# From the ocean to our kitchen table: anthropogenic particles in the edible tissue of U.S. West Coast seafood species

**DOI:** 10.3389/ftox.2024.1469995

**Published:** 2024-12-24

**Authors:** Summer D. Traylor, Elise F. Granek, Marilyn Duncan, Susanne M. Brander

**Affiliations:** ^1^ Environmental Science and Management, Portland State University, Portland, OR, United States; ^2^ Fisheries, Wildlife, and Conservation Sciences, Oregon State University, Corvallis, OR, United States

**Keywords:** contamination, lingcod, microplastics, Oregon, Pacific herring, Pacific lamprey, pink shrimp

## Abstract

Microplastics (MPs) and other anthropogenic particles (APs) are pervasive environmental contaminants found throughout marine and aquatic environments. We quantified APs in the edible tissue of black rockfish, lingcod, Chinook salmon, Pacific herring, Pacific lamprey, and pink shrimp, comparing AP burdens across trophic levels and between vessel-retrieved and retail-purchased individuals. Edible tissue was digested and analyzed under a microscope, and a subset of suspected APs was identified using spectroscopy (μFTIR). Anthropogenic particles were found in 180 of 182 individuals. Finfish contained 0.02–1.08 AP/g of muscle tissue. In pink shrimp (*Pandalus jordani*), the average AP/g was 10.68 for vessel-retrieved and 7.63 for retail-purchased samples; however, APs/g of tissue were higher in retail-purchased lingcod than vessel-retrieved lingcod, signaling possible added contamination during processing from ocean to market. Riverine young adult Pacific lamprey contained higher concentrations of APs (1 AP/g ±0.59) than ocean phase adults (0.60 AP/g ±0.80 and *p* = 0.08). Particle types identified were 82% fibers, 17% fragments, and 0.66% films. These findings suggest a need for further research into technologies and strategies to reduce microfiber pollution entering the environment.

## 1 Introduction

Anthropogenic particles (APs), a broad category of materials produced or modified by humans, include microplastics (MPs), plastics less than 5 mm in diameter at their longest dimension (Mattsson et al., 2021; Coffin et al., 2022). Environmental MPs are found in a variety of shapes, including films, foams, pellets, beads, fibers, fragments, and tire wear particles (Brander et al., 2020; Granek et al., 2020; Tamis et al., 2021), and polymer types, including polyester, polyethylene terephthalate (PET), high-density polyethylene (HDPE), and polyvinyl chloride (PVC). *Anthropogenically modified* substances refer to materials of anthropogenic origin or those that are heavily processed, like dyed cellulose textiles or poly-blends (Athey and Erdle, 2022). Since environmental particles are often a mix of MPs and other anthropogenically modified materials, we refer to APs throughout the paper, a term used by many researchers in the field (Gao et al., 2023; Adams et al., 2021).

To date, APs have been found in a variety of environmental media, including air, fresh and marine waters, sediment, wastewater, and organisms (Crawford and Quinn, 2017; Granek et al., 2022; Thornton Hampton et al., 2022). Pathways include microfibers (MFs) shed from laundering clothing (Galvão et al., 2020), MP beads from personal care products (Sun et al., 2020), and tire wear particles resulting from tire degradation (Goßmann et al., 2021; Siddiqui et al., 2022). APs can be transported aerially by wind (Brahney et al., 2020), into freshwater sources via wastewater treatment plants (WWTPs) and urban runoff (Horton et al., 2017), and into the ocean via rivers, WWTP effluent, and degradation of plastic litter (Cole et al., 2011). APs are abundant in terrestrial, freshwater, and marine environments (Wang et al., 2021) and have been found in the bodies of aquatic organisms across trophic levels, including cetaceans, avifauna, fishes, bivalves, and zooplankton (Currie et al., 2017; Romeo et al., 2015; Tanaka et al., 2019), as well as in human blood, tissue, and organs (Ragussa et al., 2021, 2022).

APs are manufactured with an array of chemicals and can also adsorb substances from the environment (Qi et al., 2021). Chemical additives used in plastic production, such as per and poly-fluorinated compounds (PFAS), phthalates, and colorants, can leach from plastics into water and body tissues over time (Sait et al., 2021; Wang et al., 2020). AP ingestion and adhesion can cause physical damage when internalized by marine organisms (Qiao et al., 2019) and lead to the transfer of constituent or associated chemicals to bodily tissues after ingestion (Tanaka et al., 2020). Gut damage (Qiao et al., 2019), adverse immune response (Sharifinia et al., 2020), protein and enzyme changes (Trestrail et al., 2021), stress response (Lanctôt et al., 2020), oxidative stress (Solomando et al., 2020), and false satiation or food dilution (Mallik et al., 2021) can result from AP exposure.

Despite the array of studies on AP ingestion across diverse species (Barboza et al., 2018; Carbery et al., 2018; Lusher et al., 2017), most studies to date have focused on bivalves in their entirety or the gastrointestinal tract of fish and crustacean species, leaving large gaps in our understanding of AP contamination in the tissue of commercially valuable finfish consumed by humans (Baechler et al., 2019; Dawson et al., 2021; Ferrante et al., 2022; Munno et al., 2021; Qiao et al., 2019; Rochman et al., 2015). However, Akoueson et al. (2020) found 0.50–1 AP/g of tissue in four finfish species collected from Scotland and Argentina, and a study of three Portuguese finfish found an average of 0.054 AP/g in dorsal muscle tissue (Barboza et al., 2020). These represent a limited number of species, geographies, habitats, and trophic levels and generate questions regarding baseline microplastic concentrations in finfish and crustaceans from different regions and trophic levels. Additionally, no known studies to date have examined the consumer source (vessel versus retail) of seafood and its relationship with AP abundance in seafood.

Oregon boasts numerous commercial, recreational, and traditional fisheries, an important part of the state’s coastal economy and fishing culture (Harte et al., 2008; Richerson et al., 2020; Robison, 2022; Sjostrom et al., 2021). There is growing interest in MP regulation and research in Oregon; however, due to the geographic variability in AP distribution and morphology, policymakers have expressed a need for further site-specific APs to guide decision-making (Das et al., 2021). Only two studies in Oregon have identified APs in consumed species (bivalves; Baechler et al., 2020; rockfish, Lasdin et al., 2023), and five studies have examined transport pathways and environmental abundance (Kapp and Yeatman, 2018; Murray et al., 2018; Talbot et al., 2022; Valine et al., 2020; Torres et al., 2023). There are no published studies on AP occurrence in the edible tissues of finfish and crustaceans in Oregon, yet such studies are important to catalyze policy-making. Across many of these studies performed in Oregon, the majority of particles identified were microfibers (Baechler et al., 2020; Lasdin et al., 2023; Torres et al., 2023).

This study aims to inform AP policy decisions by contributing to the research on AP contamination in Oregon finfish and shellfish and understand variation across trophic levels and feeding modes, as well as whether AP contamination differs across points in their pathway to consumers. Black rockfish, lingcod, Chinook salmon, Pacific herring, Pacific lamprey, and pink shrimp were selected based on their economic importance to Oregon’s commercial fisheries, their historical and cultural significance to indigenous cultures and other people in Oregon, and their variability in trophic position and feeding modes (Table 1). We examined AP contamination in individuals harvested from Oregon coastal waters, assessing differences between those obtained directly after being caught on National Oceanic and Atmospheric Administration (NOAA) or Oregon Department of Fish and Wildlife (ODFW) vessels and those caught on commercial vessels but purchased at retail markets to understand AP contamination entry. We hypothesized higher AP concentrations in riverine than oceanic stages of lamprey and in retail-purchased rather than vessel-caught individuals due to their increased exposure to plastic during seafood processing. We also predicted higher concentrations of APs in lower trophic level organisms based on the existing literature (Walkinshaw et al., 2020a).

**TABLE 1:** (Panel A) Species names (common and scientific), rationale for sampling, 2020 commercial landings in Oregon, and number of individuals collected per species by source. One individual equated to one sample. (Panel B) Study species clockwise from top left: Chinook salmon (*Oncorhynchus tshawytscha*), lingcod (*Ophiodon elongatus*), black rockfish (*Sebastes melanops*), pink shrimp (*Pandalus jordani*), Pacific lamprey (*Entosphenus tridentatus*), and Pacific herring (*Clupea pallasii)*.

(A)					
Species	Scientific name	Rationale	2020 landings in lbs from Oregon (data source)	Retail	Vessel-retrieved
Pink Shrimp	*Pandalus jordani*	• Feeding mode (filter-feeding near surface) • Largest fishery hosted in Oregon • Low trophic level	7,000,000 (ODFW)	30	30
Black rockfish	*Sebastes melanops*	• Habitat and proximity to pollution sources (near-shore bottom feeding fish) • Popularity among consumers • Accessibility for consumers • Mid-trophic level	222,667 (NOAA Fisheries)	30	12
Lingcod	*Ophiodon elongatus*	• Feeding mode (large range of prey) • Popularity among sport fishers and consumers • Non-migratory • Mid-trophic level	596,350 (NOAA Fisheries)	30	N/A
Riverine juvenile lamprey	*Entosphenus tridentatus*	• Culturally important to indigenous people of West Coast • Population pressures • Feeding mode (host feeding and filter feeding) • Mid-trophic level		N/A	15
Adult ocean-phase lamprey			98 (ODFW)		10
Pacific herring	*Clupea pallasii*	• Popularity among consumers • Habitat and proximity to pollution sources (near-shore shoaling fish) • Low-mid trophic level	72,532 (NOAA Fisheries)	N/A	15
Chinook	*Oncorhynchus tshawytscha*	• Popularity among consumers • Culturally important to indigenous people of West Coast • Population pressures • High trophic level	1,140,009 (ODFW)	N/A	10
**(B)** 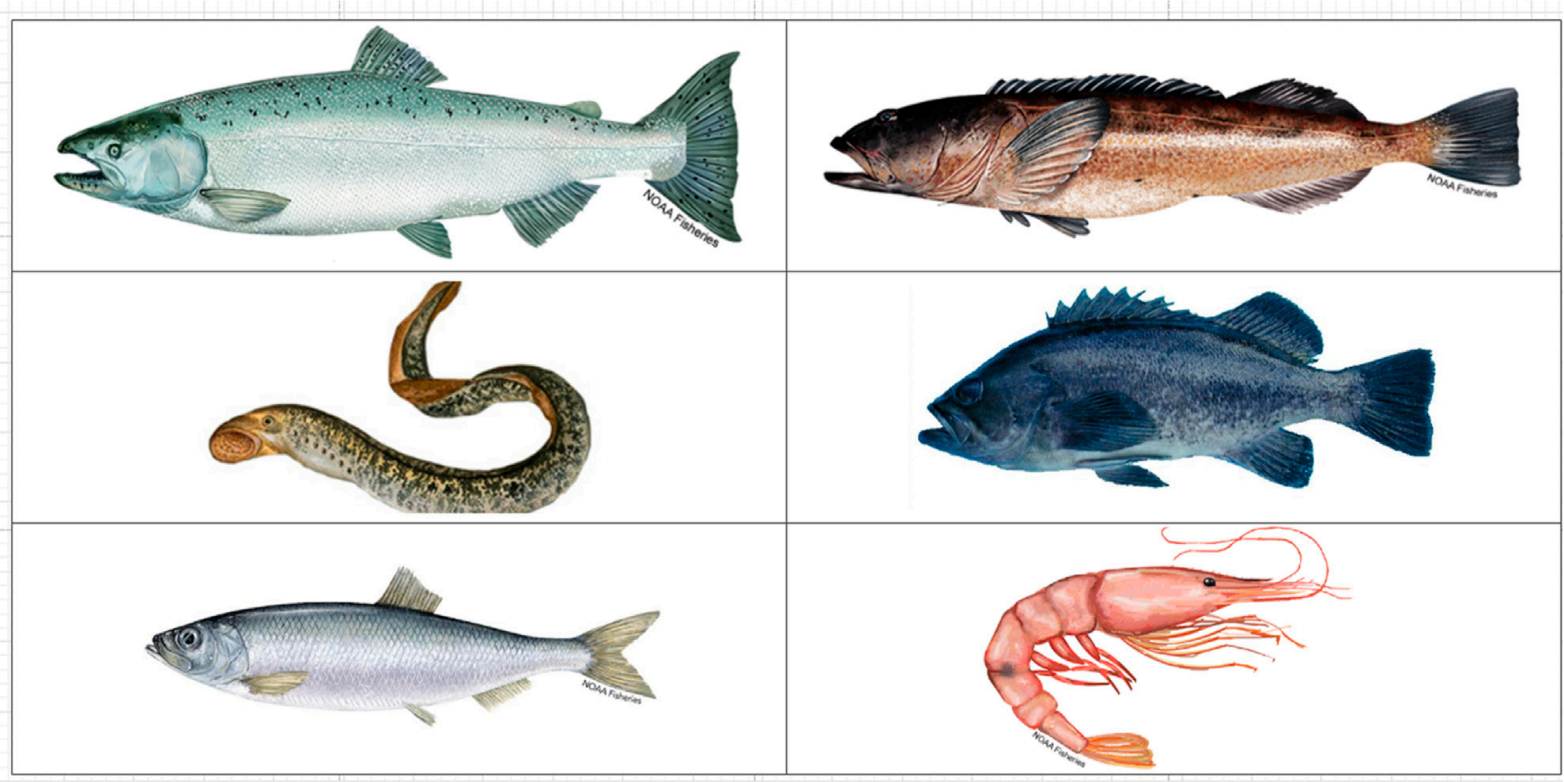					

Photo credits: Chinook salmon, lingcod, pink shrimp, Pacific herring (NOAA Fisheries), black rockfish (ODFW), and lamprey (North Carolina Wildlife Resource Commission).

## 2 Materials and methods

### 2.1 Sample collection

Five finfish and one shellfish species (Table 1) were collected from one or two sources: vessel-retrieved or/and retail-purchased from Oregon waters (see Supplementary Appendix SA1); the numbers from each source, categorized by species, are provided in Table 1. “Vessel-retrieved individuals” were whole-body fish or crustaceans caught by a fishing vessel in Oregon waters, the majority by the National Oceanic and Atmospheric Administration’s Northwest Fisheries Science Center Observers program during the 2021–2022 collection season, with the exception of riverine and ocean-phase Pacific lamprey that were collected during the 2017–2018 season (due to constraints imposed by the COVID-19 pandemic). Retail-purchased individuals were either fish fillets (finfish) or gutted shrimp purchased from a supermarket or seafood vendor. For one finfish species, lingcod, and the crustacean species, pink shrimp, samples were acquired from both fishing vessels (NOAA) and retail market (seafood counters at grocery stores), the source most accessible to the general public, and analyzed. Pacific lamprey species, listed on Oregon’s threatened and endangered species list, were acquired from ODFW and collected under permit.

Vessel-retrieved individuals were humanely euthanized by placing them in ice water baths, then double-wrapped in aluminum foil, and placed whole in plastic bags (IACUC-37, 2022). These individuals were kept frozen during transport to the Applied Coastal Ecology (ACE) Laboratory at Portland State University. Retail-purchased individuals (fillets and shelled shrimp) were purchased and transported to the ACE Laboratory as packaged by the retail store for a typical consumer. The packaging material included plastic-lined butcher paper, plastic takeout containers, and plastic freezer bags.

### 2.2 Sample processing

A total of 182 samples (122 finfish and 60 crustaceans) were collected from the Oregon coast and Oregon retail markets in 2021, while Pacific lamprey samples, collected in 2017, were frozen for later analysis.

For vessel-retrieved individuals, biological measurements, including full body length (in) and weight (g), and, if available, muscle tissue length (in) and weight (g) were recorded for all individuals. Whole-body individuals were dissected from behind the pectoral fin to the caudal fin to extract a filet of tissue corresponding to the parts of the animal typically consumed by humans.

For vessel-retrieved lingcod and Chinook salmon, approximately 220 g of muscle tissue (but ranging from 71–702 g) were randomly dissected from the front, middle, and end of the muscle tissue; for retail-purchased black rockfish and lingcod, filets were rinsed and then 32–133 (mean = 88) g of tissue were randomly dissected from the fillet. For shrimp (vessel and retail) and herring, individuals were rinsed and gutted; then all muscle tissue was dissected as all individuals sampled (75) were under 125 g (range 0.1–28 g). On average, vessel pink shrimp were 2 g (0.18–5.4 g), retail pink shrimp were 3.7 g (0.16–28 g), and vessel pacific herring were 10.77 g (3.5–19). For lamprey, riverine juveniles were defined and headed, and a small amount of muscle tissue was digested; for ocean-phase adult lamprey, we received and digested sections of muscle tissue (range 3–21 g) (see Supplementary Appendix Table SA1).

Dissected tissue from each individual was placed in its own 250 mL beaker and covered with a watch glass. Each sample was digested using a 10% potassium hydroxide (KOH) solution heated for 24–48 h at 40 C (except lamprey = 60 C), as outlined by Baechler et al. (2020), although density separation was not needed. A second digestion was needed for all lamprey samples. After digestion, the samples were vacuum-filtered through a 20-micron brass sieve (Hogentogler) to collect APs and remove the remaining liquefied tissue. The samples were then rinsed from the sieve into a vacuum filtration apparatus (Millipore Sigma) using a 47 mm diameter x 10-micron polycarbonate filter (10 μm, Millipore Sigma). Filters were then enclosed in PetriSlides (47 mm, Millipore Sigma).

### 2.3 Anthropogenic particle enumeration

Filters were examined to enumerate and measure APs using a Leica ICC50 HD with LAS V4.13 software and a ZEISS Primostar 3 with Labscope v3.3 software. Filters were examined under ×40 and ×10 magnification (depending on AP size) and photographed. Suspected APs were counted following the method outlined by Lusher et al. (2020) and classified based on their morphology, color, maximum width, and maximum length. Fiber bundles were separated into individual fibers when possible and classified accordingly. When fiber bundles could not be separated, the ends of fibers were used to count the total number of fibers in the bundle.

### 2.4 Particle characterization

A subsample of suspected plastics (n = 209, 10% of total suspected APs encountered) was sent to the Ecotox and Environmental Stress Laboratory at Oregon State University to undergo micro-Fourier transform infrared (μFTIR) spectroscopy analysis to identify specific polymers and validate total counts. Six to fifteen suspected APs (depending on the number of individuals per species) were randomly selected from each species group and analyzed via FTIR spectroscopy. OpenSpecy (Cowger et al., 2021) was used to calibrate and confirm sample material types, following methods described by Caldwell et al. (2022), Talbot et al. (2022), and Lasdin et al. (2023).

### 2.5 QA/QC

Quality control protocols were adapted from Baechler et al. (2020) and Brander et al. (2020). Pink 100% cotton laboratory coats and facemasks were used during all processing steps. Pink material was chosen to easily identify AP input from researchers in samples and controls. In addition, 100% cotton clothing was worn at all times throughout sample processing. All containers, glassware, sieves, and beakers were triple-rinsed with DI water, inverted, covered with aluminum foil, and then air-dried to minimize paper towel fibers on glass surfaces. Three DI water procedural blanks were run in conjunction with each species batch (a total of 18 blanks; 3 per every 30 samples processed) and subjected to the same digestion, sieving, and filtering protocols. Air control blanks (total of 18, three for every species batch regardless of batch size), consisting of a clean polycarbonate filter inside a clean PetriSlide, followed each species batch through the process. A 1:1 sample-to-blank ratio was used to quantify APs entering samples during microscopy. Additionally, a snorkel hood was positioned over the sample on the microscope to minimize airborne contamination.

### 2.6 Statistical analysis

Using R-studio (version 4.1.2.) statistical software, analysis of variance (ANOVA) (alpha = 0.05) was performed to test for differences in AP load among species. A Tukey honestly significant difference *post hoc* test was used to confirm significant findings. ANOVA and Welch’s two-sample t-tests were performed to test for differences between source types of the same species (pink shrimp, lingcod, and Pacific lamprey). Spearman correlation coefficients were used to evaluate relationships between the total body weight or total filet weight and overall AP tissue burden. Plots were generated using the ggplot2 and vegan packages in R.

## 3 Results

### 3.1 Anthropogenic particle characteristics

*In organisms:* Through microscope search, 1,806 suspected APs were identified across 180 of 182 individuals (averages varied drastically among species; Table 2). Fibers (1,466; 81.17%) were the most abundant, followed by fragments (332; 18.38%) and films (8; 0.44%) (see Supplementary Appendix Figure SA3B). The most common colors were blue (234; 12.95%), black (234; 12.95%), and clear or white (1,297; 71.81%) (see Supplementary Appendix Figure SA3C). The maximum length of APs ranged from 2.00–3,619 μm (mean = 911.78 μm ± 633.01), and the maximum width ranged from 0.477–1757.5 μm (mean = 26.56 μm ± 71.35) (Table 3; Supplementary Appendix Figure SA1).

**TABLE 2:** Mean particle count and mean number of particles per gram of tissue per individual (with standard error in parentheses) and the range of particles found across individuals by species.

Species	Average particle count/individual	Average # of particle/gram of tissue (AP/g) (SE)	Range of particles per individual by species
Retail			
Pink shrimp	12.6	7.6 (1.62)	1–36
Black rockfish	10	0.11 (0.02)	1–28
Lingcod	7.6	0.09 (0.009)	1–20
Vessel			
Pink shrimp	11.9	10.67 (2.26)	1–25
Riverine juvenile lamprey	8.13	1 (0.15)	3–17
Pacific herring	9.3	1.08 (0.2)	0–17
Ocean phase adult lamprey	15.9	0.60 (.25)	5–31
Lingcod	3.91	0.02 (0.006)	0–19
Chinook salmon	5.3	0.03 (0.008)	1–11

**TABLE 3:** Breakdown of material color, shape, length, and material categories identified via FTIR across species and sample collection types.

	Color				Type			Length		FTIR-type (subset of total)												
	White/clear	Black	Blue	Other	Fiber	Film	Fragment	<x size	>x size	Cardboard/cellulose	Polyester terpthalate	Fur yak bleached	Polypropyelene	Cellulose	Aramid	Viscose	Polyethylene terephthalate	Polyvinyl acetate	Low-density polyethylene	Cellophane	Natural Cellulose	Resin
Retail																						
Pink Shrimp	271	80	19	3	361	2	11	1.024	3,170.01	29	1			9		3			1		3	
Black Rockfish	134	81	81	2	245	1	54	3.795	3,243.69	2	2			9		1	9		1			1
Lingcod	155	23	50	4	185		45	1.625	3,352.95					16		3	8					
Vessel																						
Pink Shrimp	321	24	12		357	4	24	0.477	3,352.95	24	1	1	2	15		1	2			2	2	
Pacific Herring	115	8	22		134		11	3.89	3,327.61					16			1					
Lingcod	18	21	5	3	40		8	7.15	2,327.54					4								
Riverine Juvenile Pacific Lamprey	116	1	6		62	1	62	7.73	3,619.47					11		2		1				
Ocean Phase Adult Pacific Lamprey	135	5	9		70		79	1.01	3,312.4					10			2		1			
Chinook Salmon	33	7	12	1	40		13	9.433	3,188.22					12	1		1					

*In controls:* Through visual search, a total of 190 suspected APs were identified in procedural, air, and microscopy controls (see Table 4 and Supplementary Appendix Table SA2 for additional details). Fibers (160; 84.21%) were the most abundant shape found, followed by fragments (25, 13.15%) (Supplementary Appendix Figure SA3A). The most common colors were clear (132; 69.74%), blue (26; 13.68%), and black (32; 16.84%). The maximum length of all APs found in controls ranged from 29.75 to 2,969.18 μm (mean of 744.05 μm ± 515.64), and the maximum width ranged from 3.59 to 367.33 μm (mean of 29.91 μm ± 44.12) (Supplementary Appendix Figure SA1).

**TABLE 4:** APs in procedural controls, fume hood blanks, and microscope blanks, including number of pink fibers found in samples by species. Pink fibers were excluded from AP sample counts as they were presumed contamination from researcher clothing.

Species	Procedural controls (average) 1 per 10 samples	Fume hood blanks (average); 1 per 10 samples	Microscope blanks (average); 1 per 1 sample	Pink fibers in samples (total)	Pink fibers per sample (average)
Retail					
Pink shrimp	10.33	3	0.96 (n = 29)	16	(n = 0.55)
Black rockfish	3.66	2.33	0.93 (n = 23)	38	(n = 1.27)
Lingcod	5.66	3.33	0.46 (n = 14)	37	(n = 1.41)
Vessel					
Pink shrimp	9.6	4	1.13 (n = 34)	8	(n = 0.29)
Riverine juvenile lamprey	1.33	1.66	0.8 (n = 12)	2	(n = 0.13)
Pacific herring	4.66	1.33	0.6 (n = 9)	5	(n = 0.35)
Ocean phase adult lamprey	3.66	1	1.1 (n = 11)	1	(n = 0.1)
Lingcod	3	3.33	0.41 (n = 5)	3	(n = 0.27)
Chinook	1.33	2.66	1.1 (n = 11)	3	(n = 0.3)

### 3.2 Anthropogenic particles in finfish and shellfish

**FIGURE 1:**
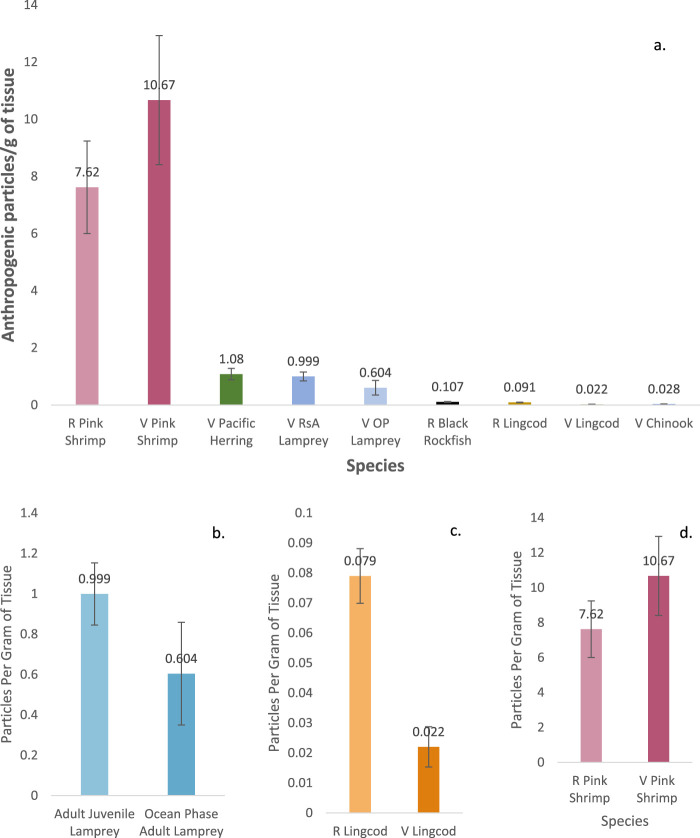
Average number (and standard error) of particles per gram of tissue found **(A)** across all species, ranked by trophic position and source type; **(B)** between riverine juvenile and ocean adult lamprey; **(C)** retail and vessel obtained lingcod; and **(D)** retail and vessel pink shrimp.

APs were found in the muscle tissue of all species of finfish and shellfish sampled; of the 182 individuals sampled, only two individuals (1%) had no APs in the section of tissue sampled (one vessel-retrieved lingcod and one vessel-retrieved herring). Among the species sampled, pink shrimp contained the most APs per individual, regardless of source type (retail: 25 (12.6 ± 1.67) per individual; vessel: 36 (11.9 ± 1.22) per individual) (Figure 1), with the most particles (36) found in a single pink shrimp weighing 4.9 g (7.35 AP/g of tissue; Tables 2, 3). Vessel-retrieved Chinook contained the smallest abundance and concentration of APs (1–11 per individual and 0.028 AP/g; Table 3). AP ranges by species and source type varied (Figure 1; Table 3). Muscle tissue weight and AP burden were inversely correlated (Spearman rank = −0.23), indicating that smaller individuals are more likely to contain APs (ANOVA: f = 9.2 and *p* = 0.0028). Biological measurements were not obtainable for retail-purchased individuals.

### 3.3 Retail-purchased and vessel-retrieved comparison

Differences in average AP/g of tissue were inconsistent between retail and vessel-caught individuals across species (lingcod and pink shrimp). Retail lingcod contained more AP/individual and more APs/g of tissue (7.33 AP/individual, 0.091 AP/g) than vessel-retrieved lingcod (3.91 AP/individual, 0.022 AP/g; Welch’s *t*-test for AP/g tissue: t = −5.1, *p* = 8.79^–5^) (Figure 1). However, retail pink shrimp contained slightly more APs/individual but fewer APs/g of tissue (12.6 AP/individual, 7.62 AP/g) than vessel-retrieved pink shrimp (11.9 AP/individual, 10.67 AP/g; Welch’s *t*-test for AP/g tissue: t = −1.2, *p* = 0.227) although the difference was not significant. An individual retail pink shrimp contained the most particles across all species and source types in a single individual (36 particles) (Figures 1C, D).

Comparing late-stage riverine juveniles and ocean phase-adult Pacific lamprey, the adults had marginally higher AP loads (15.9 versus 8.13 particles per individual) but marginally lower concentrations (0.6 AP/g versus 0.99 AP/g; Welch’s *t*-test for AP/g tissue: t = −1.32, *p* = 0.08) than juveniles (Figure 1B).

### 3.4 FTIR results

Of the 270 (∼10%) suspected APs tested using FTIR, 230 suspected APs were from individuals and 40 were from controls (detailed FTIR results in Supplementary Appendix Table SA3). OpenSpecy (Cowger et al., 2021) was used to calibrate and confirm sample material types according to methods described by Caldwell et al. (2022), Talbot et al. (2022), and Lasdin et al. (2023). In addition, 17.06% of suspected APs were fully synthetic materials, 9.47% were semi-synthetic, 8.05% were natural materials, and the overwhelming majority, 65.40%, were identified as anthropogenically modified (Supplementary Appendix Figures SA3A, SA3C). Synthetic and semi-synthetic material types included polyethylene terephthalate (PET; n = 33), polypropylene (PP; n = 3), high-density polyethene (HDPE; n = 1), low-density polyethylene (LDPE; n = 4), polyethylene vinyl acetate (PEVA; n = 1), fiberglass (n = 16), and semi-synthetic cardboard (n = 22). There was a single aramid fiber, a common material used in marine rope, flame-retardant fabrics, and military applications (Gong and Chen, 2016). Cellulose (n = 52), cotton fiber (n = 41), and cellulose acetate filter (n = 55) were the most common anthropogenically modified particles found.

### 3.5 Quality control

AP contamination in procedural controls (average: 4.80 particles), fume hood blanks (average: 2.51 particles), and microscope blanks (0.82 particles) (Table 4) was averaged for each species batch, as per previous studies (Li et al., 2015; Rochman et al., 2015; Abbasi et al., 2018), and reported in Table 4 to provide an estimate of total contamination at each sample processing step. Pink MF contamination from laboratory clothing ranged from 0.41 to 1.25 particles per sample and was excluded from all counts. Supplementary Appendix Table SA1 details AP contamination across individuals.

## 4 Discussion

### 4.1 Anthropogenic contamination characteristics

The array of AP types and colors found across the six taxa highlights the complexity of identifying AP pollution sources in aquatic environments. Li et al. (2021) described using “microplastic communities”—APs of various colors, shapes, and polymer types that accumulate together in the environment—to elicit a potential number of AP pollution sources (Li et al., 2021). Of the published studies on APs in muscle tissue that share size categories, translocation into the muscle tissue is facilitated by shape (most frequently fibers) and size although this study found slightly larger fibers and fragments than those in fish muscle tissue of other studies (Barboza et al., 2020; McIlwraith et al., 2021). This may be due to differences in protocols, AP presence in the environment at each study area, or other factors not measured in these studies. While some organisms have a tendency to ingest APs of certain colors, shapes, and sizes (Q. Chen et al., 2020; Okamoto et al., 2022), further research is needed to understand how these variables affect translocation into the muscle tissue and toxicity to organisms (Mehito et al., 2022). A recent synthesis indicated that particles <80 μm could translocate in aquatic organisms (Mehinto et al., 2022), and given that the diameter of the typical microfiber is 10–15 μm, indications are that these particles could translocate if in the proper orientation.

Of the 182 individuals sampled, only two (one vessel-retrieved Pacific herring and one vessel-retrieved lingcod) had no detected APs in their tissue although these were smaller samples 0.18–19 g of muscle (Lingcod) or full tissue (herring) sampled. The study results mirror those of Akoueson et al. (2020) and Barboza et al. (2020) and provide evidence of the widespread presence of APs in the edible tissues of Oregon’s marine and freshwater species across trophic levels and feeding modes. Pink shrimp, which filter-feed in the upper water column which contains 8–9200 AP particles/m^3^, had the highest concentrations of APs (ODFW, 2019; Curren et al., 2020; Dawson et al., 2021; Yang et al., 2021). On the other hand, Chinook salmon had the lowest concentrations, followed by black rockfish and lingcod.

Across different life stages of lamprey, we hypothesized riverine-phase juvenile lamprey, which follow the filter-feeding phase, would contain more APs than the parasitic-feeding ocean-phase adult lamprey; on an AP/g of fish basis, this hypothesis proved true, with ocean-phase adults containing 0.604 AP/g compared to riverine juveniles with 0.999 AP/g; however, since adult lamprey are larger than juveniles, they had a higher total number of APs per individual. As adults attach to a host with their mouthparts and feed primarily on the bodily fluids of host organisms (Goodman and Reid, 2017), they may inherit a portion of their APs from the bloodstream of the host organisms they feed on.

**FIGURE 2:**
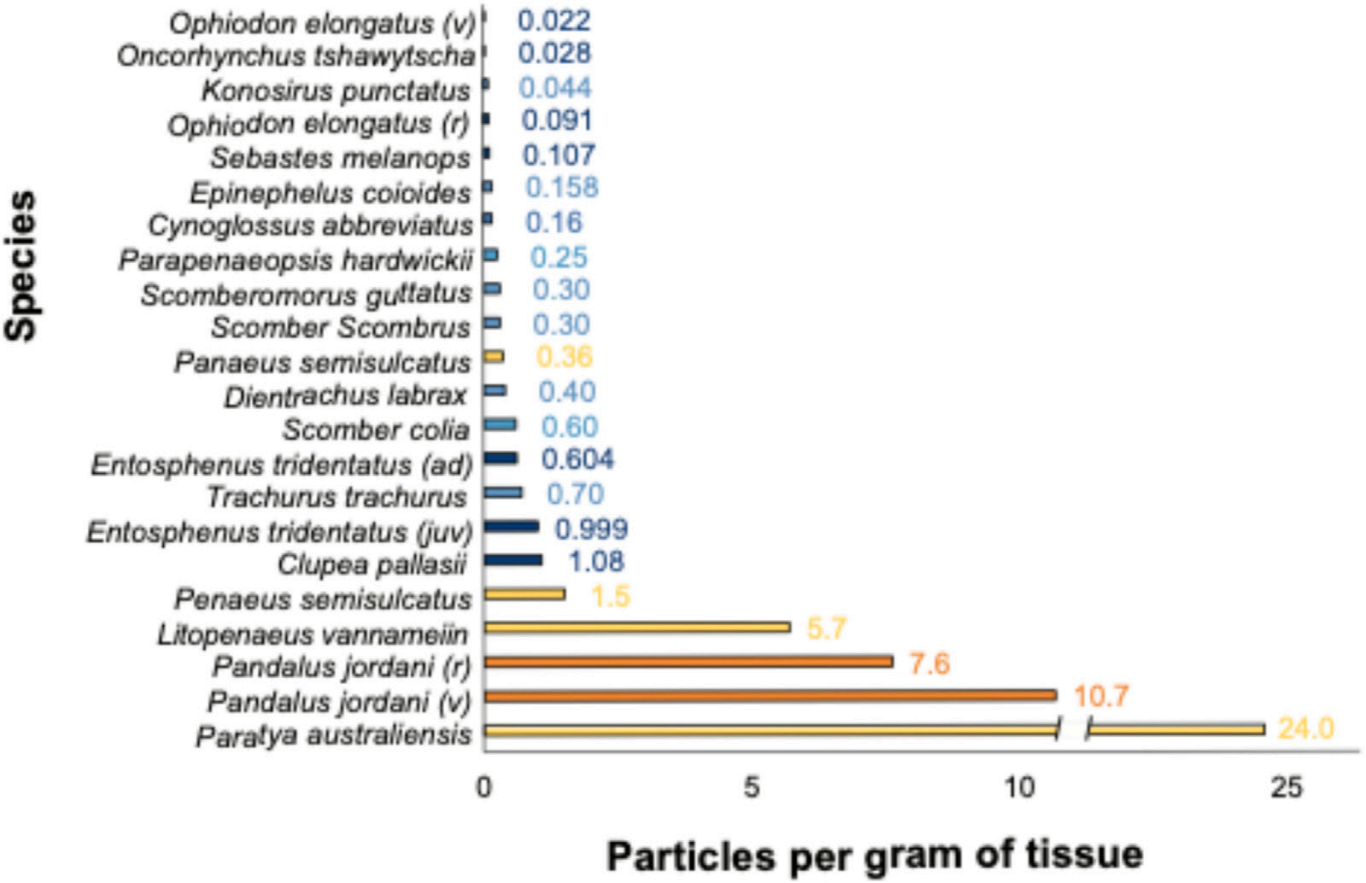
Comparison of microplastics per gram of edible shrimp (dark orange) and fish (dark blue) tissue *from this study* (see Table 1 for common names) compared with shrimp (orange) and fish (blue) from other studies around the world. v, vessel caught; r, retail; juv, juvenile; ad, adult. See Supplementary Appendix Table SA4 for sample collection locations and data sources.

This study aligns with other studies in that species across various trophic levels are exposed to AP pollution (Talley et al., 2020; F; Wang et al., 2021; Yıldız et al., 2022) and uptake and translocate particles into their tissue (e.g., Akhbarizadeh et al., 2019; Bagheri et al., 2020; Hossain et al., 2023; Nan et al., 2020; Figure 2, Supplementary Appendix Table SA2 and references therein). Furthermore, as evident from comparisons with other studies (Figure 2), we found evidence of an inverse relationship between muscle tissue AP concentration and trophic level, indicating a potential relationship between AP presence and habitat or feeding mode (see Figure 1A). Similar conclusions have been drawn by others studying trophic transfer and habitat depth (Aiguo et al., 2022; Carbery et al., 2018) and in comparisons of shellfish muscle tissue to that of higher trophic level finfish (Walkinshaw et al., 2020a).

Our study bolsters existing work on the AP muscle tissue presence, but further studies are needed to understand the mechanisms by which APs translocate into muscle tissue. Barboza et al. (2020) hypothesized that APs may transit through the bloodstream and into the muscle tissue (Barboza et al., 2020). Others hypothesize that macrophages may scavenge particles, leading to immune response and inflammation, which may facilitate translocation into the muscle tissue via cells (Leslie et al., 2022; Beijer et al., 2022).

### 4.2 Retail-purchased versus vessel-retrieved

Although other studies have found APs (MPs in the literature referenced) in retail market seafood products (Ferrante et al., 2022; Nalbone et al., 2021; Thiele et al., 2021), our results raise questions about the extent to which the retail process is a source. Our source type comparisons indicate ambiguity in retail processing as a source: AP concentrations were greater for retail than vessel-retrieved lingcod (after rinsing the surface flesh of fillets) but lower for retail than vessel-retrieved pink shrimp (after rinsing). Our results suggest that, in some cases, retail market individuals may be exposed to additional APs through processing, resulting in the incorporation of additional particles into the edible portions of seafood items. These post-mortem APs could be introduced by plastic packaging meant to preserve seafood (Dawson et al., 2021; Habib et al., 2022; Jadhav et al., 2021; Kedzierski et al., 2020). It is unclear why the retail process did not add microplastics to lingcod, as was observed for pink shrimp, indicating a need for further investigation to understand where and when AP contamination occurs post-catch.

### 4.3 Study limitations

Our sample size for the larger finfish was small, limiting the generalizability of the findings for these species. Additionally, since the study focused on species that span the US West Coast but only collected organisms from the Oregon coast, the concentrations may not represent coast-wide microplastic concentrations. However, a comparison with other studies on muscle tissue microplastic concentrations demonstrates that our results fall within the range of microplastics per gram of edible fish tissue found globally (Figure 2; Supplementary Appendix Table SA4). Future research should consider collecting the same species at various points along the West Coast to determine whether there is spatial variability in microplastic contamination from northern Washington to Southern California, particularly for the species that span the entire coast.

## 5 Implications for Oregon’s seafood producers, consumers, and threatened species

For producers and handlers of seafood, we recommend shifting to alternative packaging methods such as natural materials made from beeswax, starches, or sugars that will limit the introduction of APs into retail seafood (Chen et al., 2022; Herrmann et al., 2022; Rangaraj et al., 2021). Research and development may be needed to provide economically viable alternative products that perform similarly to plastic (Hurst-Mayr et al., in prep). For consumers, we recommend buying whole, local fish whenever possible to minimize APs introduced via plastic packaging. Regardless of the source of seafood products, individuals containing APs were found to have at least 0.3 AP/10 g of edible tissue, signaling the need for policy and other interventions to regulate APs.

Since the species we sampled are consumed by both humans and marine predators, there is a potential for biomagnification (see Torres et al., 2023). The presence of APs in edible tissues and the possibility that they are translocated from the gut or gills highlight the need for further research into the health effects of AP consumption for both aquatic organisms and humans. To date, the majority of studies identifying health effects of AP consumption by aquatic organisms, e.g., adverse cellular responses, inflammation, oxidative stress, negative impacts on growth and development, physical damage to organs, behavioral changes, adverse reproductive responses, and decreased survivorship (Lanctôt et al., 2020; Mallik et al., 2021; Qiao et al., 2019; Sharifinia et al., 2020; Solomando et al., 2020; Trestrail et al., 2021; Siddiqui et al., 2022), have focused on model species, which may under-represent the effects on wild species or did not use environmentally relevant AP concentrations, which may mis/over-represent effects.

In Oregon and the Western U.S., Pacific lamprey are listed as endangered at the state level, affected by artificial barriers to migration, poor water quality, loss of habitat, and changing ocean conditions. Moreover, Pacific lamprey are a culturally important food source for indigenous peoples of the Pacific Northwest, so consumption of Pacific lamprey is a source of AP exposure for these communities specifically (Monroe, 2013). Although APs have been found in a variety of food items across trophic levels, people who depend upon subsistence fisheries are likely ingesting fish-borne contaminants, highlighting the environmental justice issue perpetuated by AP pollution in Pacific lamprey (National Environmental Justice Advisory & Council Fish Consumption and Environmental Justice, 2002). Further research is needed to understand the baseline exposure of indigenous communities from lamprey consumption, how lamprey internalize APs relative to other seafood species, how APs may impact this already endangered species in Oregon, and how to reduce lamprey exposure. Due to the historical and systemic oppression of indigenous tribes in the U.S., the state of Oregon has a responsibility to employ larger-scale AP regulation methods to minimize exposure to AP pollution through Pacific lamprey consumption (Duffield et al., 2021). While Walkinshaw et al. (2020a) highlighted that seafood most likely does not contribute more APs than consuming other foods or drinking water, the high consumption of seafood by low income and indigenous peoples and the continued prevalence of APs detected in humans (Ibrahim et al., 2021; Jenner et al., 2022; Leslie et al., 2022; Ragusa et al., 2021) necessitates further study of the long-term exposure effects on human health (Lusher et al., 2017; Walkinshaw et al., 2020b; Coffin et al., 2022).

## 6 Implications and next steps for policymakers and researchers

Although the percentage of validated APs is only 10%, the detection of APs across all six taxa spanning multiple trophic levels and two sources confirms the need for actions to address aquatic exposures. Four potential pathways for policymakers and researchers to address APs include mitigation technology, monitoring, long-term research, and legislation.

Current AP pollution loads in the environment have reached a level irreversible by current technologies (Uzun et al., 2022). Therefore, addressing AP pollution requires mitigative approaches to reduce the flow of AP pollution into the environment or “turning off the tap” on virgin plastic production (Bergmann et al., 2022). A newer mitigative technology is laundry machine MF catchment filters. Widespread implementation of this mitigative technology or, at the very least, selective use at high-emission sites, would reduce MF pollution entering waterways (Erdle et al., 2021).

Government agencies should follow California’s lead and pursue ambient monitoring of AP in drinking water, air, and waterways (California S.B. 1,422, 2018) to inform large-scale regional policy to effectively control AP pollution. Long-term and multi-generational research studies on organismal health following exposure to AP pollution are also needed. Both anthropogenically modified and synthetic/semi-synthetic materials should be tested as the abundance of both continues to grow in the environment (Siddiqui et al., 2022; Walkinshaw et al., 2023). Ambient monitoring along the West Coast would inform concentrations for such long-term studies. Finally, the Western states should work collaboratively to create a standardized monitoring approach and, ultimately, coordinate regulatory policy for AP pollution. However, since AP environmental pollution is no longer the problem of one country or government, global policies such as the ongoing global plastics treaty negotiations and enforcement of an eventual treaty are ultimately needed to address the problem of AP pollution at its current magnitude. Unless we change our relationship with plastic and significantly reduce plastic production, we will continue to witness its negative impacts.

## Data Availability

The original contributions presented in the study are included in the article/Supplementary Material; further inquiries can be directed to the corresponding author.
