# Supplementary material for: From the ocean to our kitchen table: anthropogenic particles in the edible tissue of U.S. West Coast seafood species
Source: Front Toxicol. 2024 Dec 24;6:1469995. doi: 10.3389/ftox.2024.1469995 (PMC11703854; doi:10.3389/ftox.2024.1469995)
Supplement: Supplementary file 1 [file DataSheet1.docx]

# APPENDIX

**Species locations**

All species were confirmed either by retail market managers or fishermen, to be caught in Oregon waters (state waters or waters parallel to the Oregon coastline). Vessel retrieved lingcod were caught 17 miles off the coast of Tillamook Bay, 25 miles off the coast of Newport, 1 mile West of North Lincoln City, 2 miles W of Lincoln city, 11 -12 miles west of Winchester Bay, 3 miles off Florence. Vessel retrieved pink shrimp were caught 5 miles west of Lincoln City and 2 miles west of Garibaldi. Salmon and pacific herring were caught in open ocean along Oregon state waters and not in waters adjacent to California waters or Washington waters.

Table A: The weight, total microplastics, and microplastics per gram of tissue for each organism sampled.

| **Species** | **Sample ID** | **Weight** | **Total APs** | **APs/g tissue** | **Source** |
| --- | --- | --- | --- | --- | --- |
| Pink shrimp - vessel | PS-V-1 | 1.57 | 2 | 1.27 | vessel |
|  | PS-V-2 | 2.20 | 2 | 0.91 | vessel |
|  | PS-V-3 | 2.29 | 11 | 4.80 | vessel |
|  | PS-V-4 | 2.09 | 7 | 3.35 | vessel |
|  | PS-V-5 | 1.77 | 14 | 7.91 | vessel |
|  | PS-V-6 | 0.94 | 7 | 7.45 | vessel |
|  | PS-V-7 | 1.21 | 10 | 8.24 | vessel |
|  | PS-V-8 | 1.00 | 6 | 6.01 | vessel |
|  | PS-V-9 | 1.02 | 19 | 18.70 | vessel |
|  | PS-V-10 | 0.81 | 15 | 18.54 | vessel |
|  | PS-V-11 | 0.94 | 12 | 12.78 | vessel |
|  | PS-V-12 | 0.62 | 21 | 33.71 | vessel |
|  | PS-V-13 | 0.99 | 24 | 24.27 | vessel |
|  | PS-V-14 | 0.71 | 25 | 35.16 | vessel |
|  | PS-V-15 | 2.25 | 15 | 6.67 | vessel |
|  | PS-V-16 | 2.16 | 10 | 4.63 | vessel |
|  | PS-V-17 | 2.79 | 22 | 7.89 | vessel |
|  | PS-V-18 | 2.12 | 1 | 0.47 | vessel |
|  | PS-V-19 | 3.18 | 4 | 1.26 | vessel |
|  | PS-V-20 | 0.18 | 10 | 55.22 | vessel |
|  | PS-V-21 | 3.00 | 16 | 5.33 | vessel |
|  | PS-V-22 | 5.44 | 8 | 1.47 | vessel |
|  | PS-V-23 | 2.40 | 6 | 2.50 | vessel |
|  | PS-V-24 | 2.43 | 3 | 1.23 | vessel |
|  | PS-V-25 | 3.81 | 12 | 3.15 | vessel |
|  | PS-V-26 | 2.04 | 21 | 10.29 | vessel |
|  | PS-V-27 | 2.96 | 11 | 3.71 | vessel |
|  | PS-V-28 | 1.15 | 15 | 13.04 | vessel |
|  | PS-V-29 | 2.73 | 13 | 4.75 | vessel |
|  | PS-V-30 | 0.96 | 15 | 15.63 | vessel |
| Pink shrimp-retail | PS-S-1 | 2.66 | 25 | 9.41 | retail |
|  | PS-S-2 | 1.71 | 3 | 1.76 | retail |
|  | PS-S-3 | 0.60 | 23 | 38.66 | retail |
|  | PS-S-4 | 11.23 | 11 | 0.98 | retail |
|  | PS-S-5 | 2.52 | 4 | 1.59 | retail |
|  | PS-S-6 | 0.16 | 4 | 24.69 | retail |
|  | PS-S-7 | 2.75 | 6 | 2.18 | retail |
|  | PS-S-8 | 2.22 | 8 | 3.61 | retail |
|  | PS-S-9 | 28.32 | 15 | 0.53 | retail |
|  | PS-S-10 | 6.76 | 19 | 2.81 | retail |
|  | PS-S-11 | 11.02 | 21 | 1.91 | retail |
|  | PS-S-12 | 5.59 | 10 | 1.79 | retail |
|  | PS-S-13 | 6.61 | 13 | 1.97 | retail |
|  | PS-S-14 | 2.57 | 12 | 4.66 | retail |
|  | PS-S-15 | 4.90 | 36 | 7.35 | retail |
|  | PS-S-16 | 1.14 | 10 | 8.78 | retail |
|  | PS-S-17 | 1.46 | 31 | 21.29 | retail |
|  | PS-S-18 | 1.40 | 3 | 2.14 | retail |
|  | PS-S-19 | 2.24 | 3 | 1.34 | retail |
|  | PS-S-20 | 1.58 | 17 | 10.73 | retail |
|  | PS-S-21 | 1.33 | 2 | 1.51 | retail |
|  | PS-S-22 | 1.19 | 18 | 15.11 | retail |
|  | PS-S-23 | 1.28 | 20 | 15.61 | retail |
|  | PS-S-24 | 1.09 | 1 | 0.92 | retail |
|  | PS-S-25 | 1.61 | 2 | 1.24 | retail |
|  | PS-S-26 | 1.60 | 7 | 4.37 | retail |
|  | PS-S-27 | 1.96 | 18 | 9.16 | retail |
|  | PS-S-28 | 1.29 | 3 | 2.32 | retail |
|  | PS-S-29 | 1.13 | 14 | 12.37 | retail |
|  | PS-S-30 | 1.06 | 19 | 18.01 | retail |
| Herring | H-V-1 | 13.06 | 17 | 1.30 | vessel |
|  | H-V-2 | 6.21 | 13 | 2.10 | vessel |
|  | H-V-3 | 10.88 | 15 | 1.38 | vessel |
|  | H-V-4 | 3.52 | 0 | 0.00 | vessel |
|  | H-V-5 | 15.60 | 8 | 0.51 | vessel |
|  | H-V-6 | 5.36 | 12 | 2.24 | vessel |
|  | H-V-7 | 15.10 | 6 | 0.40 | vessel |
|  | H-V-8 | 7.49 | 7 | 0.93 | vessel |
|  | H-V-9 | 7.24 | 10 | 1.38 | vessel |
|  | H-V-10 | 6.33 | 17 | 2.69 | vessel |
|  | H-V-11 | 19.16 | 14 | 0.73 | vessel |
|  | H-V-12 | 8.52 | 7 | 0.82 | vessel |
|  | H-V-13 | 10.07 | 10 | 0.99 | vessel |
|  | H-V-14 | 13.30 | 7 | 0.53 | vessel |
|  | H-V-15 | 19.78 | 6 | 0.30 | vessel |
| Riverine Juvenile Pacific Lamprey | LAJ-1 | 8.25 | 14 | 1.70 | vessel |
|  | LAJ-2 | 5.19 | 7 | 1.35 | vessel |
|  | LAJ-3 | 4.85 | 6 | 1.24 | vessel |
|  | LAJ-4 | 3.63 | 8 | 2.20 | vessel |
|  | LAJ-5 | 11.67 | 11 | 0.94 | vessel |
|  | LAJ-6 | 10.08 | 4 | 0.40 | vessel |
|  | LAJ-7 | 14.23 | 6 | 0.42 | vessel |
|  | LAJ-8 | 3.04 | 5 | 1.64 | vessel |
|  | LAJ-9 | 9.36 | 7 | 0.75 | vessel |
|  | LAJ-10 | 14.85 | 7 | 0.47 | vessel |
|  | LAJ-11 | 10.28 | 8 | 0.78 | vessel |
|  | LAJ-12 | 12.35 | 9 | 0.73 | vessel |
|  | LAJ-13 | 12.50 | 3 | 0.24 | vessel |
|  | LAJ-14 | 10.24 | 17 | 1.66 | vessel |
|  | LAJ-15 | 21.16 | 10 | 0.47 | vessel |
| Ocean Phase Adult Pacific Lamprey | LOP-1 | 43.17 | 31 | 0.72 | vessel |
|  | LOP-2 | 47.33 | 5 | 0.11 | vessel |
|  | LOP-3 | 46.23 | 21 | 0.45 | vessel |
|  | LOP-4 | 43.20 | 11 | 0.25 | vessel |
|  | LOP-5 | 59.17 | 11 | 0.19 | vessel |
|  | LOP-6 | 75.41 | 19 | 0.25 | vessel |
|  | LOP-7 | 50.60 | 11 | 0.22 | vessel |
|  | LOP-8 | 11.06 | 5 | 0.45 | vessel |
|  | LOP-9 | 26.03 | 15 | 0.58 | vessel |
|  | LOP-10 | 10.61 | 30 | 2.83 | vessel |
| Black rockfish | BR-S-1 | 65.08 | 2 | 0.03 | retail |
|  | BR-S-2 | 74.21 | 3 | 0.04 | retail |
|  | BR-S-3 | 125.41 | 9 | 0.07 | retail |
|  | BR-S-4 | 78.40 | 2 | 0.03 | retail |
|  | BR-S-5 | 129.35 | 14 | 0.11 | retail |
|  | BR-S-6 | 76.32 | 2 | 0.03 | retail |
|  | BR-S-7 | 133.41 | 15 | 0.11 | retail |
|  | BR-S-8 | 82.85 | 9 | 0.11 | retail |
|  | BR-S-9 | 117.52 | 28 | 0.24 | retail |
|  | BR-S-10 | 80.38 | 7 | 0.09 | retail |
|  | BR-S-11 | 79.86 | 7 | 0.09 | retail |
|  | BR-S-12 | 69.12 | 18 | 0.26 | retail |
|  | BR-S-13 | 84.81 | 2 | 0.02 | retail |
|  | BR-S-14 | 88.96 | 16 | 0.18 | retail |
|  | BR-S-15 | 83.53 | 21 | 0.25 | retail |
|  | BR-S-16 | 65.96 | 9 | 0.14 | retail |
|  | BR-S-17 | 83.94 | 10 | 0.12 | retail |
|  | BR-S-18 | 82.66 | 8 | 0.10 | retail |
|  | BR-S-19 | 95.42 | 13 | 0.14 | retail |
|  | BR-S-20 | 91.95 | 25 | 0.27 | retail |
|  | BR-S-21 | 106.10 | 5 | 0.05 | retail |
|  | BR-S-22 | 85.28 | 2 | 0.02 | retail |
|  | BR-S-23 | 83.44 | 1 | 0.01 | retail |
|  | BR-S-24 | 106.51 | 2 | 0.02 | retail |
|  | BR-S-25 | 98.51 | 28 | 0.28 | retail |
|  | BR-S-26 | 99.84 | 16 | 0.16 | retail |
|  | BR-S-27 | 95.96 | 5 | 0.05 | retail |
|  | BR-S-28 | 92.57 | 6 | 0.06 | retail |
|  | BR-S-29 | 101.15 | 7 | 0.07 | retail |
|  | BR-S-30 | 113.16 | 8 | 0.07 | retail |
| Lingcod- retail | LC-S-1 | 114.19 | 20 | 0.18 | retail |
|  | LC-S-2 | 103.82 | 10 | 0.10 | retail |
|  | LC-S-3 | 89.56 | 3 | 0.03 | retail |
|  | LC-S-4 | 72.12 | 3 | 0.04 | retail |
|  | LC-S-5 | 110.29 | 14 | 0.13 | retail |
|  | LC-S-6 | 107.46 | 10 | 0.09 | retail |
|  | LC-S-7 | 83.23 | 14 | 0.17 | retail |
|  | LC-S-8 | 100.40 | 5 | 0.05 | retail |
|  | LC-S-9 | 97.90 | 6 | 0.06 | retail |
|  | LC-S-10 | 32.99 | 7 | 0.21 | retail |
|  | LC-S-11 | 59.14 | 5 | 0.08 | retail |
|  | LC-S-12 | 105.50 | 7 | 0.07 | retail |
|  | LC-S-13 | 70.17 | 3 | 0.04 | retail |
|  | LC-S-14 | 81.28 | 15 | 0.18 | retail |
|  | LC-S-15 | 61.50 | 8 | 0.13 | retail |
|  | LC-S-16 | 73.72 | 2 | 0.03 | retail |
|  | LC-S-17 | 74.76 | 5 | 0.07 | retail |
|  | LC-S-18 | 80.76 | 14 | 0.17 | retail |
|  | LC-S-19 | 113.65 | 7 | 0.06 | retail |
|  | LC-S-20 | 94.60 | 8 | 0.08 | retail |
|  | LC-S-21 | 76.73 | 4 | 0.05 | retail |
|  | LC-S-22 | 101.70 | 8 | 0.08 | retail |
|  | LC-S-23 | 75.52 | 6 | 0.08 | retail |
|  | LC-S-24 | 94.62 | 11 | 0.12 | retail |
|  | LC-S-25 | 84.54 | 8 | 0.09 | retail |
|  | LC-S-26 | 66.24 | 7 | 0.11 | retail |
|  | LC-S-27 | 92.09 | 9 | 0.10 | retail |
|  | LC-S-28 | 83.84 | 4 | 0.05 | retail |
|  | LC-S-29 | 67.77 | 5 | 0.07 | retail |
|  | LC-S-30 | 87.70 | 2 | 0.02 | retail |
| Lingcod -vessel | LC-V-1 | 220.50 | 5 | 0.02 | vessel |
|  | LC-V-2 | 264.67 | 7 | 0.03 | vessel |
|  | LC-V-3 | 250.79 | 1 | 0.00 | vessel |
|  | LC-V-4 | 253.80 | 19 | 0.07 | vessel |
|  | LC-V-5 | 280.23 | 1 | 0.00 | vessel |
|  | LC-V-6 | 214.87 | 4 | 0.02 | vessel |
|  | LC-V-7 | 109.99 | 0 | 0.00 | vessel |
|  | LC-V-8 | 97.17 | 1 | 0.01 | vessel |
|  | LC-V-9 | 127.22 | 1 | 0.01 | vessel |
|  | LC-V-10 | 76.59 | 3 | 0.04 | vessel |
|  | LC-V-11 | 71.81 | 4 | 0.06 | vessel |
|  | LC-V-12 | 147.25 | 1 | 0.01 | vessel |
| Chinook salmon | C-V-1 | 702.55 | 10 | 0.01 | vessel |
|  | C-V-2 | 518.82 | 11 | 0.02 | vessel |
|  | C-V-3 | 689.55 | 6 | 0.01 | vessel |
|  | C-V-4 | 190.52 | 1 | 0.01 | vessel |
|  | C-V-5 | 190.97 | 4 | 0.02 | vessel |
|  | C-V-6 | 102.50 | 8 | 0.08 | vessel |
|  | C-V-7 | 77.12 | 5 | 0.06 | vessel |
|  | C-V-8 | 131.50 | 5 | 0.04 | vessel |
|  | C-V-9 | 100.00 | 1 | 0.01 | vessel |
|  | C-V-10 | 81.00 | 2 | 0.02 | vessel |

Table B. For each individual dissected, the number of microparticles in the tissue, in all three procedural controls, in all three fume hood blanks for that species, in air control blanks per sample during microscopy.

| **Species** | **Individual #** | **Number of APs Found in Tissues** | **Average Number of APs found in Procedural Controls (3 controls per species)** | **Average Number of APs found in Fumehood Blanks (3 blanks per species)** | **Total Number of APs found in Air Control Blanks (1:1)** |
| --- | --- | --- | --- | --- | --- |
| **Pink Shrimp-retail** | 1 | 25 | 10.33333333 | 3 | 0 |
|  | 2 | 3 |  |  | 0 |
|  | 3 | 23 |  |  | 0 |
|  | 4 | 11 |  |  | 2 |
|  | 5 | 4 |  |  | 0 |
|  | 6 | 4 |  |  | 0 |
|  | 7 | 6 |  |  | 1 |
|  | 8 | 8 |  |  | 0 |
|  | 9 | 15 |  |  | 1 |
|  | 10 | 19 |  |  | 2 |
|  | 11 | 21 |  |  | 6 |
|  | 12 | 10 |  |  | 1 |
|  | 13 | 13 |  |  | 0 |
|  | 14 | 12 |  |  | 1 |
|  | 15 | 36 |  |  | 3 |
|  | 16 | 10 |  |  | 0 |
|  | 17 | 31 |  |  | 0 |
|  | 18 | 3 |  |  | 0 |
|  | 19 | 3 |  |  | 0 |
|  | 20 | 17 |  |  | 3 |
|  | 21 | 2 |  |  | 0 |
|  | 22 | 18 |  |  | 3 |
|  | 23 | 20 |  |  | 1 |
|  | 24 | 1 |  |  | 0 |
|  | 25 | 2 |  |  | 0 |
|  | 26 | 7 |  |  | 0 |
|  | 27 | 18 |  |  | 0 |
|  | 28 | 3 |  |  | 0 |
|  | 29 | 14 |  |  | 1 |
|  | 30 | 19 |  |  | 0 |
| **Pink Shrimp- vessel** | 1 | 2 | 9.666666667 | 2.333333333 | 4 |
|  | 2 | 2 |  |  | 0 |
|  | 3 | 11 |  |  | 5 |
|  | 4 | 7 |  |  | 0 |
|  | 5 | 14 |  |  | 3 |
|  | 6 | 7 |  |  | 0 |
|  | 7 | 10 |  |  | 0 |
|  | 8 | 6 |  |  | 0 |
|  | 9 | 19 |  |  | 1 |
|  | 10 | 15 |  |  | 0 |
|  | 11 | 12 |  |  | 0 |
|  | 12 | 21 |  |  | 0 |
|  | 13 | 24 |  |  | 1 |
|  | 14 | 25 |  |  | 0 |
|  | 15 | 15 |  |  | 0 |
|  | 16 | 10 |  |  | 0 |
|  | 17 | 22 |  |  | 1 |
|  | 18 | 1 |  |  | 1 |
|  | 19 | 4 |  |  | 2 |
|  | 20 | 10 |  |  | 3 |
|  | 21 | 16 |  |  | 0 |
|  | 22 | 8 |  |  | 3 |
|  | 23 | 6 |  |  | 0 |
|  | 24 | 3 |  |  | 0 |
|  | 25 | 12 |  |  | 0 |
|  | 26 | 21 |  |  | 2 |
|  | 27 | 11 |  |  | 5 |
|  | 28 | 15 |  |  | 1 |
|  | 29 | 13 |  |  | 0 |
|  | 30 | 15 |  |  | 0 |
| **Pacific Herring** | 1 | 17 | 4.666666667 | 1.333333333 | 1 |
|  | 2 | 13 |  |  | 0 |
|  | 3 | 15 |  |  | 1 |
|  | 4 | 0 |  |  | 0 |
|  | 5 | 8 |  |  | 0 |
|  | 6 | 12 |  |  | 0 |
|  | 7 | 6 |  |  | 0 |
|  | 8 | 7 |  |  | 1 |
|  | 9 | 10 |  |  | 0 |
|  | 10 | 17 |  |  | 0 |
|  | 11 | 14 |  |  | 0 |
|  | 12 | 7 |  |  | 1 |
|  | 13 | 10 |  |  | 1 |
|  | 14 | 7 |  |  | 0 |
|  | 15 | 6 |  |  | 1 |
| **Riverine Juvenile Lamprey** | 1 | 14 | 1.333333333 | 1.666666667 | 0 |
|  | 2 | 7 |  |  | 0 |
|  | 3 | 6 |  |  | 3 |
|  | 4 | 8 |  |  | 0 |
|  | 5 | 11 |  |  | 1 |
|  | 6 | 4 |  |  | 0 |
|  | 7 | 6 |  |  | 0 |
|  | 8 | 5 |  |  | 0 |
|  | 9 | 7 |  |  | 3 |
|  | 10 | 7 |  |  | 0 |
|  | 11 | 8 |  |  | 0 |
|  | 12 | 9 |  |  | 0 |
|  | 13 | 3 |  |  | 0 |
|  | 14 | 17 |  |  | 1 |
|  | 15 | 10 |  |  | 3 |
| **Ocean Phase Adult Lamprey** | 1 | 31 | 3.666666667 | 1 | 3 |
|  | 2 | 5 |  |  | 0 |
|  | 3 | 21 |  |  | 0 |
|  | 4 | 11 |  |  | 0 |
|  | 5 | 11 |  |  | 0 |
|  | 6 | 19 |  |  | 0 |
|  | 7 | 11 |  |  | 3 |
|  | 8 | 5 |  |  | 0 |
|  | 9 | 15 |  |  | 2 |
|  | 10 | 30 |  |  | 0 |
| **Black Rockfish** | 1 | 2 | 3.666666667 | 2.333333333 | 0 |
|  | 2 | 3 |  |  | 0 |
|  | 3 | 9 |  |  | 0 |
|  | 4 | 2 |  |  | 1 |
|  | 5 | 14 |  |  | 3 |
|  | 6 | 2 |  |  | 0 |
|  | 7 | 15 |  |  | 0 |
|  | 8 | 9 |  |  | 2 |
|  | 9 | 28 |  |  | 5 |
|  | 10 | 7 |  |  | 2 |
|  | 11 | 7 |  |  | 0 |
|  | 12 | 18 |  |  | 0 |
|  | 13 | 2 |  |  | 0 |
|  | 14 | 16 |  |  | 0 |
|  | 15 | 21 |  |  | 1 |
|  | 16 | 9 |  |  | 1 |
|  | 17 | 10 |  |  | 1 |
|  | 18 | 8 |  |  | 0 |
|  | 19 | 13 |  |  | 0 |
|  | 20 | 25 |  |  | 1 |
|  | 21 | 5 |  |  | 0 |
|  | 22 | 2 |  |  | 0 |
|  | 23 | 1 |  |  | 0 |
|  | 24 | 2 |  |  | 0 |
|  | 25 | 28 |  |  | 3 |
|  | 26 | 16 |  |  | 2 |
|  | 27 | 5 |  |  | 0 |
|  | 28 | 6 |  |  | 0 |
|  | 29 | 7 |  |  | 0 |
|  | 30 | 8 |  |  | 0 |
| **Lingcod-retail** | 1 | 20 | 5.666666667 | 3.333333333 | 0 |
|  | 2 | 10 |  |  | 0 |
|  | 3 | 3 |  |  | 1 |
|  | 4 | 3 |  |  | 1 |
|  | 5 | 14 |  |  | 3 |
|  | 6 | 10 |  |  | 2 |
|  | 7 | 14 |  |  | 2 |
|  | 8 | 5 |  |  | 0 |
|  | 9 | 6 |  |  | 0 |
|  | 10 | 7 |  |  | 0 |
|  | 11 | 5 |  |  | 0 |
|  | 12 | 7 |  |  | 0 |
|  | 13 | 3 |  |  | 0 |
|  | 14 | 15 |  |  | 0 |
|  | 15 | 8 |  |  | 0 |
|  | 16 | 2 |  |  | 0 |
|  | 17 | 5 |  |  | 0 |
|  | 18 | 14 |  |  | 1 |
|  | 19 | 7 |  |  | 1 |
|  | 20 | 8 |  |  | 0 |
|  | 21 | 4 |  |  | 0 |
|  | 22 | 8 |  |  | 2 |
|  | 23 | 6 |  |  | 1 |
|  | 24 | 11 |  |  | 0 |
|  | 25 | 8 |  |  | 0 |
|  | 26 | 7 |  |  | 0 |
|  | 27 | 9 |  |  | 0 |
|  | 28 | 4 |  |  | 0 |
|  | 29 | 5 |  |  | 0 |
|  | 30 | 2 |  |  | 0 |
| **Lingcod- vessel** | 1 | 5 | 3 | 3.333333333 | 1 |
|  | 2 | 7 |  |  | 0 |
|  | 3 | 1 |  |  | 0 |
|  | 4 | 19 |  |  | 0 |
|  | 5 | 1 |  |  | 0 |
|  | 6 | 4 |  |  | 3 |
|  | 7 | 0 |  |  | 0 |
|  | 8 | 1 |  |  | 0 |
|  | 9 | 1 |  |  | 0 |
|  | 10 | 3 |  |  | 0 |
|  | 11 | 4 |  |  | 0 |
|  | 12 | 1 |  |  | 0 |
| **Chinook salmon** | 1 | 10 | 1.333333333 | 2.666666667 | 3 |
|  | 2 | 11 |  |  | 0 |
|  | 3 | 6 |  |  | 0 |
|  | 4 | 1 |  |  | 0 |
|  | 5 | 4 |  |  | 0 |
|  | 6 | 8 |  |  | 5 |
|  | 7 | 5 |  |  | 0 |
|  | 8 | 5 |  |  | 0 |
|  | 9 | 1 |  |  | 0 |
|  | 10 | 2 |  |  | 0 |

Table C. FTIR Data table including sample name and number, AP morphology and color, micro ATR and OpenSpecy results and match percentages, and final categorization.

| **Site** | **Sample #** | **Morph-ology** | **Particle Color** | **Micro ATR Results Top 10** | **Match #** | **OpenSpecy Micro ATR Results Top 5** | **Match #** | **Categoriz-ation** |
| --- | --- | --- | --- | --- | --- | --- | --- | --- |
| PS-V | 3a | Fiber | Clear White | Wood mahagoni | 85.21 | Cardboard/cellulose | 0.98 | Natural |
|  |  |  |  | Wood pine | 84.05 |  |  |  |
|  |  |  |  | Mask 128S GeATR | 81.91 | Papercup cellulosic | 0.98 |  |
|  |  |  |  | Fibre poplar down | 79.77 |  |  |  |
|  |  |  |  | Fibre grass | 78.62 | Cellulose | 0.95 |  |
|  |  |  |  | Fibre poplar down | 78.50 |  |  |  |
|  |  |  |  | Cellulose wipe | 77.94 | Cellulose | 0.94 |  |
|  |  |  |  | Cellulose | 77.73 |  |  |  |
|  |  |  |  | Fibre kapok | 77.44 | Fibre poplar down | 0.94 |  |
|  |  |  |  | Fibre turf | 77.41 |  |  |  |
| PS-V | 5a | Fiber | Clear White | Cellulose | 89.31 | Cardboard/cellulose | 0.98 | Semi-Synthetic |
|  |  |  |  | Fibre viscose | 83.28 |  |  |  |
|  |  |  |  | Cellophane | 82.83 | Papercup cellulosic | 0.97 |  |
|  |  |  |  | Fibre viscose dyed | 79.05 |  |  |  |
|  |  |  |  | Fibre grass | 77.29 | Cellulose | 0.92 |  |
|  |  |  |  | Wood mahagoni | 76.47 |  |  |  |
|  |  |  |  | Cellulose | 75.26 | Cellulose | 0.92 |  |
|  |  |  |  | Cellulose wipe | 75.36 |  |  |  |
|  |  |  |  | Wood pine | 74.36 | Hydroxyethyl cellulose | 0.90 |  |
|  |  |  |  | Mask 128S GeATR | 73.17 |  |  |  |
| PS-V | 5b | Fiber | Clear White | Mask 128S GeATR | 97.07 | Cardboard/cellulose | 0.98 | Anthropogenically impacted |
|  |  |  |  | Cellulose acetate filter | 93.71 |  |  |  |
|  |  |  |  | Fibre cotton combers | 91.94 | Papercup cellulosic | 0.97 |  |
|  |  |  |  | Fibre cotton Us pima | 90.86 |  |  |  |
|  |  |  |  | Cellulose | 90.49 | Cellulose | 0.93 |  |
|  |  |  |  | Fibre cotton uzbekistan | 90.47 |  |  |  |
|  |  |  |  | Fibre linen | 90.28 | Cellulose | 0.93 |  |
|  |  |  |  | Fibre hemp fine | 89.53 |  |  |  |
|  |  |  |  | Fibre roasted flax | 89.38 | Cellulose | 0.90 |  |
|  |  |  |  | Fibre hemp rough | 88.01 |  |  |  |
| PS-V | 9a | Fiber | Clear White | Wood pine | 86.76 | Cardboard/cellulose | 0.97 | Anthropogenically impacted |
|  |  |  |  | Wood mahagoni | 84.38 |  |  |  |
|  |  |  |  | Fibre turf | 80.73 | Papercup cellulosic | 0.96 |  |
|  |  |  |  | Fibre poplar down | 76.99 |  |  |  |
|  |  |  |  | Fibre poplar down | 75.80 | Cellulose | 0.91 |  |
|  |  |  |  | Fibre grass | 74.03 |  |  |  |
|  |  |  |  | Fibre kapok | 72.90 | Cellulose | 0.90 |  |
|  |  |  |  | Wood beech | 72.80 |  |  |  |
|  |  |  |  | Fibre cocoanut | 72.34 | Hydroxyethyl cellulose | 0.89 |  |
|  |  |  |  | Mask 128S GeATR | 70.91 |  |  |  |
| PS-V | 9b | Fiber | Clear White | Mask 128S GeATR | 94.20 | Cardboard/cellulose | 0.98 | Anthropogenically impacted |
|  |  |  |  | Cellulose | 89.12 |  |  |  |
|  |  |  |  | Cellulose acetate filter | 85.91 | Papercup cellulosic | 0.97 |  |
|  |  |  |  | Fibre cotton combers | 85.03 |  |  |  |
|  |  |  |  | Cellulose wipe | 84.34 | Cellulose | 0.94 |  |
|  |  |  |  | Fibre hemp fine | 84.31 |  |  |  |
|  |  |  |  | Fibre linen | 84.20 | Cellulose | 0.93 |  |
|  |  |  |  | Fibre cotton Us pima | 83.76 |  |  |  |
|  |  |  |  | Fibre cotton uzbekistan | 83.16 | Hydroxyethyl cellulose | 0.91 |  |
|  |  |  |  | Fibre hemp rough | 82.97 |  |  |  |
| PS-V | 13a | Fiber | Clear White | Cellulose | 86.28 | Cardboard/cellulose | 0.98 | Semi-Synthetic |
|  |  |  |  | Cellophane | 83.33 |  |  |  |
|  |  |  |  | Fibre viscose | 80.71 | Papercup cellulosic | 0.97 |  |
|  |  |  |  | Fibre grass | 78.49 |  |  |  |
|  |  |  |  | Fibre viscose dyed | 78.08 | Cellulose | 0.92 |  |
|  |  |  |  | Wood mahagoni | 76.69 |  |  |  |
|  |  |  |  | Cellulose | 75.13 | Cellulose | 0.92 |  |
|  |  |  |  | Cellulose wipe | 74.55 |  |  |  |
|  |  |  |  | Wood pine | 74.04 | Methyl cellulose | 0.91 |  |
|  |  |  |  | Mask 128S GeATR | 73.12 |  |  |  |
| PS-V | 13b | Fiber | Clear White | Mask 128S GeATR | 96.56 | Cardboard/cellulose | 0.97 | Anthropogenically impacted |
|  |  |  |  | Cellulose acetate filter | 90.71 |  |  |  |
|  |  |  |  | Fibre cotton combers | 90.43 | Papercup cellulosic | 0.97 |  |
|  |  |  |  | Cellulose | 88.67 |  |  |  |
|  |  |  |  | Fibre cotton Us pima | 88.37 | Cellulose | 0.93 |  |
|  |  |  |  | Fibre cotton uzbekistan | 87.63 |  |  |  |
|  |  |  |  | Fibre roasted flax | 86.27 | Cellulose | 0.93 |  |
|  |  |  |  | Fibre hemp fine | 84.11 |  |  |  |
|  |  |  |  | Fibre linen | 83.47 | Cellulose | 0.89 |  |
|  |  |  |  | Fibre hemp rough | 83.42 |  |  |  |
| PS-V | 15a | Fiber | Clear | Cellulose | 83.82 | Cardboard/cellulose | 0.97 | Semi-Synthetic |
|  |  |  |  | Cellophane | 80.76 |  |  |  |
|  |  |  |  | Fibre viscose | 76.02 | Papercup cellulosic | 0.96 |  |
|  |  |  |  | Fibre viscose dyed | 75.30 |  |  |  |
|  |  |  |  | Wood mahagoni | 73.34 | Cellulose | 0.90 |  |
|  |  |  |  | Fibre grass | 73.15 |  |  |  |
|  |  |  |  | Wood pine | 70.33 | Methyl cellulose | 0.90 |  |
|  |  |  |  | Cellulose wipe | 67.94 |  |  |  |
|  |  |  |  | Cellulose | 67.81 | Cellulose | 0.89 |  |
|  |  |  |  | Hydroxyethyl cellulose | 67.63 |  |  |  |
| PS-V | 15b | Fiber | Brown | Polyethylene terephthalate | 95.16 | Polyesterterphthalate | 0.98 | Synthetic |
|  |  |  |  | Polyethylene terephthalate | 94.28 |  |  |  |
|  |  |  |  | Fibre polyester | 93.82 | PET | 0.97 |  |
|  |  |  |  | Polyethylene terephthalate | 93.50 |  |  |  |
|  |  |  |  | Polyester | 93.35 | Polyethylene terephthalate | 0.96 |  |
|  |  |  |  | Polyethylene terephthalate | 92.88 |  |  |  |
|  |  |  |  | Polyethylene terephthalate | 93.61 | Polyethylene terephthalate | 0.96 |  |
|  |  |  |  | Polyethylene terephthalate | 91.96 |  |  |  |
|  |  |  |  | Fibre polyester | 91.96 | Polyester | 0.96 |  |
|  |  |  |  | Polyester | 89.61 |  |  |  |
| PS-V | 20a | Fiber | Clear White | Mask 128S GeATR | 94.66 | Cardboard/cellulose | 0.97 | Anthropogenically impacted |
|  |  |  |  | Fibre cotton combers | 87.98 |  |  |  |
|  |  |  |  | Cellulose | 87.85 | Papercup cellulosic | 0.96 |  |
|  |  |  |  | Cellulose acetate filter | 87.37 |  |  |  |
|  |  |  |  | Fibre cotton Us pima | 85.21 | Cellulose | 0.91 |  |
|  |  |  |  | Fibre cotton uzbekistan | 84.47 |  |  |  |
|  |  |  |  | Fibre roasted flax | 82.60 | Cellulose | 0.91 |  |
|  |  |  |  | Fibre hemp fine | 82.20 |  |  |  |
|  |  |  |  | Fibre hemp rough | 81.87 | Methyl cellulose | 0.89 |  |
|  |  |  |  | Fibre linen | 81.33 |  |  |  |
| PS-V | 20b | Fiber | Clear White | Mask 128S GeATR | 96.76 | Cardboard/cellulose | 0.98 | Anthropogenically impacted |
|  |  |  |  | Cellulose acetate filter | 90.90 |  |  |  |
|  |  |  |  | Cellulose | 90.54 | Papercup cellulosic | 0.97 |  |
|  |  |  |  | Fibre cotton combers | 90.27 |  |  |  |
|  |  |  |  | Fibre cotton Us pima | 88.40 | Cellulose | 0.95 |  |
|  |  |  |  | Fibre cotton uzbekistan | 87.55 |  |  |  |
|  |  |  |  | Fibre roasted flax | 86.29 | Cellulose | 0.94 |  |
|  |  |  |  | Fibre hemp fine | 86.01 |  |  |  |
|  |  |  |  | Fibre linen | 85.15 | Cellulose | 0.91 |  |
|  |  |  |  | Fibre hemp rough | 85.05 |  |  |  |
| PS-V | 27a | Fiber | Clear White | Wood pine | 87.77 | Cardboard/cellulose | 0.96 | Natural |
|  |  |  |  | Wood mahagoni | 83.53 |  |  |  |
|  |  |  |  | Mask 128S GeATR | 79.54 | Papercup cellulosic | 0.96 |  |
|  |  |  |  | Fibre turf | 77.92 |  |  |  |
|  |  |  |  | Cellulose | 77.26 | Cellulose | 0.90 |  |
|  |  |  |  | Fibre linen | 76.43 |  |  |  |
|  |  |  |  | Fibre grass | 76.36 | Cellulose | 0.89 |  |
|  |  |  |  | Cellulose wipe | 75.64 |  |  |  |
|  |  |  |  | Fibre poplar down | 75.25 | Leaf plant | 0.87 |  |
|  |  |  |  | Fibre poplar down | 74.47 |  |  |  |
| PS-V | 27b | Fiber | Clear White | Mask 128S GeATR | 95.14 | Cardboard/cellulose | 0.97 | Anthropogenically impacted |
|  |  |  |  | Cellulose acetate filter | 89.91 |  |  |  |
|  |  |  |  | Cellulose | 89.01 | Papercup cellulosic | 0.96 |  |
|  |  |  |  | Fibre cotton combers | 88.49 |  |  |  |
|  |  |  |  | Fibre cotton Us pima | 87.27 | Cellulose | 0.90 |  |
|  |  |  |  | Fibre cotton uzbekistan | 86.51 |  |  |  |
|  |  |  |  | Fibre hemp fine | 86.38 | Cellulose | 0.90 |  |
|  |  |  |  | Fibre linen | 86.29 |  |  |  |
|  |  |  |  | Fibre roasted flax | 85.90 | Methyl cellulose | 0.88 |  |
|  |  |  |  | Cellulose wipe | 85.33 |  |  |  |
| PS-V | 28a | Fiber | Yellow | Mask 128S GeATR | 96.48 | Cardboard/cellulose | 0.98 | Anthropogenically impacted |
|  |  |  |  | Cellulose | 90.00 |  |  |  |
|  |  |  |  | Cellulose acetate filter | 87.80 | Papercup cellulosic | 0.97 |  |
|  |  |  |  | Fibre cotton combers | 87.76 |  |  |  |
|  |  |  |  | Fibre cotton Us pima | 87.03 | Cellulose | 0.93 |  |
|  |  |  |  | Fibre cotton uzbekistan | 86.65 |  |  |  |
|  |  |  |  | Fibre linen | 85.95 | Cellulose | 0.92 |  |
|  |  |  |  | Cellulose wipe | 85.08 |  |  |  |
|  |  |  |  | Fibre roasted flax | 84.81 | Methyl cellulose | 0.92 |  |
|  |  |  |  | Fibre hemp rough | 84.78 |  |  |  |
| PS-V | 28b | Fiber | Clear White | Mask 128S GeATR | 90.73 | Cardboard/cellulose | 0.98 | Anthropogenically impacted |
|  |  |  |  | Cellulose | 85.16 |  |  |  |
|  |  |  |  | Fibre cotton combers | 83.04 | Papercup cellulosic | 0.97 |  |
|  |  |  |  | Cellulose acetate filter | 82.78 |  |  |  |
|  |  |  |  | Wood pine | 82.43 | Cellulose | 0.93 |  |
|  |  |  |  | Wood mahagoni | 82.27 |  |  |  |
|  |  |  |  | Fibre cotton Us pima | 82.17 | Cellulose | 0.93 |  |
|  |  |  |  | Fibre linen | 81.82 |  |  |  |
|  |  |  |  | Fibre cotton uzbekistan | 81.48 | Hydroxyethyl cellulose | 0.90 |  |
|  |  |  |  | Cellulose wipe | 81.30 |  |  |  |
| PS-V | 29a | Fiber | Clear | Mask 128S GeATR | 93.36 | Cardboard/cellulose | 0.97 | Anthropogenically impacted |
|  |  |  |  | Cellulose acetate filter | 89.23 |  |  |  |
|  |  |  |  | Cellulose | 88.89 | Papercup cellulosic | 0.96 |  |
|  |  |  |  | Fibre cotton combers | 88.14 |  |  |  |
|  |  |  |  | Fibre cotton Us pima | 87.05 | Cellulose | 0.92 |  |
|  |  |  |  | Fibre cotton uzbekistan | 86.52 |  |  |  |
|  |  |  |  | Cellulose wipe | 85.67 | Cellulose | 0.91 |  |
|  |  |  |  | Fibre roasted flax | 85.59 |  |  |  |
|  |  |  |  | Fibre linen | 85.35 | Methyl cellulose | 0.90 |  |
|  |  |  |  | Fibre hemp fine | 85.12 |  |  |  |
| PS-V | C1a | Fiber | White | Mask 128S GeATR | 86.79 | Cardboard/cellulose | 0.98 | Anthropogenically impacted |
|  |  |  |  | Wood pine | 84.78 |  |  |  |
|  |  |  |  | Wood mahagoni | 83.61 | Papercup cellulosic | 0.97 |  |
|  |  |  |  | Cellulose | 83.08 |  |  |  |
|  |  |  |  | Fibre cotton combers | 79.94 | Cellulose | 0.94 |  |
|  |  |  |  | Cellulose acetate filter | 79.88 |  |  |  |
|  |  |  |  | Cellulose wipe | 79.21 | Cellulose | 0.93 |  |
|  |  |  |  | Fibre cotton Us pima | 78.83 |  |  |  |
|  |  |  |  | Fibre hemp fine | 78.71 | Hydroxyethyl cellulose | 0.90 |  |
|  |  |  |  | Fibre linen | 78.51 |  |  |  |
| PS-V | C1b | Fiber Bundle | White | Mask 128S GeATR | 85.50 | Cardboard/cellulose | 0.97 | Anthropogenically impacted |
|  |  |  |  | Wood pine | 84.08 |  |  |  |
|  |  |  |  | Wood mahagoni | 83.74 | Papercup cellulosic | 0.97 |  |
|  |  |  |  | Cellulose | 82.82 |  |  |  |
|  |  |  |  | Cellulose wipe | 79.57 | Cellulose | 0.91 |  |
|  |  |  |  | Fibre grass | 79.31 |  |  |  |
|  |  |  |  | Fibre linen | 77.94 | Cellulose | 0.90 |  |
|  |  |  |  | Cellophane | 77.48 |  |  |  |
|  |  |  |  | Fibre hemp fine | 76.78 | Methyl cellulose | 0.90 |  |
|  |  |  |  | Fibre poplar down | 73.49 |  |  |  |
| PS-V | C3a | Fiber | Clear | Cellulose | 84.63 | Cardboard/cellulose | 0.97 | Semi-Synthetic |
|  |  |  |  | Fibre viscose | 78.75 |  |  |  |
|  |  |  |  | Cellophane | 76.39 | Papercup cellulosic | 0.96 |  |
|  |  |  |  | Fibre viscose dyed | 75.72 |  |  |  |
|  |  |  |  | Wood mahagoni | 73.64 | Cellulose | 0.90 |  |
|  |  |  |  | Fibre grass | 73.63 |  |  |  |
|  |  |  |  | Wood pine | 71.65 | Cellulose | 0.90 |  |
|  |  |  |  | Cellulose wipe | 70.46 |  |  |  |
|  |  |  |  | Cellulose | 69.39 | Fibre viscose dyed | 0.89 |  |
|  |  |  |  | Fibre urtica dioica L conar fibra | 68.32 |  |  |  |
| PS-V | C3b | Fragment | Clear | Polyethylene low density | 99.04 | Polyethylene | 0.97 | Synthetic |
|  |  |  |  | Polyethylene low density | 98.82 |  |  |  |
|  |  |  |  | Polyethylene low density | 98.72 | Polyethylene | 0.96 |  |
|  |  |  |  | Polyethylene low density | 98.57 |  |  |  |
|  |  |  |  | Fibre thermoplastic elastomere | 98.43 | Fibre thermoplastic elastomere | 0.95 |  |
|  |  |  |  | Polyethylene low density | 98.34 |  |  |  |
|  |  |  |  | Polyethylene low density | 98.15 | Polyethylene low density | 0.95 |  |
|  |  |  |  | Polyethylene low density | 97.49 |  |  |  |
|  |  |  |  | Polyethylene low density | 97.45 | Polyethylene low density linear | 0.95 |  |
|  |  |  |  | Polyethyelen foamed | 97.43 |  |  |  |
| PS-S | 1a | Fiber | Brown | Wood pine | 86.76 | Cardboard/cellulose | 0.97 | Natural |
|  |  |  |  | Wood mahagoni | 83.39 |  |  |  |
|  |  |  |  | Mask 128S GeATR | 81.67 | Papercup cellulosic | 0.97 |  |
|  |  |  |  | Cellulose | 79.29 |  |  |  |
|  |  |  |  | Fibre turf | 77.22 | Cellulose | 0.91 |  |
|  |  |  |  | Fibre linen | 77.13 |  |  |  |
|  |  |  |  | Fibre poplar down | 76.98 | Cellulose | 0.90 |  |
|  |  |  |  | Cellulose wipe | 76.37 |  |  |  |
|  |  |  |  | Fibre cotton combers | 76.34 | Methyl cellulose | 0.89 |  |
|  |  |  |  | Fibre poplar down | 76.10 |  |  |  |
| PS-S | 2a | Fiber | Clear White | Mask 128S GeATR | 92.83 | Cardboard/cellulose | .97\ | Anthropogenically impacted |
|  |  |  |  | Fibre cotton combers | 87.82 |  |  |  |
|  |  |  |  | Cellulose acetate filter | 87.63 | Papercup cellulosic | 0.96 |  |
|  |  |  |  | Fibre cotton Us pima | 85.34 |  |  |  |
|  |  |  |  | Cellulose | 84.92 | Cellulose | 0.94 |  |
|  |  |  |  | Fibre cotton uzbekistan | 84.81 |  |  |  |
|  |  |  |  | Fibre roasted flax | 83.06 | Cellulose | 0.94 |  |
|  |  |  |  | Fibre hemp fine | 81.65 |  |  |  |
|  |  |  |  | Fibre linen | 80.80 | Hydroxyethyl cellulose | 0.90 |  |
|  |  |  |  | Fibre hemp rough | 80.46 |  |  |  |
| PS-S | 7a | Fiber | Clear White | Mask 128S GeATR | 96.43 | Cardboard/cellulose | 0.97 | Anthropogenically impacted |
|  |  |  |  | Cellulose acetate filter | 90.26 |  |  |  |
|  |  |  |  | Fibre cotton combers | 89.94 | Papercup cellulosic | 0.97 |  |
|  |  |  |  | Cellulose | 88.05 |  |  |  |
|  |  |  |  | Fibre cotton Us pima | 87.86 | Cellulose | 0.94 |  |
|  |  |  |  | Fibre cotton uzbekistan | 87.04 |  |  |  |
|  |  |  |  | Fibre roasted flax | 85.73 | Cellulose | 0.94 |  |
|  |  |  |  | Fibre hemp fine | 83.98 |  |  |  |
|  |  |  |  | Fibre hemp rough | 83.25 | Cellulose | 0.90 |  |
|  |  |  |  | Fibre linen | 83.18 |  |  |  |
| PS-S | 11a | Fragment | Clear | Polyethylene low density | 99.11 | Polyethylene | 0.96 | Synthetic |
|  |  |  |  | Polyethylene low density | 98.84 |  |  |  |
|  |  |  |  | Polyethylene low density | 98.66 | Polyethylene | 0.96 |  |
|  |  |  |  | Polyethylene low density | 98.45 |  |  |  |
|  |  |  |  | Fibre thermoplastic elastomere | 98.40 | Fibre thermoplastic elastomere | 0.95 |  |
|  |  |  |  | Polyethylene low density | 98.28 |  |  |  |
|  |  |  |  | Polyethylene low density | 98.11 | Polyethylene low density | 0.95 |  |
|  |  |  |  | Polyethylene low density | 97.24 |  |  |  |
|  |  |  |  | Polyethylene low density | 97.15 | polyethylene low density linear | 0.95 |  |
|  |  |  |  | Polyethylene low density | 97.06 |  |  |  |
| PS-S | 11b | Fiber | Purple | Mask 128S GeATR | 96.55 | Cardboard/cellulose | 0.97 | Anthropogenically impacted |
|  |  |  |  | Cellulose acetate filter | 88.42 |  |  |  |
|  |  |  |  | Fibre cotton combers | 88.05 | Papercup cellulosic | 0.97 |  |
|  |  |  |  | Fibre cotton Us pima | 86.81 |  |  |  |
|  |  |  |  | Cellulose | 86.14 | Cellulose | 0.94 |  |
|  |  |  |  | Fibre cotton uzbekistan | 85.88 |  |  |  |
|  |  |  |  | Fibre roasted flax | 84.72 | Cellulose | 0.93 |  |
|  |  |  |  | Fibre linen | 83.98 |  |  |  |
|  |  |  |  | Fibre hemp fine | 82.82 | Hydroxyethyl cellulose | 0.90 |  |
|  |  |  |  | Fibre hemp rough | 82.66 |  |  |  |
| PS-S | 14a | Fiber | Clear | Mask 128S GeATR | 94.89 | Cardboard/cellulose | 0.98 | Anthropogenically impacted |
|  |  |  |  | Cellulose acetate filter | 89.62 |  |  |  |
|  |  |  |  | Cellulose | 89.53 | Papercup cellulosic | 0.97 |  |
|  |  |  |  | Fibre cotton combers | 88.44 |  |  |  |
|  |  |  |  | Fibre cotton Us pima | 86.22 | Cellulose | 0.95 |  |
|  |  |  |  | Fibre hemp fine | 85.53 |  |  |  |
|  |  |  |  | Fibre cotton uzbekistan | 85.17 | Cellulose | 0.95 |  |
|  |  |  |  | Fibre roasted flax | 84.24 |  |  |  |
|  |  |  |  | Fibre linen | 83.79 | Hydroxyethyl cellulose | 0.91 |  |
|  |  |  |  | Fibre hemp rough | 83.51 |  |  |  |
| PS-S | 14b | Fiber | White | Mask 128S GeATR | 96.35 | Cardboard/cellulose | 0.97 | Anthropogenically impacted |
|  |  |  |  | Cellulose | 89.54 |  |  |  |
|  |  |  |  | Fibre cotton combers | 89.51 | Papercup cellulosic | 0.97 |  |
|  |  |  |  | Cellulose acetate filter | 88.90 |  |  |  |
|  |  |  |  | Fibre cotton Us pima | 87.05 | Cellulose | 0.93 |  |
|  |  |  |  | Fibre cotton uzbekistan | 86.54 |  |  |  |
|  |  |  |  | Fibre roasted flax | 84.91 | Cellulose | 0.93 |  |
|  |  |  |  | Fibre hemp fine | 84.34 |  |  |  |
|  |  |  |  | Fibre linen | 84.21 | Hydroxyethyl cellulose | 0.90 |  |
|  |  |  |  | Fibre hemp rough | 84.05 |  |  |  |
| PS-S | 18a | Fiber | Blue | Mask 128S GeATR | 89.63 | Cardboard/cellulose | 0.98 | Anthropogenically impacted |
|  |  |  |  | Cellulose | 86.88 |  |  |  |
|  |  |  |  | Cellulose wipe | 82.88 | Papercup cellulosic | 0.97 |  |
|  |  |  |  | Fibre hemp fine | 82.88 |  |  |  |
|  |  |  |  | Cellulose acetate filter | 82.80 | Cellulose | 0.91 |  |
|  |  |  |  | Fibre linen | 82.71 |  |  |  |
|  |  |  |  | Fibre cotton combers | 81.47 | Cellulose | 0.91 |  |
|  |  |  |  | Fibre hemp rough | 80.90 |  |  |  |
|  |  |  |  | Fibre grass | 80.57 | Methyl cellulose | 0.90 |  |
|  |  |  |  | Fibre cotton Us pima | 80.56 |  |  |  |
| PS-S | 18b | Fiber | Clear White | Wood mahagoni | 83.71 | Cardboard/cellulose | 0.97 | Natural |
|  |  |  |  | Bentonite (brown) | 79.36 |  |  |  |
|  |  |  |  | Wood pine | 79.19 | Papercup cellulosic | 0.96 |  |
|  |  |  |  | Wood beech | 77.73 |  |  |  |
|  |  |  |  | Fibre poplar down | 77.45 | Cellulose | 0.90 |  |
|  |  |  |  | Fibre turf | 77.14 |  |  |  |
|  |  |  |  | Cellophane | 77.00 | Methyl cellulose | 0.89 |  |
|  |  |  |  | Fibre grass | 76.33 |  |  |  |
|  |  |  |  | Fibre poplar down | 75.97 | Cellulose | 0.89 |  |
|  |  |  |  | Cellulose | 75.74 |  |  |  |
| PS-S | 21a | Fiber | Clear | Cellulose | 96.08 | Cardboard/cellulose | 0.98 | Semi-Synthetic |
|  |  |  |  | Cellophane | 84.04 |  |  |  |
|  |  |  |  | Fibre viscose | 82.12 | Papercup cellulosic | 0.98 |  |
|  |  |  |  | Fibre grass | 79.30 |  |  |  |
|  |  |  |  | Wood mahagoni | 79.10 | Cellulose | 0.94 |  |
|  |  |  |  | Cellulose | 78.76 |  |  |  |
|  |  |  |  | Fibre viscose dyed | 78.25 | Cellulose | 0.93 |  |
|  |  |  |  | Cellulose wipe | 77.98 |  |  |  |
|  |  |  |  | Mask 128S GeATR | 76.89 | Hydroxyethyl cellulose | 0.91 |  |
|  |  |  |  | Wood pine | 76.36 |  |  |  |
| PS-S | 21b | Fiber | Clear White | Mask 128S GeATR | 95.50 | Cardboard/cellulose | 0.98 | Anthropogenically impacted |
|  |  |  |  | Cellulose acetate filter | 89.62 |  |  |  |
|  |  |  |  | Fibre cotton combers | 89.12 | Papercup cellulosic | 0.97 |  |
|  |  |  |  | Cellulose | 88.70 |  |  |  |
|  |  |  |  | Fibre cotton Us pima | 86.49 | Cellulose | 0.93 |  |
|  |  |  |  | Fibre cotton uzbekistan | 85.34 |  |  |  |
|  |  |  |  | Fibre hemp fine | 84.59 | Cellulose | 0.93 |  |
|  |  |  |  | Fibre roasted flax | 84.44 |  |  |  |
|  |  |  |  | Fibre linen | 83.15 | Hydroxyethyl cellulose | 0.99 |  |
|  |  |  |  | Fibre hemp rough | 83.02 |  |  |  |
| PS-S | 25a | Fiber | Blue | Mask 128S GeATR | 83.39 | Cardboard/cellulose | 0.98 | Anthropogenically impacted |
|  |  |  |  | Cellulose | 82.38 |  |  |  |
|  |  |  |  | Cellulose | 81.18 | Papercup cellulosic | 0.97 |  |
|  |  |  |  | Fibre viscose | 79.82 |  |  |  |
|  |  |  |  | Cellulose acetate filter | 79.13 | Cellulose | 0.94 |  |
|  |  |  |  | Fibre hemp fine | 78.96 |  |  |  |
|  |  |  |  | Cellulose wipe | 78.68 | Cellulose | 0.93 |  |
|  |  |  |  | Fibre linen | 78.07 |  |  |  |
|  |  |  |  | Fibre cotton combers | 77.94 | Cellulose | 0.90 |  |
|  |  |  |  | Fibre hemp rough | 76.62 |  |  |  |
| PS-S | 25b | Fiber | Clear | Mask 128S GeATR | 98.00 | Cardboard/cellulose | 0.98 | Anthropogenically impacted |
|  |  |  |  | Cellulose acetate filter | 93.19 |  |  |  |
|  |  |  |  | Fibre cotton combers | 92.05 | Papercup cellulosic | 0.97 |  |
|  |  |  |  | Fibre cotton Us pima | 90.96 |  |  |  |
|  |  |  |  | Fibre cotton uzbekistan | 90.28 | Cellulose | 0.94 |  |
|  |  |  |  | Cellulose | 90.25 |  |  |  |
|  |  |  |  | Fibre linen | 89.55 | Cellulose | 0.93 |  |
|  |  |  |  | Fibre roasted flax | 89.17 |  |  |  |
|  |  |  |  | Fibre hemp fine | 88.36 | Hydroxyethyl cellulose | 0.90 |  |
|  |  |  |  | Fibre hemp rough | 87.57 |  |  |  |
| PS-S | 28a | Fiber | Clear | Polyethylene terephthalate | 95.91 | Polyesterterphthalate | 0.95 | Synthetic |
|  |  |  |  | Polyester | 94.86 |  |  |  |
|  |  |  |  | Polyethylene terephthalate | 94.67 | PET | 0.95 |  |
|  |  |  |  | Polyethylene terephthalate | 94.42 |  |  |  |
|  |  |  |  | Polyethylene terephthalate | 94.13 | Polyester | 0.95 |  |
|  |  |  |  | Polyethylene terephthalate | 91.30 |  |  |  |
|  |  |  |  | Polyethylene terephthalate | 90.28 | Polyethylene terephthalate | 0.94 |  |
|  |  |  |  | Fibre polyester | 90.19 |  |  |  |
|  |  |  |  | Fibre polyester | 87.90 | Polyethylene terephthalate | 0.94 |  |
|  |  |  |  | Polyethylene terephthalate | 86.41 |  |  |  |
| PS-S | 28b | Fiber | White | Wood pine | 87.10 | Cardboard/cellulose | 0.96 | Natural |
|  |  |  |  | Wood mahagoni | 86.18 |  |  |  |
|  |  |  |  | Fibre turf | 81.47 | Papercup cellulosic | 0.96 |  |
|  |  |  |  | Fibre poplar down | 79.11 |  |  |  |
|  |  |  |  | Fibre poplar down | 78.03 | Cellulose | 0.90 |  |
|  |  |  |  | Fibre grass | 76.49 |  |  |  |
|  |  |  |  | Fibre cocoanut | 75.86 | Cellulose | 0.89 |  |
|  |  |  |  | Fibre kapok | 75.19 |  |  |  |
|  |  |  |  | Wood beech | 75.03 | Fibre poplar down | 0.87 |  |
|  |  |  |  | Mask 128S GeATR | 71.76 |  |  |  |
| PS-S | C1a | Fiber | Clear White | Fibre poplar down | .87.26 | Cardboard/cellulose | 0.97 | Natural |
|  |  |  |  | Mask 128S GeATR | 86.53 |  |  |  |
|  |  |  |  | Fibre poplar down | 86.20 | Papercup cellulosic | 0.96 |  |
|  |  |  |  | Wood mahagoni | 86.14 |  |  |  |
|  |  |  |  | Fibre kapok | 84.10 | Cellulose | 0.92 |  |
|  |  |  |  | Wood beech | 81.50 |  |  |  |
|  |  |  |  | Cellulose | 80.60 | Cellulose | 0.91 |  |
|  |  |  |  | Cellulose wipe | 79.75 |  |  |  |
|  |  |  |  | Fibre linen | 79.09 | Methyl cellulose | 0.90 |  |
|  |  |  |  | Fibre grass | 78.34 |  |  |  |
| PS-S | C1b | Fiber | Clear White | Mask 128S GeATR | 96.49 | Cardboard/cellulose | 0.98 | Anthropogenically impacted |
|  |  |  |  | Cellulose acetate filter | 90.05 |  |  |  |
|  |  |  |  | Cellulose | 88.92 | Papercup cellulosic | 0.97 |  |
|  |  |  |  | Fibre cotton combers | 88.21 |  |  |  |
|  |  |  |  | Fibre cotton Us pima | 87.46 | Cellulose | 0.93 |  |
|  |  |  |  | Fibre cotton uzbekistan | 86.65 |  |  |  |
|  |  |  |  | Fibre roasted flax | 85.83 | Cellulose | 0.93 |  |
|  |  |  |  | Fibre hemp fine | 84.53 |  |  |  |
|  |  |  |  | Fibre linen | 84.33 | Cellulose | 0.90 |  |
|  |  |  |  | Fibre hemp rough | 83.41 |  |  |  |
| PS-S | C2a | fiber | Blue | Cellophane | 79.15 | Cardboard/cellulose | 0.98 | Semi-Synthetic |
|  |  |  |  | Cellulose | 67.81 |  |  |  |
|  |  |  |  | Fibre viscose | 62.92 | Papercup cellulosic | 0.97 |  |
|  |  |  |  | Fibre viscose dyed | 61.05 |  |  |  |
|  |  |  |  | Fibre grass | 61.01 | Cellulose | 0.92 |  |
|  |  |  |  | Cellophane | 57.27 |  |  |  |
|  |  |  |  | Wood mahagoni | 56.48 | Cellulose | 0.91 |  |
|  |  |  |  | Cellulose | 55.00 |  |  |  |
|  |  |  |  | Hydroxyethyl cellulose | 54.98 | Methyl cellulose | 0.91 |  |
|  |  |  |  | Fibre turf | 53.91 |  |  |  |
| PS-S | C2b | Fragment | Clear White | Polypropylene | 98.59 | Polypropylene | 0.96 | Synthetic |
|  |  |  |  | Fibre polypropylene | 98.47 |  |  |  |
|  |  |  |  | Polypropylene | 98.15 | Polypropylene | 0.96 |  |
|  |  |  |  | Polypropylene | 98.09 |  |  |  |
|  |  |  |  | Polypropylene | 97.84 | Polypropylene | 0.95 |  |
|  |  |  |  | Polypropylene | 97.68 |  |  |  |
|  |  |  |  | Fibre polypropylene dyed | 97.40 | Polypropylene | 0.95 |  |
|  |  |  |  | Polypropylene | 97.02 |  |  |  |
|  |  |  |  | KOH lid polypropylene | 96.29 | Polypropylene | 0.95 |  |
|  |  |  |  | Polypropylene | 95.45 |  |  |  |
| PS-S | A4b | Fiber | Blue | Mask 128S GeATR | 96.39 | Cardboard/cellulose | 0.97 | Anthropogenically impacted |
|  |  |  |  | Cellulose acetate filter | 88.28 |  |  |  |
|  |  |  |  | Fibre cotton combers | 88.05 | Papercup cellulosic | 0.96 |  |
|  |  |  |  | Cellulose | 87.91 |  |  |  |
|  |  |  |  | Fibre cotton Us pima | 86.50 | Cellulose | 0.92 |  |
|  |  |  |  | Fibre cotton uzbekistan | 85.78 |  |  |  |
|  |  |  |  | Fibre roasted flax | 84.73 | Cellulose | 0.91 |  |
|  |  |  |  | Fibre hemp fine | 82.78 |  |  |  |
|  |  |  |  | Fibre linen | 82.65 | Methyl cellulose | 0.90 |  |
|  |  |  |  | Fibre hemp rough | 82.03 |  |  |  |
| PS-S | A5a | Fiber | Clear | Cellophane | 81.73 | Cardboard/cellulose | 0.98 | Anthropogenically impacted |
|  |  |  |  | Mask 128S GeATR | 81.60 |  |  |  |
|  |  |  |  | Cellulose | 79.90 | Papercup cellulosic | 0.97 |  |
|  |  |  |  | Cellulose | 77.45 |  |  |  |
|  |  |  |  | Cellulose wipe | 75.78 | Cellulose | 0.95 |  |
|  |  |  |  | Fibre viscose | 74.42 |  |  |  |
|  |  |  |  | Fibre linen | 74.39 | Cellulose | 0.94 |  |
|  |  |  |  | Fibre hemp fine | 73.98 |  |  |  |
|  |  |  |  | Wood mahagoni | 73.97 | Hydroxyethyl cellulose | 0.92 |  |
|  |  |  |  | Fibre grass | 73.68 |  |  |  |
| PS-S | A5b | Fiber | Brown | Cellulose | 88.46 | Cardboard/cellulose | 0.97 | Semi-Synthetic |
|  |  |  |  | Cellophane | 82.96 |  |  |  |
|  |  |  |  | Fibre viscose | 82.38 | Papercup cellulosic | 0.97 |  |
|  |  |  |  | Fibre viscose dyed | 79.82 |  |  |  |
|  |  |  |  | Fibre grass | 75.85 | Cellulose | 0.92 |  |
|  |  |  |  | Wood mahagoni | 74.11 |  |  |  |
|  |  |  |  | Wood pine | 72.48 | Cellulose | 0.92 |  |
|  |  |  |  | Cellulose | 71.85 |  |  |  |
|  |  |  |  | Cellulose wipe | 71.41 | Hydroxyethyl cellulose | 0.90 |  |
|  |  |  |  | Fibre turf | 68.66 |  |  |  |
| BR-S | 3a | Fiber | White | Mask 128S GeATR | 96.22 | Cardboard/cellulose | 0.97 | Anthropogenically impacted |
|  |  |  |  | Cellulose acetate filter | 88.36 |  |  |  |
|  |  |  |  | Fibre cotton combers | 87.40 | Papercup cellulosic | 0.96 |  |
|  |  |  |  | Cellulose | 87.01 |  |  |  |
|  |  |  |  | Fibre cotton Us pima | 86.47 | Cellulose | 0.94 |  |
|  |  |  |  | Fibre cotton uzbekistan | 86.05 |  |  |  |
|  |  |  |  | Fibre roasted flax | 84.67 | Cellulose | 0.94 |  |
|  |  |  |  | Fibre linen | 82.67 |  |  |  |
|  |  |  |  | Fibre hemp fine | 82.49 | Cellulose | 0.90 |  |
|  |  |  |  | Fibre hemp rough | 82.06 |  |  |  |
| BR-S | 3b | Fiber | Brown | Mask 128S GeATR | 94.96 | Cardboard/cellulose | 0.96 | Anthropogenically impacted |
|  |  |  |  | Cellulose | 90.41 |  |  |  |
|  |  |  |  | Cellulose wipe | 89.64 | Papercup cellulosic | 0.95 |  |
|  |  |  |  | Cellulose acetate filter | 89.63 |  |  |  |
|  |  |  |  | Fibre cotton combers | 88.36 | Cellulose | 0.90 |  |
|  |  |  |  | Fibre cotton Us pima | 87.51 |  |  |  |
|  |  |  |  | Fibre cotton uzbekistan | 87.45 | Cellulose | 0.89 |  |
|  |  |  |  | Fibre linen | 87.04 |  |  |  |
|  |  |  |  | Fibre hemp fine | 86.34 | Methyl cellulose | 0.87 |  |
|  |  |  |  | Fibre hemp rough | 85.98 |  |  |  |
| BR-S | 8b | Fiber | Black | Mask 128S GeATR | 95.15 | Cardboard/cellulose | 0.96 | Anthropogenically impacted |
|  |  |  |  | Fibre cotton combers | 86.50 |  |  |  |
|  |  |  |  | Cellulose | 86.46 | Papercup cellulosic | 0.95 |  |
|  |  |  |  | Cellulose acetate filter | 86.35 |  |  |  |
|  |  |  |  | Fibre cotton Us pima | 84.86 | Cellulose | 0.90 |  |
|  |  |  |  | Fibre cotton uzbekistan | 84.04 |  |  |  |
|  |  |  |  | Fibre roasted flax | 82.44 | Cellulose | 0.89 |  |
|  |  |  |  | Fibre linen | 81.17 |  |  |  |
|  |  |  |  | Fibre hemp fine | 81.10 | Methyl cellulose | 0.87 |  |
|  |  |  |  | Fibre hemp rough | 80.67 |  |  |  |
| BR-S | 9b | Fiber | Yellow | Mask 128S GeATR | 92.21 | Cardboard/cellulose | 0.96 | Anthropogenically impacted |
|  |  |  |  | Cellulose | 85.88 |  |  |  |
|  |  |  |  | Fibre cotton combers | 83.12 | Papercup cellulosic | 0.96 |  |
|  |  |  |  | Cellulose acetate filter | 82.36 |  |  |  |
|  |  |  |  | Fibre cotton Us pima | 82.01 | Cellulose | 0.90 |  |
|  |  |  |  | Fibre cotton uzbekistan | 81.53 |  |  |  |
|  |  |  |  | Cellulose wipe | 80.58 | Cellulose | 0.89 |  |
|  |  |  |  | Fibre linen | 79.78 |  |  |  |
|  |  |  |  | Fibre hemp rough | 79.38 | Methyl cellulose | 0.87 |  |
|  |  |  |  | Fibre roasted flax | 79.25 |  |  |  |
| BR-S | 12a | Fiber | Black | Polyethylene terephthalate | 91.10 | Polyesterterphthalate | 0.97 | Synthetic |
|  |  |  |  | Polyester | 90.00 |  |  |  |
|  |  |  |  | Polyethylene terephthalate | 89.12 | polyester | 0.97 |  |
|  |  |  |  | Polyethylene terephthalate | 88.60 |  |  |  |
|  |  |  |  | Polyethylene terephthalate | 88.59 | polyester | 0.97 |  |
|  |  |  |  | Polyethylene terephthalate | 88.40 |  |  |  |
|  |  |  |  | Polyethylene terephthalate | 87.64 | PET | 0.97 |  |
|  |  |  |  | Fibre polyester | 86.88 |  |  |  |
|  |  |  |  | Epoxide resin | 84.66 | Polyethylene terephthalate | 0.96 |  |
|  |  |  |  | Fibre polyester | 84.49 |  |  |  |
| BR-S | 12b | Fiber | Brown | Polyethylene terephthalate | 90.84 | Polyesterterphthalate | 0.97 | Synthetic |
|  |  |  |  | Polyethylene terephthalate | 89.94 |  |  |  |
|  |  |  |  | Polyester | 89.65 | PET | 0.95 |  |
|  |  |  |  | Polyethylene terephthalate | 88.26 |  |  |  |
|  |  |  |  | Polyester epoxide | 88.05 | Polyester | 0.95 |  |
|  |  |  |  | Fibre polyester | 88.03 |  |  |  |
|  |  |  |  | Epoxide resin | 87.89 | Polyethylene terephthalate | 0.94 |  |
|  |  |  |  | Polyethylene terephthalate | 87.55 |  |  |  |
|  |  |  |  | Polyethylene terephthalate | 86.57 | Amorphous polyethylene terephthalate | 0.94 |  |
|  |  |  |  | Fibre polyester | 86.36 |  |  |  |
| BR-S | 13a | Fiber | Black | Polyethylene terephthalate | 95.24 | Polyesterterphthalate | 0.97 | Synthetic |
|  |  |  |  | Polyester | 94.24 |  |  |  |
|  |  |  |  | Polyethylene terephthalate | 93.84 | PET | 0.96 |  |
|  |  |  |  | Polyethylene terephthalate | 93.36 |  |  |  |
|  |  |  |  | Polyethylene terephthalate | 92.77 | Polyethylene terephthalate | 0.95 |  |
|  |  |  |  | Polyethylene terephthalate | 91.52 |  |  |  |
|  |  |  |  | Polyethylene terephthalate | 91.02 | Polyester | 0.95 |  |
|  |  |  |  | Fibre polyester | 90.64 |  |  |  |
|  |  |  |  | Fibre polyester | 88.93 | Polyethylene terephthalate | 0.95 |  |
|  |  |  |  | Polyethylene terephthalate | 87.08 |  |  |  |
| BR-S | 13b | Fiber | Clear | Cellulose | 81.16 | Cardboard/cellulose | 0.98 | Semi-Synthetic |
|  |  |  |  | Cellophane | 80.31 |  |  |  |
|  |  |  |  | Mask 128S GeATR | 77.81 | Papercup cellulosic | 0.97 |  |
|  |  |  |  | Cellulose | 77.57 |  |  |  |
|  |  |  |  | Fibre viscose | 77.24 | Cellulose | 0.92 |  |
|  |  |  |  | Fibre grass | 74.20 |  |  |  |
|  |  |  |  | Cellulose wipe | 74.06 | Cellulose | 0.91 |  |
|  |  |  |  | Wood mahagoni | 73.88 |  |  |  |
|  |  |  |  | Fibre viscose dyed | 72.76 | Methyl cellulose | 91.00 |  |
|  |  |  |  | Fibre hemp fine | 72.48 |  |  |  |
| BR-S | 15a | Fiber | Clear | Polyethylene terephthalate | 97.09 | Polyesterterphthalate | 0.98 | Synthetic |
|  |  |  |  | Polyethylene terephthalate | 95.90 |  |  |  |
|  |  |  |  | Polyester | 95.58 | Polyethylene terephthalate | 0.98 |  |
|  |  |  |  | Polyethylene terephthalate | 95.54 |  |  |  |
|  |  |  |  | Polyethylene terephthalate | 95.18 | PET | 0.98 |  |
|  |  |  |  | Fibre polyester | 93.63 |  |  |  |
|  |  |  |  | Polyethylene terephthalate | 93.00 | Polyethylene terephthalate | 0.97 |  |
|  |  |  |  | Polyethylene terephthalate | 92.80 |  |  |  |
|  |  |  |  | Fibre polyester | 92.51 | Polyethylene terephthalate | 0.97 |  |
|  |  |  |  | Polyester | 90.31 |  |  |  |
| BR-S | 15b | Fiber | Black | Polyethylene terephthalate | 92.22 | Polyesterterphthalate | 0.98 | Synthetic |
|  |  |  |  | Polyethylene terephthalate | 91.25 |  |  |  |
|  |  |  |  | Polyester | 90.26 | Polyester | 0.97 |  |
|  |  |  |  | Fibre Polyester | 89.56 |  |  |  |
|  |  |  |  | Polyethylene terephthalate | 89.35 | PET | 0.97 |  |
|  |  |  |  | Polyethylene terephthalate | 88.23 |  |  |  |
|  |  |  |  | Polyethylene terephthalate | 88.13 | Polyester | 0.96 |  |
|  |  |  |  | Epoxide resin | 87.73 |  |  |  |
|  |  |  |  | Polyethylene terephthalate | 87.41 | Polyethylene terephthalate | 0.96 |  |
|  |  |  |  | Polyethylene terephthalate | 86.91 |  |  |  |
| BR-S | 20a | Fiber | Clear | Polyethylene terephthalate | 94.89 | Polyesterterphthalate | 0.97 | Synthetic |
|  |  |  |  | Polyethylene terephthalate | 93.88 |  |  |  |
|  |  |  |  | Polyethylene terephthalate | 92.95 | Polyester | 0.95 |  |
|  |  |  |  | Polyethylene terephthalate | 92.65 |  |  |  |
|  |  |  |  | Polyester | 92.64 | PET | 0.95 |  |
|  |  |  |  | Polyethylene terephthalate | 92.29 |  |  |  |
|  |  |  |  | Polyethylene terephthalate | 91.86 | Polyethylene terephthalate | 0.95 |  |
|  |  |  |  | Fibre polyester | 91.73 |  |  |  |
|  |  |  |  | Fibre polyester | 80.28 | Polyethylene terephthalate | 0.95 |  |
|  |  |  |  | Polyethylene terephthalate | 88.61 |  |  |  |
| BR-S | 20b | Fiber | Black | Mask 128S GeATR | 95.00 | Cardboard/cellulose | 0.97 | Anthropogenically impacted |
|  |  |  |  | Cellulose | 91.00 |  |  |  |
|  |  |  |  | Cellulose wipe | 89.53 | Papercup cellulosic | 0.96 |  |
|  |  |  |  | Cellulose acetate filter | 87.59 |  |  |  |
|  |  |  |  | Fibre linen | 86.00 | Cellulose | 0.92 |  |
|  |  |  |  | Fibre cotton combers | 86.42 |  |  |  |
|  |  |  |  | Fibre cotton uzbekistan | 86.13 | Cellulose | 0.92 |  |
|  |  |  |  | Fibre cotton Us pima | 85.98 |  |  |  |
|  |  |  |  | Fibre hemp fine | 85.73 | Hydroxyethyl cellulose | 0.88 |  |
|  |  |  |  | Fibre hemp rough | 75.47 |  |  |  |
| BR-S | 26a | Fiber | White | Mask 128S GeATR | 95.00 | Cardboard/cellulose | 0.98 | Anthropogenically impacted |
|  |  |  |  | Cellulose | 91.00 |  |  |  |
|  |  |  |  | Cellulose wipe | 89.53 | Papercup cellulosic | 0.97 |  |
|  |  |  |  | Cellulose acetate filter | 87.59 |  |  |  |
|  |  |  |  | Fibre linen | 86.60 | Cellulose | 0.93 |  |
|  |  |  |  | Fibre cotton combers | 86.42 |  |  |  |
|  |  |  |  | Fibre cotton uzbekistan | 86.13 | Cellulose | 0.92 |  |
|  |  |  |  | Fibre cotton Us pima | 85.98 |  |  |  |
|  |  |  |  | Fibre hemp fine | 85.73 | Methyl cellulose | 0.91 |  |
|  |  |  |  | Fibre hemp rough | 85.47 |  |  |  |
| BR-S | 26b | Fiber | Blue | Mask 128S GeATR | 95.67 | Cardboard/cellulose | 0.97 | Anthropogenically impacted |
|  |  |  |  | Cellulose acetate filter | 92.37 |  |  |  |
|  |  |  |  | Fibre cotton combers | 92.17 | Papercup cellulosic | 0.96 |  |
|  |  |  |  | Fibre cotton Us pima | 89.50 |  |  |  |
|  |  |  |  | Cellulose | 88.73 | Cellulose | 0.92 |  |
|  |  |  |  | Fibre cotton uzbekistan | 88.56 |  |  |  |
|  |  |  |  | Fibre roasted flax | 87.07 | Cellulose | 0.92 |  |
|  |  |  |  | Fibre hemp fine | 86.00 |  |  |  |
|  |  |  |  | Fibre linen | 85.31 | Hydroxyethyl cellulose | 0.88 |  |
|  |  |  |  | Fibre hemp rough | 84.98 |  |  |  |
| BR-S | 26c | Fragment | Black | Polyethylene low density | 96.26 | HDPE | 0.92 | Synthetic |
|  |  |  |  | Polyethylene low density | 96.26 |  |  |  |
|  |  |  |  | Polyethylene low density | 96.13 | HDPE | 0.92 |  |
|  |  |  |  | Polyethylene low density | 96.11 |  |  |  |
|  |  |  |  | Polyethylene low density | 96.08 | HDPE | 0.92 |  |
|  |  |  |  | Polyethylene low density | 85.73 |  |  |  |
|  |  |  |  | Polyethylene low density | 95.47 | HDPE | 0.92 |  |
|  |  |  |  | Fibre thermoplastic elastomere | 95.45 |  |  |  |
|  |  |  |  | Polyethylene low density | 95.44 | HDPE | 0.92 |  |
|  |  |  |  | Polyethylene low density | 95.10 |  |  |  |
| BR-S | 30b | Fiber | White | Mask 128S GeATR | 95.97 | Cardboard/cellulose | 0.97 | Anthropogenically impacted |
|  |  |  |  | Cellulose acetate filter | 87.32 |  |  |  |
|  |  |  |  | Fibre cotton combers | 87.04 | Papercup cellulosic | 0.96 |  |
|  |  |  |  | Cellulose | 86.78 |  |  |  |
|  |  |  |  | Fibre cotton Us pima | 85.65 | Cellulose | 0.92 |  |
|  |  |  |  | Fibre cotton uzbekistan | 85.09 |  |  |  |
|  |  |  |  | Fibre roasted flax | 83.73 | Cellulose | 0.92 |  |
|  |  |  |  | Fibre linen | 81.62 |  |  |  |
|  |  |  |  | Fibre hemp fine | 81.34 | Cellulose | 0.88 |  |
|  |  |  |  | Fibre hemp rough | 81.24 |  |  |  |
| BR-S | C2a | Fiber | Blue | Cellophane | 82.16 | Cardboard/cellulose | 0.97 | Semi-Synthetic |
|  |  |  |  | Cellulose | 78.50 |  |  |  |
|  |  |  |  | Fibre viscose | 72.38 | Papercup cellulosic | 0.97 |  |
|  |  |  |  | Fibre viscose dyed | 69.51 |  |  |  |
|  |  |  |  | Cellophane | 64.90 | Cellulose | 0.92 |  |
|  |  |  |  | Fibre grass | 62.03 |  |  |  |
|  |  |  |  | Hydroxyethyl cellulose | 61.29 | Methyl cellulose | 0.92 |  |
|  |  |  |  | Cellulose | 60.58 |  |  |  |
|  |  |  |  | Wood mahagoni | 59.88 | Hydroxyethyl cellulose | 0.91 |  |
|  |  |  |  | Mask 128S GeATR | 59.25 |  |  |  |
| BR-S | C3a | Fiber | Blue | Mask 128S GeATR | 95.49 | Cardboard/cellulose | 0.97 | Anthropogenically impacted |
|  |  |  |  | Cellulose acetate filter | 93.42 |  |  |  |
|  |  |  |  | Fibre cotton combers | 92.92 | Papercup cellulosic | 0.96 |  |
|  |  |  |  | Fibre cotton Us pima | 90.58 |  |  |  |
|  |  |  |  | Fibre cotton uzbekistan | 89.74 | Cellulose | 0.92 |  |
|  |  |  |  | Celllulose | 89.63 |  |  |  |
|  |  |  |  | Fibre roasted flax | 88.06 | Cellulose | 0.92 |  |
|  |  |  |  | Fibre hemp fine | 87.69 |  |  |  |
|  |  |  |  | Fibre linen | 87.45 | Cellulose | 0.90 |  |
|  |  |  |  | Fibre hemp rough | 86.87 |  |  |  |
| BR-S | C3b | Fiebr | Black | Mask 128S GeATR | 94.16 | Cardboard/cellulose | 0.98 | Anthropogenically impacted |
|  |  |  |  | Cellulose acetate filter | 91.48 |  |  |  |
|  |  |  |  | Cellulose | 90.03 | Papercup cellulosic | 0.97 |  |
|  |  |  |  | Fibre cotton combers | 89.49 |  |  |  |
|  |  |  |  | Fibre cotton Us pima | 88.23 | Cellulose | 0.94 |  |
|  |  |  |  | Fibre hemp fine | 87.80 |  |  |  |
|  |  |  |  | Fibre linen | 87.79 | Cellulose | 0.94 |  |
|  |  |  |  | Fibre cotton uzbekistan | 87.49 |  |  |  |
|  |  |  |  | Fibre roasted flax | 86.28 | Cellulose | 0.91 |  |
|  |  |  |  | Fibre hemp rough | 86.11 |  |  |  |
| BR-A | 1a | Fiber | Clear | Mask 128S GeATR | 97.25 | Cardboard/cellulose | 0.98 | Anthropogenically impacted |
|  |  |  |  | Cellulose | 90.77 |  |  |  |
|  |  |  |  | Cellulose acetate filter | 90.48 | Papercup cellulosic | 0.97 |  |
|  |  |  |  | Fibre cotton combers | 90.21 |  |  |  |
|  |  |  |  | Fibre cotton Us pima | 88.87 | Cellulose | 0.92 |  |
|  |  |  |  | Fibre cotton uzbekistan | 88.49 |  |  |  |
|  |  |  |  | Fibre roasted flax | 86.70 | Cellulose | 0.92 |  |
|  |  |  |  | Fibre linen | 85.38 |  |  |  |
|  |  |  |  | Fibre hemp rough | 85.28 | Methyl cellulose | 0.89 |  |
|  |  |  |  | Fibre hemp fine | 85.20 |  |  |  |
| BR-A | 1b | Fiber | Clear | Mask 128S GeATR | 97.00 | Cardboard/cellulose | 0.98 | Anthropogenically impacted |
|  |  |  |  | Cellulose acetate filter | 92.71 |  |  |  |
|  |  |  |  | Fibre cotton combers | 91.68 | Papercup cellulosic | 0.97 |  |
|  |  |  |  | Cellulose | 90.19 |  |  |  |
|  |  |  |  | Fibre cotton Us pima | 89.61 | Cellulose | 0.94 |  |
|  |  |  |  | Fibre cotton Us pima | 88.67 |  |  |  |
|  |  |  |  | Fibre cotton uzbekistan | 87.41 | Cellulose | 0.94 |  |
|  |  |  |  | Fibre hemp fine | 86.71 |  |  |  |
|  |  |  |  | Fibre linen | 85.42 | Cellulose | 0.90 |  |
|  |  |  |  | Fibre hemp rough | 85.22 |  |  |  |
| BR-A | 2a | Fiber | Blue white | Mask 128S GeATR | 90.65 | Papercup cellulosic | 0.88 | Anthropogenically impacted |
|  |  |  |  | Fibre cotton combers | 81.74 |  |  |  |
|  |  |  |  | Cellulose acetate filter | 81.03 | Cardboard/cellulose | 0.88 |  |
|  |  |  |  | Cellulose | 79.97 |  |  |  |
|  |  |  |  | Fibre cotton Us pima | 79.33 | ethyl cellulose | 0.86 |  |
|  |  |  |  | Fibre cotton uzbekistan | 77.82 |  |  |  |
|  |  |  |  | Fibre roasted flax | 76.72 | Methyl cellulose | 0.86 |  |
|  |  |  |  | Fibre hemp fine | 74.90 |  |  |  |
|  |  |  |  | Fibre hemp rough | 74.01 | Hydroxyethyl cellulose | 0.85 |  |
|  |  |  |  | Fibre linen | 73.83 |  |  |  |
| BR-A | 2b | Fiber | Blue | Mask 128S GeATR | 96.65 | Cardboard/cellulose | 0.97 | Anthropogenically impacted |
|  |  |  |  | Cellulose acetate filter | 90.84 |  |  |  |
|  |  |  |  | Fibre cotton combers | 90.32 | Papercup cellulosic | 0.97 |  |
|  |  |  |  | Fibre cotton Us pima | 88.45 |  |  |  |
|  |  |  |  | Fibre cotton uzbekistan | 87.83 | Cellulose | 0.96 |  |
|  |  |  |  | Cellulose | 87.51 |  |  |  |
|  |  |  |  | Fibre roasted flax | 86.69 | Cellulose | 0.95 |  |
|  |  |  |  | Fibre linen | 84.07 |  |  |  |
|  |  |  |  | Fibre hemp fine | 83.97 | Cellulose | 0.91 |  |
|  |  |  |  | Fibre hemp rough | 83.39 |  |  |  |
| C-V-A | 1a | Fiber | Clear | Mask 128S GeATR | 95.54 | Cardboard/cellulose | 0.98 | Anthropogenically impacted |
|  |  |  |  | Cellulose | 90.17 |  |  |  |
|  |  |  |  | Cellulose acetate filter | 86.65 | Papercup cellulosic | 0.97 |  |
|  |  |  |  | Fibre cotton combers | 86.27 |  |  |  |
|  |  |  |  | Fibre cotton Us pima | 84.98 | Cellulose | 0.94 |  |
|  |  |  |  | Fibre cotton uzbekistan | 84.78 |  |  |  |
|  |  |  |  | Cellulose wipe | 84.30 | Cellulose | 0.93 |  |
|  |  |  |  | Fibre linen | 83.70 |  |  |  |
|  |  |  |  | Fibre hemp fine | 83.61 | Hydroxyethyl cellulose | 0.91 |  |
|  |  |  |  | Fibre roasted flax | 83.42 |  |  |  |
| C-V-A | 5a | Fiber | White | Mask 128S GeATR | 93.72 | Cardboard/cellulose | 0.96 | Anthropogenically impacted |
|  |  |  |  | Fibre cotton combers | 88.95 |  |  |  |
|  |  |  |  | Cellulose acetate filter | 88.18 | Papercup cellulosic | 0.96 |  |
|  |  |  |  | Fibre cotton Us pima | 85.77 |  |  |  |
|  |  |  |  | Cellulose | 85.52 | Cellulose | 0.91 |  |
|  |  |  |  | Fibre cotton uzbekistan | 84.46 |  |  |  |
|  |  |  |  | Fibre roasted flax | 82.93 | Cellulose | 0.91 |  |
|  |  |  |  | Fibre hemp fine | 81.56 |  |  |  |
|  |  |  |  | Fibre hemp rough | 80.92 | Hydroxyethyl cellulose | 0.88 |  |
|  |  |  |  | Fibre linen | 80.56 |  |  |  |
| C-V-A | 5b | Fiber | Blue white | Mask 128S GeATR | 95.67 | Cardboard/cellulose | 0.97 | Anthropogenically impacted |
|  |  |  |  | Cellulose acetate filter | 89.88 |  |  |  |
|  |  |  |  | Fibre cotton combers | 88.56 | Papercup cellulosic | 0.96 |  |
|  |  |  |  | Fibre cotton Us pima | 87.23 |  |  |  |
|  |  |  |  | Cellulose | 87.14 | Cellulose | 0.91 |  |
|  |  |  |  | Fibre cotton uzbekistan | 86.21 |  |  |  |
|  |  |  |  | Fibre roasted flax | 85.13 | Cellulose | 0.90 |  |
|  |  |  |  | Fibre hemp fine | 83.06 |  |  |  |
|  |  |  |  | Fibre linen | 82.18 | Methyl cellulose | 0.88 |  |
|  |  |  |  | Fibre hemp rough | 81.66 |  |  |  |
| LOP-A | 1a | Fiber | White | Mask 128S GeATR | 90.13 | Cardboard/cellulose | 0.96 | Anthropogenically impacted |
|  |  |  |  | Cellulose | 86.62 |  |  |  |
|  |  |  |  | Cellulose acetate filter | 86.42 | Papercup cellulosic | 0.95 |  |
|  |  |  |  | Fibre cotton combers | 85.57 |  |  |  |
|  |  |  |  | Fibre cotton Us pima | 85.18 | Cellulose | 0.89 |  |
|  |  |  |  | Cellulose wipe | 84.98 |  |  |  |
|  |  |  |  | Fibre linen | 84.94 | Cellulose | 0.89 |  |
|  |  |  |  | Fibre cotton uzbekistan | 94.91 |  |  |  |
|  |  |  |  | Wood mahagoni | 83.95 | Cellulose | 0.87 |  |
|  |  |  |  | Fibre hemp fine | 83.94 |  |  |  |
| LOP-A | 1b | Fiber | Black | Mask 128S GeATR | 96.15 | Cardboard/cellulose | 0.98 | Anthropogenically impacted |
|  |  |  |  | Cellulose acetate filter | 92.15 |  |  |  |
|  |  |  |  | Fibre cotton combers | 91.10 | Papercup cellulosic | 0.97 |  |
|  |  |  |  | cellulose | 90.06 |  |  |  |
|  |  |  |  | Fibre cotton Us pima | 89.02 | Cellulose | 0.94 |  |
|  |  |  |  | Fibre cotton uzbekistan | 87.97 |  |  |  |
|  |  |  |  | Fibre roasted flax | 87.21 | Cellulose | 0.94 |  |
|  |  |  |  | Fibre hemp fine | 87.06 |  |  |  |
|  |  |  |  | Fibre linen | 86.28 | Cellulose | 0.90 |  |
|  |  |  |  | Fibre hemp rough | 85.50 |  |  |  |
| LOP-A | 2a | Fiber | Blue | Mask 128S GeATR | 93.47 | Cardboard/cellulose | 0.96 | Anthropogenically impacted |
|  |  |  |  | Cellulose | 84.77 |  |  |  |
|  |  |  |  | Cellulose acetate filter | 84.39 | Papercup cellulosic | 0.95 |  |
|  |  |  |  | Fibre cotton combers | 84.24 |  |  |  |
|  |  |  |  | Fibre cotton Us pima | 82.23 | Cellulose | 0.92 |  |
|  |  |  |  | Fibre cotton uzbekistan | 81.21 |  |  |  |
|  |  |  |  | Fibre roasted flax | 79.81 | Cellulose | 0.91 |  |
|  |  |  |  | Fibre hemp fine | 78.66 |  |  |  |
|  |  |  |  | Fibre hemp rough | 77.88 | Cellulose | 0.88 |  |
|  |  |  |  | Fibre linen | 77.35 |  |  |  |
| LOP-A | 2b | Fiber | Black | Polyethylene terephthalate | 96.23 | Polyethylene terephthalate | 0.96 | Synthetic |
|  |  |  |  | Polyester | 95.31 |  |  |  |
|  |  |  |  | Polyethylene terephthalate | 94.92 | Polyesterterphthalate | 0.96 |  |
|  |  |  |  | Polyethylene terephthalate | 94.80 |  |  |  |
|  |  |  |  | Polyethylene terephthalate | 93.93 | Polyethylene terephthalate | 0.90 |  |
|  |  |  |  | Fibre polyester | 82.91 |  |  |  |
|  |  |  |  | Polyethylene terephthalate | 92.62 | Polyethylene terephthalate | 0.95 |  |
|  |  |  |  | Polyethylene terephthalate | 92.27 |  |  |  |
|  |  |  |  | fibre polyester | 91.41 | Polyethylene terephthalate | 0.95 |  |
|  |  |  |  | Polyester | 88.58 |  |  |  |
| LOP-A | 3a | Fiber | Whtie | Mask 128S GeATR | 92.43 | Cardboard/cellulose | 0.95 | Anthropogenically impacted |
|  |  |  |  | Fibre cotton combers | 83.83 |  |  |  |
|  |  |  |  | Cellulose acetate filter | 83.60 | Papercup cellulosic | 0.94 |  |
|  |  |  |  | Cellulose | 82.81 |  |  |  |
|  |  |  |  | Fibre cotton Us pima | 82.14 | Cellulose | 0.89 |  |
|  |  |  |  | Fibre cotton uzbekistan | 81.23 |  |  |  |
|  |  |  |  | Fibre roasted flax | 79.64 | Cellulose | 0.80 |  |
|  |  |  |  | Fibre hemp fine | 79.64 |  |  |  |
|  |  |  |  | Fibre hemp rough | 76.29 | Methyl cellulose | 0.88 |  |
|  |  |  |  | Fibre linen | 75.84 |  |  |  |
| PS-V | A2a | Fiber | White | Mask 128S Ge ATR #2 | 96.33 | Cardboard/cellulose | 0.95 | Anthropogenic origin |
|  |  |  |  | Cellulose acetate filter | 95.48 |  |  |  |
|  |  |  |  | Fibre cotton combers | 93.69 | Papercup cellulosic | 0.93 |  |
|  |  |  |  | Fibre cotton US pima | 92.52 |  |  |  |
|  |  |  |  | Fibre cotton uzbekistan | 92.24 | Cellulose | 0.92 |  |
|  |  |  |  | Fibre linen | 91.16 |  |  |  |
|  |  |  |  | Cellulose | 91.06 | Cellulose | 0.91 |  |
|  |  |  |  | Fibre roasted flax | 90.45 |  |  |  |
|  |  |  |  | Fibre hemp rough | 89.86 | Fibre viscose dyed | 0.90 |  |
|  |  |  |  |  |  |  |  |  |
| PS-V | A3b | Fiber | Black/White | Mask 128S Ge ATR #2 | 95.73 | Cardboard/cellulose | 0.97 | Anthropogenic origin |
|  |  |  |  | Fibre cotton combers | 90.38 |  |  |  |
|  |  |  |  | Cellulose acetate filter | 90.28 | Papercup cellulosic | 0.96 |  |
|  |  |  |  | Fibre cotton US pima | 88.55 |  |  |  |
|  |  |  |  | Cellulose | 88.36 | Cellulose | 0.91 |  |
|  |  |  |  | Fibre cotton uzbekistan | 87.78 |  |  |  |
|  |  |  |  | Fibre roasted flax | 85.43 | Cellulose | 0.91 |  |
|  |  |  |  | Fibre hemp fine | 83.68 |  |  |  |
|  |  |  |  | Fibre hemp rough | 83.64 | Cellulose | 0.88 |  |
|  |  |  |  |  |  |  |  |  |
| PS-V | 11a | Fiber | White | Wood mahagoni | 87.17 | Cardboard/cellulose | 0.96 | Natural |
|  |  |  |  | Fibre poplar down | 84.63 |  |  |  |
|  |  |  |  | Fibre poplar down | 83.24 | Papercup cellulosic | 0.95 |  |
|  |  |  |  | Mask 128S Ge ATR #2 | 82.82 |  |  |  |
|  |  |  |  | Fibre kapok | 81.26 | Cellulose | 0.89 |  |
|  |  |  |  | Wood pine | 81.18 |  |  |  |
|  |  |  |  | Wood beech | 80.79 | Cellulose | 0.88 |  |
|  |  |  |  | Fibre grass | 80.64 |  |  |  |
|  |  |  |  | Cellulose | 80.00 | Methyl cellulose | 0.88 |  |
|  |  |  |  |  |  |  |  |  |
| PS-V | 11b | Fiber | White | Mask 128S Ge ATR #2 | 91.95 | Cardboard/cellulose | 0.95 | Anthropogenic origin |
|  |  |  |  | Fibre cotton combers | 83.53 |  |  |  |
|  |  |  |  | Cellulose acetate filter | 82.95 | Papercup cellulosic | 0.95 |  |
|  |  |  |  | Cellulose | 81.78 |  |  |  |
|  |  |  |  | Fibre cotton US pima | 81.11 | Cellulose | 0.91 |  |
|  |  |  |  | Fibre cotton uzbekistan | 79.78 |  |  |  |
|  |  |  |  | Fibre roasted flax | 78.36 | Cellulose | 0.90 |  |
|  |  |  |  | Fibre hemp fine | 76.32 |  |  |  |
|  |  |  |  | Fibre hemp rough | 75.85 | Methyl cellulose | 0.88 |  |
|  |  |  |  | Fibre linen | 75.25 |  |  |  |
| PS-V | 14a | Fiber | Clear White | Mask 128S Ge ATR #2 | 96.95 | Cardboard/cellulose | 0.98 | Anthropogenic origin |
|  |  |  |  | Cellulose | 92.67 |  |  |  |
|  |  |  |  | Cellulose acetate filter | 91.94 | Papercup cellulosic | 0.97 |  |
|  |  |  |  | Fibre cotton combers | 91.33 |  |  |  |
|  |  |  |  | Fibre cotton US pima | 90.13 | Cellulose | 0.94 |  |
|  |  |  |  | Fibre cotton uzbekistan | 90.10 |  |  |  |
|  |  |  |  | Fibre linen | 90.04 | Cellulose | 0.94 |  |
|  |  |  |  | Cellulose wipe | 89.79 |  |  |  |
|  |  |  |  | Fibre hemp fine | 88.99 | Cellulose | 0.91 |  |
|  |  |  |  | Fibre hemp rough | 88.79 |  |  |  |
| PS-V | 14b | Fiber | White | Mask 128S Ge ATR #2 | 81.41 | Cardboard/cellulose | 0.96 | Natural |
|  |  |  |  | Wood mahagoni | 80.58 |  |  |  |
|  |  |  |  | Wood pine | 79.87 | Papercup cellulosic | 0.95 |  |
|  |  |  |  | Cellulose | 77.29 |  |  |  |
|  |  |  |  | Cellulose wipe | 75.63 | Cellulose | 0.88 |  |
|  |  |  |  | Fibre grass | 74.32 |  |  |  |
|  |  |  |  | Fibre poplar down | 74.08 | Cellulose | 0.87 |  |
|  |  |  |  | Cellophane | 74.03 |  |  |  |
|  |  |  |  | Fibre poplar down | 73.06 | Methyl cellulose | 0.86 |  |
|  |  |  |  | Fibre kapok | 72.54 |  |  |  |
| PS-V | 15c | Fiber | Blue | Polyethylene terephthalate | 97.10 | Polyesterterphthalate | 0.98 | Synthetic |
|  |  |  |  | Polyethylene terephthalate | 96.01 |  |  |  |
|  |  |  |  | Polyethylene terephthalate | 95.62 | Polyethylene terephthalate | 0.97 |  |
|  |  |  |  | Polyester | 95.56 |  |  |  |
|  |  |  |  | Polyethylene terephthalate | 95.49 | Polyethylene terephthalate | 0.97 |  |
|  |  |  |  | Fibre polyester | 94.28 |  |  |  |
|  |  |  |  | Polyethylene terephthalate | 83.42 | PET | 0.97 |  |
|  |  |  |  | Fibre polyester | 73.15 |  |  |  |
|  |  |  |  | Polyethylene terephthalate | 93.15 | Polyethylene terephthalate | 0.97 |  |
|  |  |  |  | Polyester | 90.77 |  |  |  |
| PS-S | 26a | Bundle | White | Cellulose | 88.43 | Cardboard/cellulose | 0.97 | Semi-synthetic |
|  |  |  |  | Fibre viscose | 81.57 |  |  |  |
|  |  |  |  | Cellophane | 79.86 | Papercup cellulosic | 0.96 |  |
|  |  |  |  | Fibre viscose dyed | 79.33 |  |  |  |
|  |  |  |  | Fibre grass | 71.88 | Cellulose | 0.91 |  |
|  |  |  |  | Wood mahagoni | 71.44 |  |  |  |
|  |  |  |  | Cellulose | 70.28 | Cellulose | 0.90 |  |
|  |  |  |  | Wood pine | 70.15 |  |  |  |
|  |  |  |  | Cellulose wipe | 69.64 | Hydroxyethyl cellulose | 0.89 |  |
|  |  |  |  | Hydroxyethyl cellulose | 66.90 |  |  |  |
| C-V | 2a | Fragment | White | Fibre polyester | 94.68 | PET | 0.94 | Synthetic |
|  |  |  |  | Polyester | 94.52 |  |  |  |
|  |  |  |  | Fibre polyester | 94.47 | PET | 0.93 |  |
|  |  |  |  | Polyethylene terephthalate | 91.79 |  |  |  |
|  |  |  |  | Polyethylene terephthalate | 91.33 | PET | 0.93 |  |
|  |  |  |  | Polyethylene terephthalate | 91.01 |  |  |  |
|  |  |  |  | Polyethylene terephthalate | 90.37 | PET | 0.93 |  |
|  |  |  |  | Polyethylene terephthalate | 90.20 |  |  |  |
|  |  |  |  | Polyethylene terephthalate | 90.10 | PET | 0.93 |  |
|  |  |  |  | Polyethylene terephthalate | 89.03 |  |  |  |
| C-V | 2b | Fragment | White | Mask 128S Ge ATR #2 | 95.86 | Cardboard/cellulose | 0.98 | Anthropogenic origin |
|  |  |  |  | Fibre cotton combers | 91.65 |  |  |  |
|  |  |  |  | Cellulose acetate filter | 91.04 | Papercup cellulosic | 0.97 |  |
|  |  |  |  | Fibre cotton US pima | 89.19 |  |  |  |
|  |  |  |  | Fibre cotton uzbekistan | 88.54 | Cellulose | 0.94 |  |
|  |  |  |  | Fibre linen | 88.50 |  |  |  |
|  |  |  |  | Cellulose | 88.01 | Cellulose | 0.94 |  |
|  |  |  |  | Fibre hemp rough | 87.12 |  |  |  |
|  |  |  |  | Fibre hemp fine | 86.94 | Cellulose | 0.90 |  |
|  |  |  |  | Fibre roasted flax | 86.23 |  |  |  |
| C-V | 3a | Fiber | Grey | Aramid | 86.84 | Aramid | 0.86 | Synthetic |
|  |  |  |  | Aramid | 85.85 |  |  |  |
|  |  |  |  | Carbon Disulfide | 40.54 | Aramid | 0.86 |  |
|  |  |  |  | Magnesium Acetylacetonate dihydrate | 33.37 |  |  |  |
|  |  |  |  | Amphetamine HCL in KBR | 33.19 | Fibre mulberry silk | 0.73 |  |
|  |  |  |  | Bromo dimethoxyamhetamine | 32.23 |  |  |  |
|  |  |  |  | D-Pantothenyl alcohol | 32.22 | Fibre silk slubbing | 0.72 |  |
|  |  |  |  | Diclofenac sodium | 31.87 |  |  |  |
|  |  |  |  | Polyamide | 31.05 | Fibre tussah silk | 0.72 |  |
|  |  |  |  | Polyamide | 30.53 |  |  |  |
| C-V | 5a | Fiber | Black/White | Mask 128S Ge ATR #2 | 88.91 | Cardboard/cellulose | 0.97 | Anthropogenic origin |
|  |  |  |  | Fibre cotton combers | 82.14 |  |  |  |
|  |  |  |  | Cellulose acetate filter | 81.78 | Papercup cellulosic | 0.96 |  |
|  |  |  |  | Fibre linen | 81.62 |  |  |  |
|  |  |  |  | Fibre cotton US pima | 80.93 | Cellulose | 0.93 |  |
|  |  |  |  | Fibre cotton uzbekistan | 80.01 |  |  |  |
|  |  |  |  | Fibre hemp rough | 78.90 | Cellulose | 0.92 |  |
|  |  |  |  | Cellulose | 78.39 |  |  |  |
|  |  |  |  | Fibre roasted flax | 78.29 | Methyl cellulose | 0.90 |  |
|  |  |  |  | Fibre hemp fine | 78.02 |  |  |  |
| C-V | 5b | Fiber | Black | Cellulose | 86.55 | Cardboard/cellulose | 0.98 | Semi-synthetic |
|  |  |  |  | Cellophane | 85.54 |  |  |  |
|  |  |  |  | Fibre viscose | 80.91 | Papercup cellulosic | 0.97 |  |
|  |  |  |  | Fibre viscose dyed | 78.62 |  |  |  |
|  |  |  |  | Fibre grass | 77.55 | Cellulose | 0.92 |  |
|  |  |  |  | Wood mahagoni | 75.16 |  |  |  |
|  |  |  |  | Wood pine | 73.30 | Cellulose | 0.91 |  |
|  |  |  |  | Cellulose wipe | 71.63 |  |  |  |
|  |  |  |  | Cellulose | 71.55 | Methyl cellulose | 0.91 |  |
|  |  |  |  | Fibre turf | 70.41 |  |  |  |
| C-V | 6a | Fiber | White | Mask 128S Ge ATR #2 | 97.49 | Cardboard/cellulose | 0.98 | Anthropogenic origin |
|  |  |  |  | Cellulose acetate filter | 92.88 |  |  |  |
|  |  |  |  | Fibre cotton combers | 92.68 | Papercup cellulosic | 0.97 |  |
|  |  |  |  | Cellulose | 92.55 |  |  |  |
|  |  |  |  | Fibre cotton US pima | 91.12 | Cellulose | 0.93 |  |
|  |  |  |  | Fibre cotton uzbekistan | 90.83 |  |  |  |
|  |  |  |  | Fibre linen | 90.31 | Cellulose | 0.93 |  |
|  |  |  |  | Cellulose wipe | 89.31 |  |  |  |
|  |  |  |  | Fibre hemp rough | 89.31 | Cellulose | 0.90 |  |
|  |  |  |  | Fibre hemp fine | 89.18 |  |  |  |
| C-V | 6b | Fiber | Clear | Mask 128S Ge ATR #2 | 92.44 | Cardboard/cellulose | 0.97 | Anthropogenic origin |
|  |  |  |  | Fibre cotton combers | 88.85 |  |  |  |
|  |  |  |  | Cellulose acetate filter | 87.91 | Papercup cellulosic | 0.96 |  |
|  |  |  |  | Fibre cotton US pima | 85.70 |  |  |  |
|  |  |  |  | Fibre cotton uzbekistan | 84.89 | Cellulose | 0.93 |  |
|  |  |  |  | Fibre linen | 84.85 |  |  |  |
|  |  |  |  | Cellulose | 84.05 | Cellulose | 0.93 |  |
|  |  |  |  | Fibre hemp fine | 83.39 |  |  |  |
|  |  |  |  | Fibre hemp rough | 83.22 | Hydroxyethyl cellulose | 0.89 |  |
|  |  |  |  | Fibre roasted flax | 83.18 |  |  |  |
| C-V | 6c | Fiber | Clear | Mask 128S Ge ATR #2 | 85.46 | Cardboard/cellulose | 0.96 | Anthropogenic origin |
|  |  |  |  | Fibre cotton combers | 80.67 |  |  |  |
|  |  |  |  | Cellulose acetate filter | 79.37 | Papercup cellulosic | 0.95 |  |
|  |  |  |  | Fibre cotton US pima | 77.59 |  |  |  |
|  |  |  |  | Fibre linen | 76.86 | Cellulose | 0.92 |  |
|  |  |  |  | Fibre cotton uzbekistan | 76.10 |  |  |  |
|  |  |  |  | Cellulose | 75.50 | Cellulose | 0.91 |  |
|  |  |  |  | Fibre hemp rough | 75.24 |  |  |  |
|  |  |  |  | Fibre hemp fine | 75.05 | leaf-plant | 0.89 |  |
|  |  |  |  | Fibre roasted flax | 74.59 |  |  |  |
| H-V | 2a | Fiber | Clear | Fibre grass | 76.34 | Cardboard/cellulose | 0.95 | Natural |
|  |  |  |  | Fibre linen | 71.49 |  |  |  |
|  |  |  |  | Fibre turf | 69.40 | Papercup cellulosic | 0.95 |  |
|  |  |  |  | Wood mahagoni | 67.84 |  |  |  |
|  |  |  |  | Calcium phosphate powder | 67.25 | leaf-plant | 0.89 |  |
|  |  |  |  | Wood pine | 66.36 |  |  |  |
|  |  |  |  | Wood beech | 66.18 | Cellulose | 0.89 |  |
|  |  |  |  | Calcium phosphate | 65.02 |  |  |  |
|  |  |  |  | Fibre kapok | 64.90 | Methyl cellulose | 0.88 |  |
|  |  |  |  | Fibre poplar down | 64.61 |  |  |  |
| H-V | 2b | Fiber | White | Fibre grass | 78.37 | Cardboard/cellulose | 0.96 | Anthropogenic origin |
|  |  |  |  | Cellophane | 74.78 |  |  |  |
|  |  |  |  | Fibre linen | 73.16 | Papercup cellulosic | 0.96 |  |
|  |  |  |  | Mask 128S Ge ATR #2 | 71.53 |  |  |  |
|  |  |  |  | Fibre linen | 71.18 | leaf-plant | 0.91 |  |
|  |  |  |  | Cellulose | 71.06 |  |  |  |
|  |  |  |  | Fibre flax | 70.37 | Cellulose | 0.90 |  |
|  |  |  |  | Wood mahagoni | 68.31 |  |  |  |
|  |  |  |  | Fibre viscose | 67.71 | Cellulose | 0.89 |  |
|  |  |  |  | Cellulose | 67.64 |  |  |  |
| H-V | 3a | Fiber | White | Mask 128S Ge ATR #2 | 97.89 | Cardboard/cellulose | 0.98 | Anthropogenic origin |
|  |  |  |  | Cellulose acetate filter | 92.32 |  |  |  |
|  |  |  |  | Fibre cotton combers | 91.34 | Papercup cellulosic | 0.97 |  |
|  |  |  |  | Fibre cotton US pima | 89.87 |  |  |  |
|  |  |  |  | Fibre cotton uzbekistan | 89.23 | Cellulose | 0.94 |  |
|  |  |  |  | Cellulose | 89.04 |  |  |  |
|  |  |  |  | Fibre roasted flax | 88.40 | Cellulose | 0.94 |  |
|  |  |  |  | Fibre linen | 86.39 |  |  |  |
|  |  |  |  | Fibre hemp fine | 86.05 | Cellulose | 0.90 |  |
|  |  |  |  | Fibre hemp rough | 85.28 |  |  |  |
| H-V | 3d | Fiber | Black | Polyethylene terephthalate | 94.01 | Polyesterterphthalate | 0.97 | Synthetic |
|  |  |  |  | Polyethylene terephthalate | 93.90 |  |  |  |
|  |  |  |  | Polyethylene terephthalate | 92.74 | Polyester | 0.96 |  |
|  |  |  |  | Polyethylene terephthalate | 92.55 |  |  |  |
|  |  |  |  | Polyethylene terephthalate | 92.54 | PET | 0.96 |  |
|  |  |  |  | Fibre polyester | 92.33 |  |  |  |
|  |  |  |  | Polyester | 91.48 | Polyethylene terephthalate | 0.96 |  |
|  |  |  |  | Polyethylene terephthalate | 91.42 |  |  |  |
|  |  |  |  | Fibre polyester | 90.34 | Polyesterterphthalate | 0.95 |  |
|  |  |  |  | Polyester | 88.49 |  |  |  |
| H-V | 5a | Fiber | White | Mask 128S Ge ATR #2 | 84.53 | Cardboard/cellulose | 0.97 | Anthropogenic origin |
|  |  |  |  | Cellulose | 78.38 |  |  |  |
|  |  |  |  | Fibre linen | 76.93 | Papercup cellulosic | 0.96 |  |
|  |  |  |  | Wood pine | 76.47 |  |  |  |
|  |  |  |  | Fibre cotton combers | 76.22 | Cellulose | 0.93 |  |
|  |  |  |  | Fibre cotton US pima | 75.64 |  |  |  |
|  |  |  |  | Fibre grass | 75.20 | Cellulose | 0.92 |  |
|  |  |  |  | Wood mahagoni | 75.11 |  |  |  |
|  |  |  |  | Fibre hemp rough | 75.04 | Methyl cellulose | 0.90 |  |
|  |  |  |  | Cellulose acetate filter | 74.86 |  |  |  |
| H-V | 5b | Fiber | Clear | Mask 128S Ge ATR #2 | 95.19 | Cardboard/cellulose | 0.97 | Anthropogenic origin |
|  |  |  |  | Fibre cotton combers | 89.53 |  |  |  |
|  |  |  |  | Cellulose acetate filter | 89.35 | Papercup cellulosic | 0.96 |  |
|  |  |  |  | Fibre cotton US pima | 87.15 |  |  |  |
|  |  |  |  | Fibre cotton uzbekistan | 86.04 | Cellulose | 0.92 |  |
|  |  |  |  | Fibre roasted flax | 84.98 |  |  |  |
|  |  |  |  | Cellulose | 84.96 | Cellulose | 0.92 |  |
|  |  |  |  | Fibre linen | 84.74 |  |  |  |
|  |  |  |  | Fibre hemp fine | 83.15 | Methyl cellulose | 0.89 |  |
|  |  |  |  | Fibre hemp rough | 83.10 |  |  |  |
| H-V | 6a | Fiber | White | Cellulose wipe | 89.76 | Cardboard/cellulose | 0.98 | Anthropogenic origin |
|  |  |  |  | Mask 128S Ge ATR #2 | 89.73 |  |  |  |
|  |  |  |  | Cellulose | 88.09 | Papercup cellulosic | 0.98 |  |
|  |  |  |  | Fibre linen | 85.44 |  |  |  |
|  |  |  |  | Fibre hemp fine | 84.09 | Cellulose | 0.93 |  |
|  |  |  |  | Cellulose acetate filter | 83.97 |  |  |  |
|  |  |  |  | Fibre grass | 83.85 | Cellulose | 0.93 |  |
|  |  |  |  | Fibre hemp fine | 83.26 |  |  |  |
|  |  |  |  | Fibre cotton uzbekistan | 82.05 | Methyl cellulose | 0.91 |  |
|  |  |  |  | Fibre cotton US pima | 81.54 |  |  |  |
| H-V | 6b | Fiber | White | Wood pine | 86.88 | Cardboard/cellulose | 0.97 | Natural |
|  |  |  |  | Wood mahagoni | 85.79 |  |  |  |
|  |  |  |  | Fibre poplar down | 79.74 | Papercup cellulosic | 0.96 |  |
|  |  |  |  | Fibre poplar down | 78.81 |  |  |  |
|  |  |  |  | Fibre turf | 78.51 | Cellulose | 0.93 |  |
|  |  |  |  | Mask 128S Ge ATR #2 | 78.45 |  |  |  |
|  |  |  |  | Fibre kapok | 75.57 | Cellulose | 0.93 |  |
|  |  |  |  | Cellulose | 75.51 |  |  |  |
|  |  |  |  | Fibre grass | 74.37 | Hydroxyethyl cellulose | 0.89 |  |
|  |  |  |  | Wood beech | 73.81 |  |  |  |
| H-V | 8a |  | Clear | Cellophane | 82.38 | Cardboard/cellulose | 0.98 | Semi-synthetic |
|  |  |  |  | Cellulose | 81.57 |  |  |  |
|  |  |  |  | Wood mahagoni | 80.88 | Papercup cellulosic | 0.97 |  |
|  |  |  |  | Fibre grass | 80.65 |  |  |  |
|  |  |  |  | Mask 128S Ge ATR #2 | 79.99 | Cellulose | 0.92 |  |
|  |  |  |  | Cellulose | 79.76 |  |  |  |
|  |  |  |  | Wood pine | 79.41 | Cellulose | 0.92 |  |
|  |  |  |  | Cellulose wipe | 78.07 |  |  |  |
|  |  |  |  | Fibre viscose | 77.69 | Methyl cellulose | 0.90 |  |
|  |  |  |  | Fibre linen | 76.45 |  |  |  |
| H-V | 8b | Fiber | White | Mask 128S Ge ATR #2 | 96.05 | Cardboard/cellulose | 0.97 | Anthropogenic origin |
|  |  |  |  | Cellulose acetate filter | 88.68 |  |  |  |
|  |  |  |  | Fibre cotton combers | 88.12 | Papercup cellulosic | 0.96 |  |
|  |  |  |  | Cellulose | 87.35 |  |  |  |
|  |  |  |  | Fibre cotton US pima | 86.14 | Cellulose | 0.93 |  |
|  |  |  |  | Fibre cotton uzbekistan | 85.16 |  |  |  |
|  |  |  |  | Fibre roasted flax | 84.35 | Cellulose | 0.92 |  |
|  |  |  |  | Fibre hemp fine | 82.81 |  |  |  |
|  |  |  |  | Fibre linen | 82.36 | Hydroxyethyl cellulose | 0.89 |  |
|  |  |  |  | Fibre hemp rough | 81.97 |  |  |  |
| H-V | 11a | Fiber | White | Mask 128S Ge ATR #2 | 93.62 | Cardboard/cellulose | 0.98 | Anthropogenic origin |
|  |  |  |  | Cellulose | 87.37 |  |  |  |
|  |  |  |  | Fibre linen | 86.37 | Papercup cellulosic | 0.97 |  |
|  |  |  |  | Cellulose acetate filter | 85.92 |  |  |  |
|  |  |  |  | Fibre cotton combers | 85.57 | Cellulose | 0.93 |  |
|  |  |  |  | Fibre cotton US pima | 84.50 |  |  |  |
|  |  |  |  | Fibre hemp fine | 84.25 | Cellulose | 0.92 |  |
|  |  |  |  | Fibre hemp rough | 84.05 |  |  |  |
|  |  |  |  | Fibre cotton uzbekistan | 83.76 | Methyl cellulose | 0.90 |  |
|  |  |  |  | Cellulose wipe | 83.10 |  |  |  |
| H-V | 11b | Fiber | Brown | Mask 128S Ge ATR #2 | 94.62 | Cardboard/cellulose | 0.98 | Anthropogenic origin |
|  |  |  |  | Cellulose | 89.39 |  |  |  |
|  |  |  |  | Cellulose acetate filter | 87.89 | Papercup cellulosic | 0.97 |  |
|  |  |  |  | Fibre cotton combers | 87.68 |  |  |  |
|  |  |  |  | Fibre linen | 86.86 | Cellulose | 0.92 |  |
|  |  |  |  | Fibre cotton US pima | 86.66 |  |  |  |
|  |  |  |  | Cellulose wipe | 86.61 | Cellulose | 0.92 |  |
|  |  |  |  | Fibre cotton uzbekistan | 86.23 |  |  |  |
|  |  |  |  | Fibre hemp fine | 85.34 | Hydroxyethyl cellulose | 0.89 |  |
|  |  |  |  | Fibre hemp rough | 84.95 |  |  |  |
| H-V | 13a | Fiber | White | Mask 128S Ge ATR #2 | 96.91 | Cardboard/cellulose | 0.97 | Anthropogenic origin |
|  |  |  |  | Cellulose acetate filter | 90.32 |  |  |  |
|  |  |  |  | Fibre cotton combers | 89.65 | Papercup cellulosic | 0.96 |  |
|  |  |  |  | Cellulose | 87.92 |  |  |  |
|  |  |  |  | Fibre cotton US pima | 87.75 | Cellulose | 0.95 |  |
|  |  |  |  | Fibre cotton uzbekistan | 87.00 |  |  |  |
|  |  |  |  | Fibre roasted flax | 86.21 | Cellulose | 0.94 |  |
|  |  |  |  | Fibre hemp fine | 84.80 |  |  |  |
|  |  |  |  | Fibre linen | 84.48 | Fibre kapok | 0.91 |  |
|  |  |  |  | Fibre hemp rough | 83.79 |  |  |  |
| H-V | 13b | Fiber | Clear | Mask 128S Ge ATR #2 | 97.10 | Cardboard/cellulose | 0.98 | Anthropogenic origin |
|  |  |  |  | Cellulose acetate filter | 94.59 |  |  |  |
|  |  |  |  | Fibre cotton combers | 94.03 | Papercup cellulosic | 0.97 |  |
|  |  |  |  | Fibre cotton US pima | 91.56 |  |  |  |
|  |  |  |  | Fibre cotton uzbekistan | 90.75 | Cellulose | 0.95 |  |
|  |  |  |  | Cellulose | 90.34 |  |  |  |
|  |  |  |  | Fibre roasted flax | 89.46 | Cellulose | 0.95 |  |
|  |  |  |  | Fibre hemp fine | 88.52 |  |  |  |
|  |  |  |  | Fibre linen | 88.30 | Cellulose | 0.91 |  |
|  |  |  |  | Fibre hemp rough | 87.71 |  |  |  |
| H-V | 15a | Fiber | White | Mask 128S Ge ATR #2 | 88.64 | Cardboard/cellulose | 0.95 | Anthropogenic origin |
|  |  |  |  | Cellulose | 78.30 |  |  |  |
|  |  |  |  | Fibre linen | 78.21 | Papercup cellulosic | 0.95 |  |
|  |  |  |  | Fibre cotton combers | 78.07 |  |  |  |
|  |  |  |  | Cellulose acetate filter | 77.33 | Cellulose | 0.89 |  |
|  |  |  |  | Fibre cotton US pima | 76.83 |  |  |  |
|  |  |  |  | Fibre hemp rough | 75.93 | Cellulose | 0.88 |  |
|  |  |  |  | Fibre cotton uzbekistan | 75.89 |  |  |  |
|  |  |  |  | Fibre hemp fine | 75.47 | leaf-plant | 0.85 |  |
|  |  |  |  | Fibre roasted flax | 74.23 |  |  |  |
| LAJ | 1b | Fiber | Clear | Mask 128S Ge ATR #2 | 94.52 | Cardboard/cellulose | 0.98 | Anthropogenic origin |
|  |  |  |  | Cellulose | 87.09 |  |  |  |
|  |  |  |  | Fibre cotton combers | 86.64 | Papercup cellulosic | 0.97 |  |
|  |  |  |  | Cellulose acetate filter | 86.06 |  |  |  |
|  |  |  |  | Fibre cotton US pima | 84.23 | Cellulose | 0.94 |  |
|  |  |  |  | Fibre cotton uzbekistan | 83.71 |  |  |  |
|  |  |  |  | Fibre linen | 82.84 | Cellulose | 0.93 |  |
|  |  |  |  | Fibre hemp fine | 82.64 |  |  |  |
|  |  |  |  | Cellulose wipe | 82.17 | Fibre poplar down | 0.92 |  |
|  |  |  |  | Fibre roasted flax | 82.07 |  |  |  |
| LAJ | 3a | Fiber | Black/White | Mask 128S Ge ATR #2 | 95.37 | Cardboard/cellulose | 0.98 | Anthropogenic origin |
|  |  |  |  | Fibre cotton combers | 85.76 |  |  |  |
|  |  |  |  | Cellulose acetate filter | 85.69 | Papercup cellulosic | 0.97 |  |
|  |  |  |  | Fibre cotton US pima | 84.58 |  |  |  |
|  |  |  |  | Cellulose | 84.52 | Cellulose | 0.93 |  |
|  |  |  |  | Fibre cotton uzbekistan | 84.12 |  |  |  |
|  |  |  |  | Fibre roasted flax | 82.62 | Cellulose | 0.92 |  |
|  |  |  |  | Fibre linen | 82.19 |  |  |  |
|  |  |  |  | Fibre hemp rough | 81.30 | Cellulose | 0.91 |  |
|  |  |  |  | Fibre hemp fine | 80.80 |  |  |  |
| LAJ | 3b | Fiber | Grey | Fibre grass | 80.24 | Cardboard/cellulose | 0.98 | Semi-synthetic |
|  |  |  |  | Cellulose | 80.05 |  |  |  |
|  |  |  |  | Fibre viscose | 75.08 | Papercup cellulosic | 0.97 |  |
|  |  |  |  | Wood mahagoni | 75.03 |  |  |  |
|  |  |  |  | Mask 128S Ge ATR #2 | 73.64 | Cellulose | 0.93 |  |
|  |  |  |  | Wood pine | 73.49 |  |  |  |
|  |  |  |  | Fibre viscose dyed | 73.03 | Cellulose | 0.92 |  |
|  |  |  |  | Fibre linen | 72.66 |  |  |  |
|  |  |  |  | Cellulose | 72.13 | Cellulose | 0.91 |  |
|  |  |  |  | Fibre turf | 71.97 |  |  |  |
| LAJ | 5a | Fiber | Black | Cotton Blue fiber | 96.19 | Cardboard/cellulose | 0.97 | Anthropogenic origin |
|  |  |  |  | Cotton Brown Fiber | 96.11 |  |  |  |
|  |  |  |  | Cotton Yellow Fiber | 95.33 | Papercup cellulosic | 0.96 |  |
|  |  |  |  | Cotton Black Fiber | 95.14 |  |  |  |
|  |  |  |  | Mask 128S Ge ATR #2 | 94.85 | Cellulose | 0.93 |  |
|  |  |  |  | Cotton Purple Fiber | 93.91 |  |  |  |
|  |  |  |  | Cotton Blue fiber | 93.56 | Fibre poplar down | 0.92 |  |
|  |  |  |  | Cotton Pink fiber Bundle | 93.30 |  |  |  |
|  |  |  |  | Cotton Blue fiber | 92.61 | Fibre poplar down | 0.92 |  |
|  |  |  |  | Cotton Green Fiber | 91.59 |  |  |  |
| LAJ | 5b | Fiber | Clear | PEVA Yellow Fiber | 88.13 | Vinyl chloride vinyl acetate hydroxypropyl acrylate | 0.86 | Synthetic |
|  |  |  |  | PEVA Green Fiber | 88.76 |  |  |  |
|  |  |  |  | PEVA Purple Fiber | 83.89 | Vinyl chloride vinyl acetate maleic acid | 0.83 |  |
|  |  |  |  | PEVA Pink Fiber | 78.63 |  |  |  |
|  |  |  |  | PEVA Blue Fiber | 76.05 | Alkyd varish | 0.83 |  |
|  |  |  |  | Polyvinyl acetate ethylene | 75.99 |  |  |  |
|  |  |  |  | Polyvinyl acetate ethylene | 74.16 | Vinyl chloride vinyl acetate | 0.82 |  |
|  |  |  |  | Geranyl acetate | 71.78 |  |  |  |
|  |  |  |  | Trans farnesyl acetate | 71.34 | Coal | 0.82 |  |
|  |  |  |  | Polyvinyl acetate ethylene | 70.29 |  |  |  |
| LAJ | 6a | Fiber | Clear | Cotton Grey Fiber | 81.41 | Cardboard/cellulose | 0.97 | Anthropogenic origin |
|  |  |  |  | Cotton Pink fiber Bundle | 80.32 |  |  |  |
|  |  |  |  | Fibre grass | 80.17 | Papercup cellulosic | 0.96 |  |
|  |  |  |  | Cotton Green Fiber | 79.13 |  |  |  |
|  |  |  |  | Mask 128S Ge ATR #2 | 79.06 | Hydroxyethyl cellulose | 0.95 |  |
|  |  |  |  | Cotton Yellow Fiber | 78.49 |  |  |  |
|  |  |  |  | Fibre linen | 76.94 | Fibre poplar down | 0.95 |  |
|  |  |  |  | Cotton Grey Fiber | 76.67 |  |  |  |
|  |  |  |  | Cotton Purple Fiber | 76.09 | Fibre poplar down | 0.95 |  |
|  |  |  |  | Cotton Red Fabric | 75.24 |  |  |  |
| LAJ | 6b | Fiber | Black | Cellulose | 90.03 | Cardboard/cellulose | 0.97 | Semi-synthetic |
|  |  |  |  | Fibre viscose | 82.34 |  |  |  |
|  |  |  |  | Cellophane | 82.01 | Papercup cellulosic | 0.97 |  |
|  |  |  |  | Cotton Pink fiber | 81.94 |  |  |  |
|  |  |  |  | Fibre viscose dyed | 79.55 | Cellulose | 0.92 |  |
|  |  |  |  | Cotton Green Fiber | 77.18 |  |  |  |
|  |  |  |  | Cotton Grey Fiber | 76.47 | Cellulose | 0.91 |  |
|  |  |  |  | Fibre grass | 74.67 |  |  |  |
|  |  |  |  | Cotton Yellow Fiber | 73.83 | Fibre poplar down | 0.90 |  |
|  |  |  |  | Hydroxyethyl cellulose | 72.89 |  |  |  |
| LAJ | 9a | Fiber | Clear White | Cotton Black Fiber | 96.09 | Cardboard/cellulose | 0.97 | Anthropogenic origin |
|  |  |  |  | Cotton Yellow Fiber | 95.49 |  |  |  |
|  |  |  |  | Cotton Blue fiber | 94.50 | Papercup cellulosic | 0.96 |  |
|  |  |  |  | Mask 128S Ge ATR #2 | 94.36 |  |  |  |
|  |  |  |  | Cotton Brown Fiber | 93.53 | Cellulose | 0.92 |  |
|  |  |  |  | Cotton Pink fiber Bundle | 93.21 |  |  |  |
|  |  |  |  | Cotton Green Fiber | 93.08 | Cellulose | 0.92 |  |
|  |  |  |  | Cotton Blue fiber | 91.56 |  |  |  |
|  |  |  |  | Cotton Blue fiber | 90.76 | Hydroxyethyl cellulose | 0.90 |  |
|  |  |  |  | Cotton Purple Fiber | 90.16 |  |  |  |
| LAJ | 9b | Fiber | Clear | Mask 128S Ge ATR #2 | 80.88 | Cardboard/cellulose | 0.96 | Anthropogenic origin |
|  |  |  |  | Cotton Brown Fiber | 80.78 |  |  |  |
|  |  |  |  | Cotton Blue fiber | 79.69 | Papercup cellulosic | 0.96 |  |
|  |  |  |  | Cotton Pink fiber Bundle | 79.49 |  |  |  |
|  |  |  |  | Cotton Yellow Fiber | 79.12 | Cellulose | 0.94 |  |
|  |  |  |  | Cotton Black Fiber | 79.04 |  |  |  |
|  |  |  |  | Cotton Blue fiber | 77.40 | Cellulose | 0.93 |  |
|  |  |  |  | Cotton Green Fiber | 77.34 |  |  |  |
|  |  |  |  | Cotton Green Fiber | 77.12 | Hydroxyethyl cellulose | 0.92 |  |
|  |  |  |  | Cotton Blue fiber | 76.96 |  |  |  |
| LAJ | 11a | Fiber | White | Cotton Black Fiber | 97.01 | Cardboard/cellulose | 0.98 | Anthropogenic origin |
|  |  |  |  | Mask 128S Ge ATR #2 | 96.54 |  |  |  |
|  |  |  |  | Cotton Yellow Fiber | 96.50 | Papercup cellulosic | 0.97 |  |
|  |  |  |  | Cotton Blue fiber | 95.97 |  |  |  |
|  |  |  |  | Cotton Brown Fiber | 95.16 | Cellulose | 0.93 |  |
|  |  |  |  | Cotton Green Fiber | 93.80 |  |  |  |
|  |  |  |  | Cotton Pink fiber Bundle | 93.64 | Cellulose | 0.93 |  |
|  |  |  |  | Cotton Blue fiber | 92.73 |  |  |  |
|  |  |  |  | Cotton Blue fiber | 92.42 | Hydroxyethyl cellulose | 0.92 |  |
|  |  |  |  | Cotton Purple Fiber | 91.68 |  |  |  |
| LAJ | 11b | Fiber | White | Cotton Black Fiber | 86.66 | Cardboard/cellulose | 0.97 | Anthropogenic origin |
|  |  |  |  | Cotton Pink fiber Bundle | 86.18 |  |  |  |
|  |  |  |  | Cotton Blue fiber | 85.64 | Papercup cellulosic | 0.96 |  |
|  |  |  |  | Cotton Yellow Fiber | 85.55 |  |  |  |
|  |  |  |  | Cotton Brown Fiber | 85.55 | Methyl cellulose | 0.92 |  |
|  |  |  |  | Cotton Blue fiber | 83.99 |  |  |  |
|  |  |  |  | Mask 128S Ge ATR #2 | 83.56 | Cellulose | 0.92 |  |
|  |  |  |  | Cotton Blue fiber | 82.59 |  |  |  |
|  |  |  |  | Cotton Green Fiber | 82.31 | Hydroxyethyl cellulose | 0.91 |  |
|  |  |  |  | Cotton Purple Fiber | 82.28 |  |  |  |
| LAJ | 11c | Fiber | Clear | Cotton Pink fiber Bundle | 91.58 | Cardboard/cellulose | 0.97 | Anthropogenic origin |
|  |  |  |  | Cotton Brown Fiber | 90.86 |  |  |  |
|  |  |  |  | Cotton Blue fiber | 90.59 | Papercup cellulosic | 0.96 |  |
|  |  |  |  | Cotton Black Fiber | 90.26 |  |  |  |
|  |  |  |  | Cotton Yellow Fiber | 89.41 | Methyl cellulose | 0.91 |  |
|  |  |  |  | Cotton Blue fiber | 88.50 |  |  |  |
|  |  |  |  | Cotton Blue fiber | 87.44 | Cellulose | 0.91 |  |
|  |  |  |  | Cotton Purple Fiber | 87.43 |  |  |  |
|  |  |  |  | Mask 128S Ge ATR #2 | 87.27 | Hydroxyethyl cellulose | 0.91 |  |
|  |  |  |  | Cotton Green Fiber | 85.88 |  |  |  |
| LAJ | 15a | Fiber | Clear | Cotton Green Fiber | 76.52 | Cardboard/cellulose | 0.92 | Anthropogenic origin |
|  |  |  |  | Cotton Pink fiber Bundle | 75.31 |  |  |  |
|  |  |  |  | Cotton Grey Fiber | 73.52 | Papercup cellulosic | 0.91 |  |
|  |  |  |  | Cotton Pink fiber Bundle | 73.16 |  |  |  |
|  |  |  |  | Cotton Black Fiber | 7307.00 | leaf-plant | 0.91 |  |
|  |  |  |  | Cotton Yellow Fiber | 72.28 |  |  |  |
|  |  |  |  | Cotton Brown Fiber | 71.74 | Methyl cellulose | 0.90 |  |
|  |  |  |  | Mask 128S Ge ATR #2 | 71.57 |  |  |  |
|  |  |  |  | Cotton Blue fiber | 71.01 | hydroxypropyl methyl cellulose | 0.89 |  |
|  |  |  |  | Cotton Green Fiber | 70.64 |  |  |  |
| LAJ | 15b | Fiber | Clear | Mask 128S Ge ATR #2 | 83.54 | Cardboard/cellulose | 0.97 | Anthropogenic origin |
|  |  |  |  | Cotton Brown Fiber | 81.45 |  |  |  |
|  |  |  |  | Cotton Blue fiber | 80.72 | Papercup cellulosic | 0.96 |  |
|  |  |  |  | Cotton Yellow Fiber | 80.72 |  |  |  |
|  |  |  |  | Cotton Black Fiber | 80.11 | leaf-plant | 0.91 |  |
|  |  |  |  | Cotton Green Fiber | 79.22 |  |  |  |
|  |  |  |  | Cotton Green Fiber | 79.01 | Cellulose | 0.91 |  |
|  |  |  |  | Cotton Purple Fiber | 79.00 |  |  |  |
|  |  |  |  | Cotton Blue fiber | 78.61 | Cellulose | 0.91 |  |
|  |  |  |  | Fibre linen | 78.57 |  |  |  |
| LC-V | 4a | Fiber | Blue | Cotton Black Fiber | 96.33 | Cardboard/cellulose | 0.97 | Anthropogenic origin |
|  |  |  |  | Cotton Yellow Fiber | 95.89 |  |  |  |
|  |  |  |  | Cotton Blue fiber | 95.44 | Papercup cellulosic | 0.96 |  |
|  |  |  |  | Mask 128S Ge ATR #2 | 95.09 |  |  |  |
|  |  |  |  | Cotton Brown Fiber | 94.60 | Cellulose | 0.92 |  |
|  |  |  |  | Cotton Blue fiber | 92.93 |  |  |  |
|  |  |  |  | Cotton Green Fiber | 92.90 | Cellulose | 0.92 |  |
|  |  |  |  | Cotton Pink fiber Bundle | 92.61 |  |  |  |
|  |  |  |  | Cotton Blue fiber | 91.79 | Fibre poplar down | 0.90 |  |
|  |  |  |  | Cotton Purple Fiber | 91.16 |  |  |  |
| LC-V | 4b | Fiber | White | Cotton pink fiber | 91.14 | Cardboard/cellulose | 0.97 | Anthropogenic origin |
|  |  |  |  | Cellulose | 88.61 |  |  |  |
|  |  |  |  | Cotton Green Fiber | 88.34 | Papercup cellulosic | 0.97 |  |
|  |  |  |  | Cotton Grey Fiber | 86.81 |  |  |  |
|  |  |  |  | Cotton Yellow Fiber | 86.58 | Hydroxyethyl cellulose | 0.91 |  |
|  |  |  |  | Fibre viscose | 83.44 |  |  |  |
|  |  |  |  | Cotton Grey Fiber | 80.39 | Cellulose | 0.91 |  |
|  |  |  |  | Cellophane | 79.82 |  |  |  |
|  |  |  |  | Fibre viscose dyed | 79.81 | Cellulose | 0.91 |  |
|  |  |  |  | Cotton Purple Fiber | 78.87 |  |  |  |
| LC-V | 1a | Fiber | White | Cotton Pink Fiber | 89.11 | Cardboard/cellulose | 0.97 | Anthropogenic origin |
|  |  |  |  | Cotton Green Fiber | 86.52 |  |  |  |
|  |  |  |  | Cotton Yellow Fiber | 85.03 | Papercup cellulosic | 0.95 |  |
|  |  |  |  | Cotton Grey Fiber | 84.58 |  |  |  |
|  |  |  |  | Cellulose | 83.46 | C. wipe | 0.92 |  |
|  |  |  |  | Fibre viscose | 81.81 |  |  |  |
|  |  |  |  | Cotton Grey Fiber | 80.20 | Fibre viscose dyed | 0.91 |  |
|  |  |  |  | Cotton Purple Fiber | 79.15 |  |  |  |
|  |  |  |  | Fibre viscose dyed | 76.08 | Cellulose | 0.91 |  |
|  |  |  |  | Cotton White Lab Coat Fiber | 75.60 |  |  |  |
| P-FIBER | 1b | Fiber | Orange | PET White Fragment | 96.69 | Polyethylene terephthalate | 0.97 | Synthetic |
|  |  |  |  | PET White Fiber Bundle | 95.84 |  |  |  |
|  |  |  |  | PET Blue Fiber Bundle | 95.47 | Polyesterterphthalate | 0.97 |  |
|  |  |  |  | Polyethylene terephthalate | 95.31 |  |  |  |
|  |  |  |  | Polyester White Label Fiber | 95.28 | Polyethylene terephthalate | 0.96 |  |
|  |  |  |  | PET White Fiber Bundle | 95.03 |  |  |  |
|  |  |  |  | PET Blue Fragment | 94.59 | PET | 0.96 |  |
|  |  |  |  | PET Yellow Fiber Bundle | 94.56 |  |  |  |
|  |  |  |  | Polyester | 94.54 | Polyethylene terephthalate | 0.96 |  |
|  |  |  |  | Polyester Green Label Fiber | 94.41 |  |  |  |
| LOP | 1a | Fiber | Black | Cotton Black Fiber | 95.96 | Cardboard/cellulose | 0.96 | Anthropogenic origin |
|  |  |  |  | Cotton Yellow Fiber | 94.28 |  |  |  |
|  |  |  |  | Mask 128S Ge ATR #2 | 93.24 | Papercup cellulosic | 0.96 |  |
|  |  |  |  | Cotton Pink fiber Bundle | 92.36 |  |  |  |
|  |  |  |  | Cotton Blue fiber | 92.36 | Methyl cellulose | 0.92 |  |
|  |  |  |  | Cotton Green Fiber | 91.95 |  |  |  |
|  |  |  |  | Cotton Black Fiber | 90.64 | Cellulose | 0.92 |  |
|  |  |  |  | Cotton Brown Fiber | 90.57 |  |  |  |
|  |  |  |  | Cotton White Lab Coat Fiber | 89.38 | Hydroxyethyl cellulose | 0.91 |  |
|  |  |  |  | Cotton Blue fiber | 89.31 |  |  |  |
| LOP | 1b | Fiber | Yellow | Mask 128S Ge ATR #2 | 90.28 | Cardboard/cellulose | 0.97 | Anthropogenic origin |
|  |  |  |  | Cotton Black Fiber | 89.91 |  |  |  |
|  |  |  |  | Cotton Brown Fiber | 89.52 | Papercup cellulosic | 0.97 |  |
|  |  |  |  | Cotton Yellow Fiber | 89.32 |  |  |  |
|  |  |  |  | Cotton Blue fiber | 89.31 | Cellulose | 0.94 |  |
|  |  |  |  | Cotton Pink fiber Bundle | 87.61 |  |  |  |
|  |  |  |  | Cotton Green Fiber | 87.17 | Cellulose | 0.93 |  |
|  |  |  |  | Cotton Blue fiber | 86.13 |  |  |  |
|  |  |  |  | Cotton Blue fiber | 85.73 | Hydroxyethyl cellulose | 0.93 |  |
|  |  |  |  | Cotton Purple Fiber | 85.60 |  |  |  |
| LOP | 3a | Fiber | Black | Polyester Brown Yarn Fiber | 96.82 | Polyamide | 0.68 | Synthetic |
|  |  |  |  | PET Yellow Fiber Bundle | 96.31 |  |  |  |
|  |  |  |  | Polyester White Label Fiber | 95.99 | Nylon | 0.65 |  |
|  |  |  |  | PET White Fiber Bundle | 95.93 |  |  |  |
|  |  |  |  | Polyester white Fiber | 95.85 | Polyethylene | 0.61 |  |
|  |  |  |  | Polyester Blue Yarn Fiber | 95.29 |  |  |  |
|  |  |  |  | Polyester Pink Fiber | 94.99 | Polyethylene | 0.60 |  |
|  |  |  |  | PET Green Fiebr | 94.84 |  |  |  |
|  |  |  |  | Polyester White fiber | 94.83 | Nylon | 0.59 |  |
|  |  |  |  | PET Blue Fragment | 94.81 |  |  |  |
| LOP | 4a | Fiber | Clear | Mask 128S Ge ATR #2 | 78.50 | Cardboard/cellulose | 0.97 | Anthropogenic origin |
|  |  |  |  | Fibre linen | 75.25 |  |  |  |
|  |  |  |  | Cotton Yellow Fiber | 74.79 | Papercup cellulosic | 0.96 |  |
|  |  |  |  | Fibre linen | 74.39 |  |  |  |
|  |  |  |  | Cotton Blue fiber | 73.24 | Hydroxyethyl cellulose | 0.92 |  |
|  |  |  |  | Fibre flax | 73.06 |  |  |  |
|  |  |  |  | Cotton Brown Fiber | 73.00 | Cellulose | 0.91 |  |
|  |  |  |  | Cotton Yellow Fiber | 72.44 |  |  |  |
|  |  |  |  | Cotton Green Fiber | 72.44 | Methyl cellulose | 0.91 |  |
|  |  |  |  | Cotton pink fiber | 72.32 |  |  |  |
| LOP | 5a | fiber | Black | Mask 128S Ge ATR #2 | 95.00 | Cardboard/cellulose | 0.98 | Anthropogenic origin |
|  |  |  |  | Cotton Yellow Fiber | 93.27 |  |  |  |
|  |  |  |  | Cotton Blue fiber | 92.11 | Papercup cellulosic | 0.97 |  |
|  |  |  |  | Cotton Black Fiber | 91.44 |  |  |  |
|  |  |  |  | Cotton Brown Fiber | 91.19 | Cellulose | 0.94 |  |
|  |  |  |  | Cotton Yellow Fiber | 90.94 |  |  |  |
|  |  |  |  | Cotton Purple Fiber | 90.74 | Cellulose | 0.94 |  |
|  |  |  |  | Cotton Green Fiber | 90.49 |  |  |  |
|  |  |  |  | Cotton Blue fiber | 90.05 | Fibre poplar down | 0.93 |  |
|  |  |  |  | Cotton Green Fiber | 89.57 |  |  |  |
| LOP | 5b | fiber | White | Mask 128S Ge ATR #2 | 97.15 | Cardboard/cellulose | 0.98 | Anthropogenic origin |
|  |  |  |  | Cotton Brown Fiber | 96.27 |  |  |  |
|  |  |  |  | Cotton Blue fiber | 96.00 | Papercup cellulosic | 0.97 |  |
|  |  |  |  | Cotton Yellow Fiber | 95.38 |  |  |  |
|  |  |  |  | Cotton Black Fiber | 94.28 | Cellulose | 0.95 |  |
|  |  |  |  | Cotton Purple Fiber | 94.13 |  |  |  |
|  |  |  |  | Cotton Blue fiber | 92.93 | Cellulose | 0.95 |  |
|  |  |  |  | Cotton Blue fiber | 92.53 |  |  |  |
|  |  |  |  | Cotton Green Fiber | 92.34 | Fibre poplar down | 0.93 |  |
|  |  |  |  | Cotton Yellow Fiber | 91.77 |  |  |  |
| LOP | 6a | Fiber | Black | PET Yellow Fiber Bundle | 97.92 | Polyesterterphthalate | 0.95 | Synthetic |
|  |  |  |  | Polyester Red Yarn Fiber | 97.82 |  |  |  |
|  |  |  |  | Polyester Green Label Fiber | 97.56 | Polyester | 0.94 |  |
|  |  |  |  | Polyester White Label Fiber | 97.50 |  |  |  |
|  |  |  |  | Polyester Blue Yarn Fiber | 97.35 | Polyethylene terephthalate | 0.94 |  |
|  |  |  |  | PET White Fiber Bundle | 97.02 |  |  |  |
|  |  |  |  | Polyester White fiber | 96.87 | PET | 0.94 |  |
|  |  |  |  | Polyester white fabric bag fiber | 96.69 |  |  |  |
|  |  |  |  | Polyester Pink Fiber | 96.67 | Polyester | 0.94 |  |
|  |  |  |  | PET Blue Fragment | 96.66 |  |  |  |
| LOP | 6b | Fiber | Black | Cotton pink fiber | 83.02 | Cardboard/cellulose | 0.98 | Anthropogenic origin |
|  |  |  |  | Cotton Grey Fiber | 77.73 |  |  |  |
|  |  |  |  | Cotton Green Fiber | 77.16 | Papercup cellulosic | 0.97 |  |
|  |  |  |  | Cotton Yellow Fiber | 77.16 |  |  |  |
|  |  |  |  | Cotton Grey Fiber | 75.92 | Methyl cellulose | 0.92 |  |
|  |  |  |  | Cellophane | 75.21 |  |  |  |
|  |  |  |  | Celluose | 75.15 | Hydroxyethyl cellulose | 0.92 |  |
|  |  |  |  | Cotton Red Fabric | 74.47 |  |  |  |
|  |  |  |  | Fibre grass | 74.09 | Cellulose | 0.91 |  |
|  |  |  |  | Fibre viscose | 70.87 |  |  |  |
| LOP | 7a | Fiber | White | Mask 128S Ge ATR #2 | 97.03 | Cardboard/cellulose | 0.97 | Anthropogenic origin |
|  |  |  |  | Cotton Blue fiber | 95.07 |  |  |  |
|  |  |  |  | Cotton Brown Fiber | 94.83 | Papercup cellulosic | 0.96 |  |
|  |  |  |  | Cotton Purple Fiber | 94.45 |  |  |  |
|  |  |  |  | Cotton Yellow Fiber | 93.94 | Cellulose wipe | 0.93 |  |
|  |  |  |  | Cotton Yellow Fiber | 93.15 |  |  |  |
|  |  |  |  | Cotton Green Fiber | 92.43 | Cellulose | 0.93 |  |
|  |  |  |  | Cotton Blue fiber | 92.20 |  |  |  |
|  |  |  |  | Cotton Black Fiber | 91.90 | Cellulose | 0.93 |  |
|  |  |  |  | Cellulose acetate filter | 91.55 |  |  |  |
| LOP | 7b | fiber | White | Mask 128S Ge ATR #2 | 89.59 | Cardboard/cellulose | 0.98 | Anthropogenic origin |
|  |  |  |  | Cotton Yellow Fiber | 89.23 |  |  |  |
|  |  |  |  | Cotton Blue fiber | 89.11 | Papercup cellulosic | 0.97 |  |
|  |  |  |  | Cotton Grey Fiber | 88.89 |  |  |  |
|  |  |  |  | Cotton Brown Fiber | 88.61 | Cellulose | 0.93 |  |
|  |  |  |  | Cotton Black Fiber | 88.24 |  |  |  |
|  |  |  |  | Cotton Purple Fiber | 88.00 | Hydroxyethyl cellulose | 0.92 |  |
|  |  |  |  | Cotton Yellow Fiber | 87.82 |  |  |  |
|  |  |  |  | Cotton Blue fiber | 87.33 | Methyl cellulose | 0.92 |  |
|  |  |  |  | Cotton Green Fiber | 86.82 |  |  |  |
| LOP | 10a | Fiber | Clear | Cotton Black Fiber | 91.94 | Cardboard/cellulose | 0.97 | Anthropogenic origin |
|  |  |  |  | Cotton Yellow Fiber | 91.38 |  |  |  |
|  |  |  |  | Mask 128S Ge ATR #2 | 91.05 | Papercup cellulosic | 0.96 |  |
|  |  |  |  | Cotton pink fiber | 91.01 |  |  |  |
|  |  |  |  | Cotton Blue fiber | 91.00 | Cellulose | 0.94 |  |
|  |  |  |  | Cotton Brown Fiber | 90.88 |  |  |  |
|  |  |  |  | Cotton Green Fiber | 88.58 | Celllulose | 0.93 |  |
|  |  |  |  | Cotton Blue fiber | 88.36 |  |  |  |
|  |  |  |  | Cotton Blue fiber | 87.96 | Hydroxyethyl cellulose | 0.93 |  |
|  |  |  |  | Cotton Purple Fiber | 87.05 |  |  |  |
| LOP | 10b | Fiber | White | Mask 128S Ge ATR #2 | 90.97 | Cardboard/cellulose | 0.97 | Anthropogenic origin |
|  |  |  |  | Cotton Yellow Fiber | 89.79 |  |  |  |
|  |  |  |  | Cotton Black Fiber | 89.11 | Papercup cellulosic | 0.96 |  |
|  |  |  |  | Cotton Blue fiber | 88.41 |  |  |  |
|  |  |  |  | Cotton Green Fiber | 87.64 | Cellulose | 0.95 |  |
|  |  |  |  | Cotton Brown Fiber | 87.42 |  |  |  |
|  |  |  |  | Cotton Yellow Fiber | 86.28 | Hydroxyethyl cellulose | 0.94 |  |
|  |  |  |  | Cotton Blue fiber | 86.25 |  |  |  |
|  |  |  |  | Cotton Black Fiber | 85.78 | Methyl cellulose | 0.94 |  |
|  |  |  |  | Cotton White Lab Coat Fiber | 85.64 |  |  |  |
| LC-S | 1a | fiber | Black | Cotton Green Fiber | 76.41 | Cardboard/cellulose | 0.96 | Anthropogenic origin |
|  |  |  |  | Cotton Yellow Fiber | 74.26 |  |  |  |
|  |  |  |  | Cotton pink fiber | 74.01 | Papercup cellulosic | 0.95 |  |
|  |  |  |  | Cotton Grey Fiber | 73.83 |  |  |  |
|  |  |  |  | Cellulose | 72.92 | Cellulose wipe | 0.93 |  |
|  |  |  |  | Fibre viscose | 71.86 |  |  |  |
|  |  |  |  | Cotton White Lab Coat Fiber | 71.71 | Fibre viscose dyed | 0.93 |  |
|  |  |  |  | Cotton Purple Fiber | 70.02 |  |  |  |
|  |  |  |  | Cellulose | 68.83 | Cellulose | 0.93 |  |
|  |  |  |  | Mask 128S Ge ATR #2 | 68.52 |  |  |  |
| LC-S | 1b | Fiber | Clear | Cotton pink fiber | 67.03 | Cardboard/cellulose | 0.96 | Anthropogenic origin |
|  |  |  |  | Cotton Green Fiber | 66.90 |  |  |  |
|  |  |  |  | Cellulose | 65.39 | Papercup cellulosic | 0.95 |  |
|  |  |  |  | Cotton Grey Fiber | 65.00 |  |  |  |
|  |  |  |  | Cotton Yellow Fiber | 64.75 | Cellulose | 0.94 |  |
|  |  |  |  | Fibre viscose | 62.36 |  |  |  |
|  |  |  |  | Cellophane | 60.88 | Cellulose | 0.94 |  |
|  |  |  |  | Cotton White Lab Coat Fiber | 60.85 |  |  |  |
|  |  |  |  | Fibre viscose dyed | 58.28 | Cellulose | 0.93 |  |
|  |  |  |  | Cellulose | 57.97 |  |  |  |
| LC-S | 5a | Fiber | White | Cotton pink fiber | 87.07 | Cardboard/cellulose | 0.98 | Anthropogenic origin |
|  |  |  |  | Cotton Green Fiber | 81.84 |  |  |  |
|  |  |  |  | Cotton Grey Fiber | 81.75 | Papercup cellulosic | 0.98 |  |
|  |  |  |  | Cotton Yellow Fiber | 81.61 |  |  |  |
|  |  |  |  | Fibre grass | 80.42 | Hydroxyethyl cellulose | 0.94 |  |
|  |  |  |  | Cotton Grey Fiber | 80.39 |  |  |  |
|  |  |  |  | Cellulose | 78.13 | Methyl cellulose | 0.94 |  |
|  |  |  |  | Cotton Red Fabric | 77.70 |  |  |  |
|  |  |  |  | Cellophane | 77.43 | Cellulose | 0.94 |  |
|  |  |  |  | Fibre viscose | 76.15 |  |  |  |
| LC-S | 5b | Fiber | Clear | Polyester Green Label Fiber | 97.10 | Polyethylene terephthalate | 0.95 | Synthetic |
|  |  |  |  | Polyester White Label Fiber | 97.06 |  |  |  |
|  |  |  |  | PET White Fiber Bundle | 97.02 | Polyesterterphthalate | 0.95 |  |
|  |  |  |  | PET Yellow Fiber Bundle | 96.90 |  |  |  |
|  |  |  |  | PET Blue Fragment | 96.46 | Polyethylene terephthalate | 95.00 |  |
|  |  |  |  | Polyester Blue Yarn Fiber | 93.36 |  |  |  |
|  |  |  |  | PET White Fragment | 96.32 | PET | 0.94 |  |
|  |  |  |  | PET Orange Fiber | 96.23 |  |  |  |
|  |  |  |  | Polyester white fiber | 96.16 | Polyethylene terephthalate | 0.94 |  |
|  |  |  |  | Polyester Dark Brown Yarn Fiber | 95.80 |  |  |  |
| LC-S | 10a | Fiber | Clear | Mask 128S Ge ATR #2 | 98.35 | Cardboard/cellulose | 0.98 | Anthropogenic origin |
|  |  |  |  | Cotton Blue fiber | 79.46 |  |  |  |
|  |  |  |  | Cotton Yellow Fiber | 97.13 | Papercup cellulosic | 0.97 |  |
|  |  |  |  | Cotton Brown Fiber | 96.97 |  |  |  |
|  |  |  |  | Cotton Black Fiber | 95.94 | Cellulose | 0.96 |  |
|  |  |  |  | Cotton Blue fiber | 94.84 |  |  |  |
|  |  |  |  | Cotton Purple Fiber | 94.72 | Cellulose | 0.96 |  |
|  |  |  |  | Cotton Green Fiber | 94.28 |  |  |  |
|  |  |  |  | Cotton Blue fiber | 93.97 | Cellulose wipe | 0.94 |  |
|  |  |  |  | Cellulose acetate filter | 92.44 |  |  |  |
| LC-S | 10b | fiber | Black | Cotton Brown Fiber | 97.25 | Cardboard/cellulose | 0.98 | Anthropogenic origin |
|  |  |  |  | Cotton Blue fiber | 97.14 |  |  |  |
|  |  |  |  | Cotton Purple Fiber | 96.02 | Papercup cellulosic | 0.97 |  |
|  |  |  |  | Cotton Yellow Fiber | 95.85 |  |  |  |
|  |  |  |  | Cotton Blue fiber | 95.40 | Cellulose | 0.93 |  |
|  |  |  |  | Cotton Yellow Fiber | 95.34 |  |  |  |
|  |  |  |  | Cotton Black Fiber | 94.93 | Cellulose | 0.93 |  |
|  |  |  |  | Mask 128S Ge ATR #2 | 94.83 |  |  |  |
|  |  |  |  | Cotton Blue fiber | 94.00 | Hydroxyethyl cellulose | 0.92 |  |
|  |  |  |  | Cotton Green Fiber | 93.68 |  |  |  |
| LC-S | 12a | Fiber | Black | Polyethylene terephthalate | 96.75 | Polyethylene terephthalate | 0.97 | Synthetic |
|  |  |  |  | PET White Fragment | 96.65 |  |  |  |
|  |  |  |  | Polyester White Label Fiber | 96.33 | Polyesterterphthalate | 0.97 |  |
|  |  |  |  | Polyester red Fiber | 96.21 |  |  |  |
|  |  |  |  | Polyester Green Label Fiber | 96.13 | Polyethylene terephthalate | 0.97 |  |
|  |  |  |  | PET Yellow Fiber Bundle | 96.04 |  |  |  |
|  |  |  |  | Polyester Blue Yarn Fiber | 96.01 | PET | 0.97 |  |
|  |  |  |  | Polyester Red Yarn Fiber | 95.82 |  |  |  |
|  |  |  |  | Polyester | 95.79 | Polyethylene terephthalate | 0.96 |  |
|  |  |  |  | PET Black Fiber | 95.49 |  |  |  |
| LC-S | 12b | Fiber | Black | Cotton Black Fiber | 72.10 | Cardboard/cellulose | 0.95 | Anthropogenic origin |
|  |  |  |  | Cotton White Lab Coat Fiber | 71.59 |  |  |  |
|  |  |  |  | Cotton Black Fiber | 71.33 | Papercup cellulosic | 0.95 |  |
|  |  |  |  | Cotton Yellow Fiber | 69.82 |  |  |  |
|  |  |  |  | Mask 128S Ge ATR #2 | 69.19 | Cellulose | 0.94 |  |
|  |  |  |  | Cellophane | 67.55 |  |  |  |
|  |  |  |  | Cotton Green Fiber | 67.22 | Hydroxyethyl cellulose | 0.93 |  |
|  |  |  |  | Cotton Pink fiber Bundle | 67.01 |  |  |  |
|  |  |  |  | Cotton Blue fiber | 66.93 | Cellulose | 0.93 |  |
|  |  |  |  | Cotton Blue fiber | 65.74 |  |  |  |
| LC-S | 14a | Fiber | Black | Cellulose | 91.72 | Cardboard/cellulose | 0.97 | Semi-synthetic |
|  |  |  |  | Cotton pink fiber | 86.35 |  |  |  |
|  |  |  |  | Fibre viscose | 86.33 | Papercup cellulosic | 0.96 |  |
|  |  |  |  | Fibre viscose dyed | 82.98 |  |  |  |
|  |  |  |  | Cotton Green Fiber | 81.72 | Fibre viscose dyed | 0.93 |  |
|  |  |  |  | Cotton Grey Fiber | 80.76 |  |  |  |
|  |  |  |  | Cellophane | 79.66 | Cellulose wipe | 0.93 |  |
|  |  |  |  | Cotton Yellow Fiber | 78.63 |  |  |  |
|  |  |  |  | Cotton Grey Fiber | 73.79 | Cellulose | 0.93 |  |
|  |  |  |  | Fibre grass | 73.06 |  |  |  |
| LC-S | 14b | Fiber | White | Mask 128S Ge ATR #2 | 96.74 | Cardboard/cellulose | 0.98 | Anthropogenic origin |
|  |  |  |  | Cotton Yellow Fiber | 96.47 |  |  |  |
|  |  |  |  | Cotton Blue fiber | 96.30 | Papercup cellulosic | 0.97 |  |
|  |  |  |  | Cotton Black Fiber | 96.22 |  |  |  |
|  |  |  |  | Cotton Brown Fiber | 95.46 | Cellulose | 0.92 |  |
|  |  |  |  | Cotton Purple Fiber | 93.63 |  |  |  |
|  |  |  |  | Cotton Green Fiber | 93.30 | Cellulose | 0.92 |  |
|  |  |  |  | Cotton Blue fiber | 92.63 |  |  |  |
|  |  |  |  | Cotton Blue fiber | 92.39 | Methyl cellulose | 0.91 |  |
|  |  |  |  | Cellulose acetate filter | 92.01 |  |  |  |
| LC-S | 17a | fiber | Blue | Cotton Black Fiber | 75.31 | Cardboard/cellulose | 0.96 | Anthropogenic origin |
|  |  |  |  | Cotton Yellow Fiber | 74.23 |  |  |  |
|  |  |  |  | Mask 128S Ge ATR #2 | 73.14 | Papercup cellulosic | 0.96 |  |
|  |  |  |  | Cotton Blue fiber | 72.75 |  |  |  |
|  |  |  |  | Cotton White Lab Coat Fiber | 72.36 | Methyl cellulose | 0.91 |  |
|  |  |  |  | Cotton Pink fiber Bundle | 71.67 |  |  |  |
|  |  |  |  | Cotton Green Fiber | 70.97 | Hydroxyethyl cellulose | 0.90 |  |
|  |  |  |  | Cotton Brown Fiber | 70.86 |  |  |  |
|  |  |  |  | Cotton Yellow Fiber | 70.50 | Cellulose | 0.90 |  |
|  |  |  |  | Cotton Purple Fiber | 70.38 |  |  |  |
| LC-S | 20b | Fiber | Black | Cotton Blue fiber | 97.76 | Cardboard/cellulose | 0.98 | Anthropogenic origin |
|  |  |  |  | Cotton Brown Fiber | 97.60 |  |  |  |
|  |  |  |  | Mask 128S Ge ATR #2 | 97.41 | Papercup cellulosic | 0.97 |  |
|  |  |  |  | Cotton Yellow Fiber | 96.04 |  |  |  |
|  |  |  |  | Cotton Black Fiber | 95.44 | Cellulose | 0.95 |  |
|  |  |  |  | Cotton Blue fiber | 95.05 |  |  |  |
|  |  |  |  | Cotton Purple Fiber | 94.58 | Cellulose | 0.94 |  |
|  |  |  |  | Cotton Green Fiber | 93.59 |  |  |  |
|  |  |  |  | Cotton Blue fiber | 93.41 | Cellulose wipe | 0.93 |  |
|  |  |  |  | Cellulose acetate filter | 93.10 |  |  |  |
| LC-S | 23a | Fiber | Black | Cotton pink fiber | 85.30 | Cardboard/cellulose | 0.98 | Semi-synthetic |
|  |  |  |  | Cellulose | 84.29 |  |  |  |
|  |  |  |  | Cellophane | 82.89 | Papercup cellulosic | 0.97 |  |
|  |  |  |  | Cotton Green Fiber | 81.23 |  |  |  |
|  |  |  |  | Cotton Grey Fiber | 79.43 | Hydroxyethyl cellulose | 0.92 |  |
|  |  |  |  | Cotton Yellow Fiber | 78.22 |  |  |  |
|  |  |  |  | Fibre viscose | 77.78 | Methyl cellulose | 0.92 |  |
|  |  |  |  | Fibre viscose dyed | 75.68 |  |  |  |
|  |  |  |  | Fibre grass | 75.20 | Cellulose | 0.92 |  |
|  |  |  |  | Cotton Grey Fiber | 74.23 |  |  |  |
| LC-S | 23b | Fiber | White | PET Blue Fragment | 97.52 | Polyesterterphthalate | 0.95 | Synthetic |
|  |  |  |  | PET White Fiber Bundle | 97.43 |  |  |  |
|  |  |  |  | Polyester White Label Fiber | 97.31 | Polyethylene terephthalate | 0.95 |  |
|  |  |  |  | PET White Fragment | 97.26 |  |  |  |
|  |  |  |  | Polyester Red Yarn Fiber | 96.97 | Polyethylene terephthalate | 0.95 |  |
|  |  |  |  | PET Yellow Fiber Bundle | 96.89 |  |  |  |
|  |  |  |  | Polyester Green Label Fiber | 96.87 | PET | 0.94 |  |
|  |  |  |  | Polyester Pink Fiber | 96.63 |  |  |  |
|  |  |  |  | PET Green Fiebr | 96.53 | Polyethylene terephthalate | 0.94 |  |
|  |  |  |  | PET White Fiber Bundle | 96.37 |  |  |  |
| LC-S | 25a | fiber | White | Cotton Brown Fiber | 97.29 | Cardboard/cellulose | 0.97 | Anthropogenic origin |
|  |  |  |  | Cotton Blue fiber | 96.93 |  |  |  |
|  |  |  |  | Cotton Yellow Fiber | 95.46 | Papercup cellulosic | 0.96 |  |
|  |  |  |  | Mask 128S Ge ATR #2 | 95.33 |  |  |  |
|  |  |  |  | Cotton Purple Fiber | 95.06 | Cellulose | 0.92 |  |
|  |  |  |  | Cotton Black Fiber | 94.97 |  |  |  |
|  |  |  |  | Cotton Blue fiber | 94.28 | Cellulose | 0.92 |  |
|  |  |  |  | Cotton Blue fiber | 93.33 |  |  |  |
|  |  |  |  | Cotton Pink fiber Bundle | 92.90 | Cellulose wipe | 0.92 |  |
|  |  |  |  | Cotton Green Fiber | 91.81 |  |  |  |
| LC-S | 25b | Fiber | Black | Polyethylene terephthalate | 94.86 | Polyethylene terephthalate | 0.97 | Synthetic |
|  |  |  |  | Polyethylene terephthalate | 93.83 |  |  |  |
|  |  |  |  | Polyester | 9.61 | Polyethylene terephthalate | 0.96 |  |
|  |  |  |  | Polyester red Fiber | 93.56 |  |  |  |
|  |  |  |  | Polyethylene terephthalate | 93.26 | Polyethylene terephthalate | 0.96 |  |
|  |  |  |  | PET White Fragment | 93.25 |  |  |  |
|  |  |  |  | PET Blue Fiber Bundle | 93.19 | Polyethylene terephthalate | 0.95 |  |
|  |  |  |  | Polyethylene terephthalate | 91.96 |  |  |  |
|  |  |  |  | Polyester Blue Yarn Fiber | 91.64 | PET | 0.95 |  |
|  |  |  |  | PET Yellow Fiber Bundle | 91.51 |  |  |  |
| LC-S | 27a | Fiber | Clear/Yellow | Cotton pink fiber | 78.75 | Cardboard/cellulose | 0.98 | Possibly Natural |
|  |  |  |  | Fibre grass | 78.03 |  |  |  |
|  |  |  |  | Cotton Grey Fiber | 75.50 | Papercup cellulosic | 0.97 |  |
|  |  |  |  | Cotton Green Fiber | 73.46 |  |  |  |
|  |  |  |  | Fibre turf | 73.28 | Methyl cellulose | 0.93 |  |
|  |  |  |  | Cotton Yellow Fiber | 72.83 |  |  |  |
|  |  |  |  | Wood pine | 72.80 | Hydroxyethyl cellulose | 0.92 |  |
|  |  |  |  | Cotton Red Fabric | 72.75 |  |  |  |
|  |  |  |  | Wood mahagoni | 72.72 | Cellulose | 0.92 |  |
|  |  |  |  | Cellophane | 72.55 |  |  |  |
| LC-S | 27b | Fiber | White | PET Black fragment | 97.10 | Polyesterterphthalate | 0.97 | Synthetic |
|  |  |  |  | Polyester Blue Yarn Fiber | 97.02 |  |  |  |
|  |  |  |  | PET green Glitter Fragment | 96.87 | Polyethylene terephthalate | 0.96 |  |
|  |  |  |  | Polyester red fiber | 96.81 |  |  |  |
|  |  |  |  | Polyester Green Label Fiber | 96.81 | PET | 0.96 |  |
|  |  |  |  | PET red film | 96.77 |  |  |  |
|  |  |  |  | Polyester white fiber | 96.72 | Polyethylene terephthalate | 0.96 |  |
|  |  |  |  | PET Yellow Fiber Bundle | 96.64 |  |  |  |
|  |  |  |  | Polyester White Label Fiber | 96.61 | Polyester | 0.95 |  |
|  |  |  |  | Polyester Dark Brown Yarn Fiber | 96.18 |  |  |  |
| LC-S | 29a | Fiber | White | Mask 128S Ge ATR #2 | 95.24 | Cardboard/cellulose | 0.98 | Anthropogenic origin |
|  |  |  |  | Cotton Blue fiber | 94.54 |  |  |  |
|  |  |  |  | Cotton Brown Fiber | 94.44 | Papercup cellulosic | 0.97 |  |
|  |  |  |  | Cotton Yellow Fiber | 94.08 |  |  |  |
|  |  |  |  | Cotton Black Fiber | 93.39 | Cellulose | 0.93 |  |
|  |  |  |  | Cotton Purple Fiber | 92.49 |  |  |  |
|  |  |  |  | Cotton Blue fiber | 91.77 | Celllulose | 0.92 |  |
|  |  |  |  | Cotton Blue fiber | 91.70 |  |  |  |
|  |  |  |  | Cotton Yellow Fiber | 91.23 | Hydroxyethyl cellulose | 0.91 |  |
|  |  |  |  | Cotton Green Fiber | 91.15 |  |  |  |
| LC-S | 29b | fiber | White | Cotton pink fiber | 90.85 | Cardboard/cellulose | 0.98 | Anthropogenic origin |
|  |  |  |  | Cotton Green Fiber | 88.75 |  |  |  |
|  |  |  |  | Cotton Yellow Fiber | 87.37 | Papercup cellulosic | 0.97 |  |
|  |  |  |  | Cotton Grey Fiber | 86.77 |  |  |  |
|  |  |  |  | Cotton Grey Fiber | 84.69 | Hydroxyethyl cellulose | 0.92 |  |
|  |  |  |  | Fibre grass | 83.33 |  |  |  |
|  |  |  |  | Wood mahagoni | 81.83 | Methyl cellulose | 0.92 |  |
|  |  |  |  | Cellophane | 81.17 |  |  |  |
|  |  |  |  | Cotton Purple Fiber | 80.48 | Cellulose | 0.92 |  |
|  |  |  |  | Wood pine | 80.04 |  |  |  |
| LC-S | A2a | Fiber | Clear | Cotton Black Fiber | 97.79 | Cardboard/cellulose | 0.97 | Anthropogenic origin |
|  |  |  |  | Cotton Yellow Fiber | 97.08 |  |  |  |
|  |  |  |  | Cotton Blue fiber | 97.05 | Papercup cellulosic | 0.96 |  |
|  |  |  |  | Cotton Brown Fiber | 96.24 |  |  |  |
|  |  |  |  | Cotton Pink fiber Bundle | 96.01 | Cellullose | 0.93 |  |
|  |  |  |  | Mask 128S Ge ATR #2 | 95.42 |  |  |  |
|  |  |  |  | Cotton Blue fiber | 94.50 | Celllulose | 0.93 |  |
|  |  |  |  | Cotton Green Fiber | 93.54 |  |  |  |
|  |  |  |  | Cotton Blue fiber | 93.41 | Fibre poplar down | 0.91 |  |
|  |  |  |  | Cotton Purple Fiber | 92.24 |  |  |  |
| LC-S | C1a | Fiber | Yellow | Cotton Yellow Fiber | 95.15 | Cardboard/cellulose | 0.98 | Anthropogenic origin |
|  |  |  |  | Mask 128S Ge ATR #2 | 95.03 |  |  |  |
|  |  |  |  | Cotton Black Fiber | 94.97 | Papercup cellulosic | 0.97 |  |
|  |  |  |  | Cotton Blue fiber | 93.85 |  |  |  |
|  |  |  |  | Cotton Blue fiber | 92.10 | Cellulose | 0.93 |  |
|  |  |  |  | Cotton Green Fiber | 92.01 |  |  |  |
|  |  |  |  | Cotton Brown Fiber | 91.92 | Methyl cellulose | 0.93 |  |
|  |  |  |  | Cotton White Lab Coat Fiber | 90.95 |  |  |  |
|  |  |  |  | Cotton Blue fiber | 90.28 | Hydroxyethyl cellulose | 0.92 |  |
|  |  |  |  | Cotton Purple Fiber | 89.70 |  |  |  |
| LC-S | C2a | Fragment | Yellow | Fur wild boar | 86.40 | Fur red deer | 0.75 | Keratin Based |
|  |  |  |  | Fur Cow | 81.80 |  |  |  |
|  |  |  |  | FW86-LH | 81.49 | Fur angora rabbit | 0.71 |  |
|  |  |  |  | Scoured wool not made rough | 80.41 |  |  |  |
|  |  |  |  | Wool raw cashmere afghanistan | 80.10 | Fur cat european shorthair | 0.71 |  |
|  |  |  |  | zein purified | 79.47 |  |  |  |
|  |  |  |  | wool raw cashmere mongolia | 79.27 | Fur cow | 0.69 |  |
|  |  |  |  | Fur dog | 78.99 |  |  |  |
|  |  |  |  | Fur cat european shorthair | 78.97 | Fur wildboar | 0.69 |  |
|  |  |  |  | Fur red deer | 78.91 |  |  |  |
| LC-S | C3b | Fiber | Blue | Mask 128S Ge ATR #2 | 97.78 | Cardboard/cellulose | 0.98 | Anthropogenic origin |
|  |  |  |  | Cotton Blue fiber | 97.24 |  |  |  |
|  |  |  |  | Cotton Yellow Fiber | 97.10 | Papercup cellulosic | 0.97 |  |
|  |  |  |  | Cotton Black Fiber | 96.71 |  |  |  |
|  |  |  |  | Cotton Brown Fiber | 96.16 | Cellulose | 0.95 |  |
|  |  |  |  | Cotton Blue fiber | 94.21 |  |  |  |
|  |  |  |  | Cotton Blue fiber | 94.20 | Cellulose | 0.94 |  |
|  |  |  |  | Cotton Green Fiber | 93.91 |  |  |  |
|  |  |  |  | Cotton Purple Fiber | 93.57 | Fibre poplar down | 0.93 |  |
|  |  |  |  | Cotton Pink fiber Bundle | 91.77 |  |  |  |
| C-V | 7a | Fiber | White | Cotton green fiber | 90.82 | Cardboard/Cellulose | 0.97 | Anthropogenic origin |
|  |  |  |  | Mask 128S GeATR #2 | 90.34 |  |  |  |
|  |  |  |  | Cotton yellow fiber | 89.43 | Papercup cellulosic | 0.96 |  |
|  |  |  |  | Cotton purple fiber | 88.87 |  |  |  |
|  |  |  |  | Cotton blue fiber | 88.80 | Cellulose | 0.91 |  |
|  |  |  |  | Cotton grey fiber | 87.96 |  |  |  |
|  |  |  |  | Cotton yellow fiber | 87.84 | Cellulose | 0.91 |  |
|  |  |  |  | Cotton brown fiber | 87.55 |  |  |  |
|  |  |  |  | Cotton pink fiber | 86.47 | Cellulose | 0.90 |  |
|  |  |  |  | Cotton grey fiber | 86.31 |  |  |  |
|  | 7b | Fiber | Clear | Fibre grass | 82.06 | Cardboard/Cellulose | 0.96 | Anthropogenic origin |
|  |  |  |  | Cotton green fiber | 81.73 |  |  |  |
|  |  |  |  | Cotton grey fiber | 81.06 | Papercup cellulosic | 0.95 |  |
|  |  |  |  | Cotton yellow fiber | 80.91 |  |  |  |
|  |  |  |  | Cotton grey fiber | 80.10 | Methyl Cellulose | 0.90 |  |
|  |  |  |  | Cotton pink fiber | 79.84 |  |  |  |
|  |  |  |  | Cotton purple fiber | 79.36 | Fibre poplar down | 0.90 |  |
|  |  |  |  | Wood mahagoni | 78.59 |  |  |  |
|  |  |  |  | Mask 128S GeATR #2 | 78.49 | Fibre poplar down | 0.90 |  |
|  |  |  |  | Cellulose wipe | 78.28 |  |  |  |
|  | 8a | Fiber | Black | Cotton blue fiber | 97.53 | Cardboard/Cellulose | 0.97 | Anthropogenic origin |
|  |  |  |  | Cotton black fiber | 97.23 |  |  |  |
|  |  |  |  | Cotton brown fiber | 97.13 | Papercup cellulosic | 0.97 |  |
|  |  |  |  | Cotton yellow fiber | 97.12 |  |  |  |
|  |  |  |  | Mask 128S GeATR #2 | 96.13 | Cellulose | 0.93 |  |
|  |  |  |  | Cotton blue fiber | 94.85 |  |  |  |
|  |  |  |  | Cotton pink fiber bundle | 94.64 | Cellulose | 0.93 |  |
|  |  |  |  | Cotton blue fiber | 93.89 |  |  |  |
|  |  |  |  | Cotton purple fiber | 93.83 | .Cellulose wipe | 0.92 |  |
|  |  |  |  | Cotton green fiber | 93.52 |  |  |  |
|  | 8b | Fiber | Blue | Cotton yellow fiber | 95.52 | Cardboard/Cellulose | 0.98 | Anthropogenic origin |
|  |  |  |  | Cotton yellow fiber | 94.54 |  |  |  |
|  |  |  |  | Cotton black fiber | 93.48 | Papercup cellulosic | 0.97 |  |
|  |  |  |  | Cotton blue fiber | 93.41 |  |  |  |
|  |  |  |  | Cotton green fiber | 93.36 | Methyl Cellulose | 0.92 |  |
|  |  |  |  | Cotton white lab coat fiber | 93.29 |  |  |  |
|  |  |  |  | Cotton purple fiber | 92.51 | Cellulose | 0.92 |  |
|  |  |  |  | Cotton brown fiber | 92.30 |  |  |  |
|  |  |  |  | Mask 128S GeATR #2 | 92.21 | Hydroxyethyl cellulose | 0.92 |  |
|  |  |  |  | Cotton grey fiber | 91.42 |  |  |  |
|  | 9a | Fiber | Black | Cotton black fiber | 96.69 | Cardboard/Cellulose | 0.97 | Anthropogenic origin |
|  |  |  |  | Cotton yellow fiber | 95.86 |  |  |  |
|  |  |  |  | Mask 128S GeATR #2 | 95.27 | Papercup cellulosic | 0.96 |  |
|  |  |  |  | Cotton blue fiber | 95.07 |  |  |  |
|  |  |  |  | Cotton brown fiber | 93.89 | Cellulose | 0.93 |  |
|  |  |  |  | Cotton green fiber | 93.40 |  |  |  |
|  |  |  |  | Cotton pink fiber bundle | 92.37 | Cellulose | 0.92 |  |
|  |  |  |  | Cotton blue fiber | 92.05 |  |  |  |
|  |  |  |  | Cotton blue fiber | 91.00 | Fibre poplar down | 0.90 |  |
|  |  |  |  | Cotton white lab coat fiber | 90.34 |  |  |  |
|  | 10a | Fiber | Clear | Cotton green fiber | 78.09 | Cardboard/Cellulose | 0.96 | Anthropogenic origin |
|  |  |  |  | Cotton yellow fiber | 76.17 |  |  |  |
|  |  |  |  | Cotton grey fiber | 75.77 | Papercup cellulosic | 0.95 |  |
|  |  |  |  | Cotton white lab coat fiber | 75.69 |  |  |  |
|  |  |  |  | Cotton pink fiber | 74.30 | Cellulose | 0.93 |  |
|  |  |  |  | Cellulose | 72.97 |  |  |  |
|  |  |  |  | Cotton purple fiber | 72.72 | Cellulose | 0.93 |  |
|  |  |  |  | Mask 128S GeATR #2 | 72.67 |  |  |  |
|  |  |  |  | Cotton yellow fiber | 72.19 | .Cellulose wipe | 0.92 |  |
|  |  |  |  | Cotton blue fiber | 71.19 |  |  |  |
| H | 14a | Fiber | Clear | Cotton pink fiber | 80.27 | Cardboard/Cellulose | 0.98 | Anthropogenic origin |
|  |  |  |  | Cotton grey fiber | 77.89 |  |  |  |
|  |  |  |  | Cotton yellow fiber | 75.16 | Papercup cellulosic | 0.97 |  |
|  |  |  |  | Cotton green fiber | 74.42 |  |  |  |
|  |  |  |  | Fibre grass | 73.94 | Methyl Cellulose | 0.93 |  |
|  |  |  |  | Cotton red fabric | 73.87 |  |  |  |
|  |  |  |  | Cotton grey fiber | 72.47 | Hydroxyethyl cellulose | 0.92 |  |
|  |  |  |  | Fibre linen | 68.96 |  |  |  |
|  |  |  |  | Cellulose | 68.68 | Leaf plant | 0.92 |  |
|  |  |  |  | Cotton purple fiber | 68.52 |  |  |  |
|  | 14b | Fiber | Clear | Cotton black fiber | 93.82 | Cardboard/Cellulose | 0.96 | Anthropogenic origin |
|  |  |  |  | Cotton pink fiber bundle | 93.35 |  |  |  |
|  |  |  |  | Cotton yellow fiber | 92.82 | Papercup cellulosic | 0.95 |  |
|  |  |  |  | Cotton brown fiber | 92.81 |  |  |  |
|  |  |  |  | Cotton blue fiber | 92.74 | Cellulose | 0.90 |  |
|  |  |  |  | Mask 128S GeATR #2 | 91.42 |  |  |  |
|  |  |  |  | Cotton blue fiber | 90.09 | Methyl Cellulose | 0.90 |  |
|  |  |  |  | Cotton purple fiber | 89.79 |  |  |  |
|  |  |  |  | Cotton green fiber | 89.66 | Cellulose | 0.90 |  |
|  |  |  |  | Cotton blue fiber | 89.44 |  |  |  |
| L-C-V | C1a | Fiber | Clear | Mask 128S GeATR #2 | 91.38 | Cardboard/Cellulose | 0.98 | Anthropogenic origin |
|  |  |  |  | Cotton green fiber | 90.69 |  |  |  |
|  |  |  |  | Cotton yellow fiber | 89.55 | Papercup cellulosic | 0.97 |  |
|  |  |  |  | Cotton yellow fiber | 89.42 |  |  |  |
|  |  |  |  | Cotton purple fiber | 89.20 | Methyl Cellulose | 0.92 |  |
|  |  |  |  | Cotton blue fiber | 89.20 |  |  |  |
|  |  |  |  | Cellulose wipe | 88.94 | Hydroxyethyl cellulose | 0.91 |  |
|  |  |  |  | Cellulose | 88.72 |  |  |  |
|  |  |  |  | Cotton blue fiber | 88.40 | Cellulose | 0.91 |  |
|  |  |  |  | Cotton grey fiber | 88.33 |  |  |  |
|  | C1b | Fiber | Clear | Mask 128S GeATR #2 | 93.92 | Cardboard/Cellulose | 0.97 | Anthropogenic origin |
|  |  |  |  | Cotton blue fiber | 92.89 |  |  |  |
|  |  |  |  | Cotton yellow fiber | 92.88 | Papercup cellulosic | 0.96 |  |
|  |  |  |  | Cotton black fiber | 92.53 |  |  |  |
|  |  |  |  | Cotton blue fiber | 91.38 | Methyl Cellulose | 0.91 |  |
|  |  |  |  | Cotton brown fiber | 91.08 |  |  |  |
|  |  |  |  | Cotton purple fiber | 90.78 | Cellulose | 0.91 |  |
|  |  |  |  | Cotton yellow fiber | 90.09 |  |  |  |
|  |  |  |  | Cotton green fiber | 89.33 | Hydroxyethyl cellulose | 0.90 |  |
|  |  |  |  | Cotton white lab coat fiber | 89.26 |  |  |  |
|  | C3a | Fiber | White | Cotton brown fiber | 95.18 | Cardboard/Cellulose | 0.96 | Anthropogenic origin |
|  |  |  |  | Cotton blue fiber | 93.68 |  |  |  |
|  |  |  |  | Cotton purple fiber | 93.07 | Papercup cellulosic | 0.95 |  |
|  |  |  |  | Mask 128S GeATR #2 | 82.51 |  |  |  |
|  |  |  |  | Cotton blue fiber | 81.41 | Cellulose | 0.91 |  |
|  |  |  |  | Cotton yellow fiber | 81.17 |  |  |  |
|  |  |  |  | Fibre cotton combers | 90.47 | .Cellulose wipe | 0.91 |  |
|  |  |  |  | Cotton black fiber | 90.41 |  |  |  |
|  |  |  |  | Cotton blue fiber | 90.09 | Cellulose | 0.90 |  |
|  |  |  |  | Cellulose acetate filter | 90.05 |  |  |  |
|  | C3b | Fiber | Yellow | Mask 128S GeATR #2 | 90.34 | Cardboard/Cellulose | 0.97 | Anthropogenic origin |
|  |  |  |  | Cotton yellow fiber | 90.22 |  |  |  |
|  |  |  |  | Cotton green fiber | 90.12 | Papercup cellulosic | 0.96 |  |
|  |  |  |  | Cotton blue fiber | 89.96 |  |  |  |
|  |  |  |  | Cotton yellow fiber | 89.48 | Cellulose | 0.91 |  |
|  |  |  |  | Cotton purple fiber | 89.12 |  |  |  |
|  |  |  |  | Cotton black fiber | 88.71 | Cellulose | 0.90 |  |
|  |  |  |  | Cotton grey fiber | 88.50 |  |  |  |
|  |  |  |  | Cotton blue fiber | 88.36 | Fibre poplar down | 0.88 |  |
|  |  |  |  | Cotton brown fiber | 88.01 |  |  |  |
|  | 8a | Fiber | Blue | Cotton black fiber | 92.45 | Cardboard/Cellulose | 0.92 | Anthropogenic origin |
|  |  |  |  | Cotton blue fiber | 91.69 |  |  |  |
|  |  |  |  | Cotton pink fiber bundle | 91.55 | Papercup cellulosic | 0.92 |  |
|  |  |  |  | Cotton yellow fiber | 91.28 |  |  |  |
|  |  |  |  | Cotton brown fiber | 90.89 | Methyl Cellulose | 0.90 |  |
|  |  |  |  | Cotton blue fiber | 90.16 |  |  |  |
|  |  |  |  | Mask 128S GeATR #2 | 88.77 | Hydroxyethyl cellulose | 0.90 |  |
|  |  |  |  | Cotton blue fiber | 88.50 |  |  |  |
|  |  |  |  | Cotton green fiber | 87.60 | Hydroxyethyl cellulose | 0.90 |  |
|  |  |  |  | Cotton purple fiber | 87.34 |  |  |  |
| L-C-S | 6a | Fiber | Black | Polyester white label fiber | 96.81 | Polyesterterphthalate | 0.98 | Synthetic |
|  |  |  |  | PET white fragment | 96.68 |  |  |  |
|  |  |  |  | PET yellow fiber bundle | 96.60 | Polyethylene terephthalate | 0.97 |  |
|  |  |  |  | PET white fiber bundle | 96.38 |  |  |  |
|  |  |  |  | Polyester brown yarn fiber | 96.07 | Polyethylene terephthalate | 0.96 |  |
|  |  |  |  | Polyester blue yarn fiber | 95.79 |  |  |  |
|  |  |  |  | Polyester white fiber | 95.40 | Polyethylene terephthalate | 0.96 |  |
|  |  |  |  | PET orange fiber | 95.35 |  |  |  |
|  |  |  |  | Polyester green label fiber | 95.27 | PET | 0.96 |  |
|  |  |  |  | PET Blue fragment | 95.16 |  |  |  |
|  | 6b | Fiber | Yellow | Cotton brown fiber | 95.91 | Cardboard/Cellulose | 0.97 | Anthropogenic origin |
|  |  |  |  | Cotton blue fiber | 94.82 |  |  |  |
|  |  |  |  | Cotton blue fiber | 93.41 | Papercup cellulosic | 0.96 |  |
|  |  |  |  | Cotton purple fiber | 93.17 |  |  |  |
|  |  |  |  | Cotton yellow fiber | 92.83 | Cellulose | 0.93 |  |
|  |  |  |  | Mask 128S GeATR #2 | 92.41 |  |  |  |
|  |  |  |  | Cotton black fiber | 92.39 | Cellulose | 92.00 |  |
|  |  |  |  | Cotton blue fiber | 91.49 |  |  |  |
|  |  |  |  | Cotton pink fiber bundle | 91.02 | Fibre poplar down | 0.91 |  |
|  |  |  |  | Cotton yellow fiber | 90.90 |  |  |  |
|  | 19a | Fiber | Clear |  |  | Cardboard/Cellulose | 0.97 |  |
|  |  |  |  |  |  |  |  |  |
|  |  |  |  |  |  | Papercup cellulosic | 0.96 |  |
|  |  |  |  |  |  |  |  |  |
|  |  |  |  |  |  | Fibre viscose dyed | 0.92 |  |
|  |  |  |  |  |  |  |  |  |
|  |  |  |  |  |  | .Cellulose wipe | 0.92 |  |
|  |  |  |  |  |  |  |  |  |
|  |  |  |  |  |  | Cellulose | 0.92 |  |
|  |  |  |  |  |  |  |  |  |
|  | 19b | Fiber | Blue | Cotton brown fiber | 97.26 | Cardboard/Cellulose | 0.97 | Anthropogenic origin |
|  |  |  |  | Cotton blue fiber | 96.45 |  |  |  |
|  |  |  |  | Cotton yellow fiber | 94.52 | Papercup cellulosic | 0.96 |  |
|  |  |  |  | Mask 128S GeATR #2 | 94.51 |  |  |  |
|  |  |  |  | Cotton black fiber | 94.40 | Cellulose | 0.93 |  |
|  |  |  |  | Cotton blue fiber | 94.31 |  |  |  |
|  |  |  |  | Cotton purple fiber | 94.00 | Cellulose | 0.93 |  |
|  |  |  |  | Cotton pink fiber bundle | 92.88 |  |  |  |
|  |  |  |  | Cotton blue fiber | 92.51 | .Cellulose wipe | 0.92 |  |
|  |  |  |  | Cotton green fiber | 91.39 |  |  |  |
|  | 22a | Fiber | Black | Polyester red yarn fiber | 96.00 | Polyester | 0.96 | Synthetic |
|  |  |  |  | Polyester white fiber | 94.63 |  |  |  |
|  |  |  |  | PET yellow fiber bundle | 94.41 | Polyester | 0.96 |  |
|  |  |  |  | Polyester pink fiber | 94.03 |  |  |  |
|  |  |  |  | Polyester blue yarn fiber | 93.40 | Polyesterterphthalate | 0.95 |  |
|  |  |  |  | Polyester brown yarn fiber | 93.29 |  |  |  |
|  |  |  |  | Polyester green label fiber | 93.25 | Polyethylene terephthalate | 0.95 |  |
|  |  |  |  | Polyester white fabric bag fiber | 93.24 |  |  |  |
|  |  |  |  | Polyester white label fiber | 93.00 | Polyesterterphthalate | 0.95 |  |
|  |  |  |  | Polyester white fiber | 92.95 |  |  |  |
|  | 22b | Fiber | Yellow | PET white fragment | 95.23 | Polyethylene terephthalate | 0.94 | Synthetic |
|  |  |  |  | PET white fiber bundle | 94.65 |  |  |  |
|  |  |  |  | PET white fiber bundle | 94.63 | Polyesterterphthalate | 0.94 |  |
|  |  |  |  | Polyester white label fiber | 94.61 |  |  |  |
|  |  |  |  | PET blue fiber bundle | 94.36 | PET | 0.93 |  |
|  |  |  |  | Polyester brown yarn fiber | 93.89 |  |  |  |
|  |  |  |  | PET orange fiber | 93.59 | PET | 0.93 |  |
|  |  |  |  | PET Blue fragment | 93.25 |  |  |  |
|  |  |  |  | PET yellow fiber bundle | 93.23 | PET | 0.93 |  |
|  |  |  |  | PET purple fiber bundle | 92.69 |  |  |  |
| BR-S | 6a | Fiber | Black | PET yellow fiber bundle | 97.64 | Polyesterterphthalate | 0.98 | Synthetic |
|  |  |  |  | Polyester white label fiber | 97.39 |  |  |  |
|  |  |  |  | Polyester blue yarn fiber | 97.37 | Polyethylene terephthalate | 0.97 |  |
|  |  |  |  | PET white fragment | 97.31 |  |  |  |
|  |  |  |  | Polyester green label fiber | 96.81 | Polyethylene terephthalate | 0.97 |  |
|  |  |  |  | Polyester dark brown yarn fiber | 96.43 |  |  |  |
|  |  |  |  | Polyester red fiber | 96.37 | Polyethylene terephthalate | 0.97 |  |
|  |  |  |  | Polyester brown yarn fiber | 96.26 |  |  |  |
|  |  |  |  | PET Black fiber | 96.23 | Polyethylene terephthalate | 0.96 |  |
|  |  |  |  | Polyester red yarn fiber | 96.21 |  |  |  |
|  | 6b | Fiber | Clear | Cotton pink fiber | 91.53 | Cardboard/Cellulose | 0.97 | Anthropogenic origin |
|  |  |  |  | Cotton green fiber | 89.23 |  |  |  |
|  |  |  |  | Cotton yellow fiber | 87.30 | Papercup cellulosic | 0.96 |  |
|  |  |  |  | Cotton grey fiber | 87.29 |  |  |  |
|  |  |  |  | Cellulose | 84.62 | Cellulose | 0.93 |  |
|  |  |  |  | Cotton grey fiber | 81.80 |  |  |  |
|  |  |  |  | Cellophane | 81.65 | Cellulose | 0.92 |  |
|  |  |  |  | Cotton purple fiber | 79.44 |  |  |  |
|  |  |  |  | Fibre viscose | 79.02 | Fibre poplar down | 0.91 |  |
|  |  |  |  | Cotton white lab coat fiber | 78.54 |  |  |  |
|  | 23a | Fiber | Black | Cotton yellow fiber | 87.98 | Cardboard/Cellulose | 0.98 | Anthropogenic origin |
|  |  |  |  | Cotton green fiber | 87.14 |  |  |  |
|  |  |  |  | Cotton pink fiber | 87.13 | Papercup cellulosic | 0.97 |  |
|  |  |  |  | Cotton grey fiber | 86.33 |  |  |  |
|  |  |  |  | Cotton white lab coat fiber | 84.46 | Methyl Cellulose | 0.93 |  |
|  |  |  |  | Cotton grey fiber | 81.58 |  |  |  |
|  |  |  |  | Cellophane | 80.90 | Hydroxyethyl cellulose | 0.92 |  |
|  |  |  |  | Cotton purple fiber | 79.11 |  |  |  |
|  |  |  |  | Cotton yellow fiber | 79.01 | Hydroxypropyl methyl cellulose | 0.91 |  |
|  |  |  |  | Mask 128S GeATR #2 | 77.81 |  |  |  |
|  | 24a | Fiber | Black | PET orange foam | 90.73 | Polyethylene terephthalate | 0.89 | Synthetic |
|  |  |  |  | PET white fiber bundle | 90.29 |  |  |  |
|  |  |  |  | Polyester red yarn fiber | 90.07 | Polyethylene terephthalate | 0.89 |  |
|  |  |  |  | PET Blue fragment | 88.30 |  |  |  |
|  |  |  |  | PET white fragment | 88.05 | Polyethylene terephthalate | 0.89 |  |
|  |  |  |  | Polyester green label fiber | 87.77 |  |  |  |
|  |  |  |  | Polyester white fabric bag fiber | 87.30 | Polyesterterphthalate | 0.88 |  |
|  |  |  |  | PET yellow fiber bundle | 87.12 |  |  |  |
|  |  |  |  | PET clear fragment | 86.98 | Polyethylene terephthalate | 0.87 |  |
|  |  |  |  | PET white fiber bundle | 86.96 |  |  |  |
|  | 24b | Fiber | Black | PET blue fiber bundle | 95.00 | Polyethylene terephthalate | 0.97 | Synthetic |
|  |  |  |  | Polyethylene terephthalate | 94.76 |  |  |  |
|  |  |  |  | PET white fragment | 94.54 | Polyethylene terephthalate | 0.96 |  |
|  |  |  |  | Polyester red fiber | 94.50 |  |  |  |
|  |  |  |  | Polyester | 94.08 | Polyethylene terephthalate | 0.95 |  |
|  |  |  |  | Polyethylene terephthalate | 94.01 |  |  |  |
|  |  |  |  | PET yellow fiber bundle | 93.81 | Polyethylene terephthalate | 0.95 |  |
|  |  |  |  | Polyester blue yarn fiber | 93.80 |  |  |  |
|  |  |  |  | Polyester white label fiber | 93.76 | Polyesterterphthalate | 0.95 |  |
|  |  |  |  | PET Black fiber | 93.56 |  |  |  |
| LOP | 7c | Fragment | clear | Polyethylene low density | 91.86 | Polyethylene | 0.94 | Synthetic |
|  |  |  |  | PE white film | 91.48 |  |  |  |
|  |  |  |  | ethylene propylene | 91.47 | Ethylene ethyl acrylate | 0.94 |  |
|  |  |  |  | Polyethylene low density | 91.25 |  |  |  |
|  |  |  |  | PE grev pellet | 91.12 | Polyethylene | 0.93 |  |
|  |  |  |  | PEVA oink flip flop foam | 91.01 |  |  |  |
|  |  |  |  | PE green tampon wrapper film | 91.01 | Honeycomb freshly removed with nectar | 0.93 |  |
|  |  |  |  | fibre thermoplastic elastomere | 90.95 |  |  |  |
|  |  |  |  | PE orange foam | 90.73 | Honeycomb freshly removed | 0.93 |  |
|  |  |  |  | PE white film | 90.63 |  |  |  |
| C | Test | Film | Brown | Cotton black fiber | 88.02 | Cardboard/Cellulose | 0.97 | Anthropogenic origin |
|  |  |  |  | Cotton yellow fiber | 87.81 |  |  |  |
|  |  |  |  | Mask 128S GeATR #2 | 87.48 | Papercup cellulosic | 0.96 |  |
|  |  |  |  | Cotton blue fiber | 87.04 |  |  |  |
|  |  |  |  | Cotton blue fiber | 86.82 | Methyl Cellulose | 0.91 |  |
|  |  |  |  | Cotton white lab coat fiber | 84.98 |  |  |  |
|  |  |  |  | Cotton brown fiber | 84.88 | Hydroxyethyl cellulose | 0.90 |  |
|  |  |  |  | Cotton purple fiber | 84.09 |  |  |  |
|  |  |  |  | Cotton green fiber | 84.09 | Cellulose | 0.90 |  |
|  |  |  |  | Cotton black fiber | 83.88 |  |  |  |
| PS-V | 4a | Fiber | Clear | Chitin from crustacean shells | 87.52 | Cardboard/ Cellulose | 0.93 | Natural |
|  |  |  |  | Cellophane | 53.29 |  |  |  |
|  |  |  |  | Methyl cellulose | 46.67 | Papercup cellulosic | 0.93 |  |
|  |  |  |  | Cellophane | 46.36 |  |  |  |
|  |  |  |  | Cotton grey fiber | 42.65 | Hydroxyethyl cellulose | 0.88 |  |
|  |  |  |  | Algae fucus serratus | 41.99 |  |  |  |
|  |  |  |  | Cotton grey fiber | 41.63 | Chitin from crustacean shells | 0.88 |  |
|  |  |  |  | Hydroxyethyl beta cyclodextrin | 41.30 |  |  |  |
|  |  |  |  | Cotton green fiber | 41.27 | Methyl cellulose | 0.87 |  |
|  |  |  |  | Methylene propanediol | 41.11 |  |  |  |
| PS-V | 4b | Fiber | Clear | Polyester white label fiber | 97.32 | Polyesterterpthalate | 0.95 | Synthetic |
|  |  |  |  | PET white fiber bundle | 97.26 |  |  |  |
|  |  |  |  | PET Yellow fiber bundle | 97.10 | Polyethylene terephthalate | 0.95 |  |
|  |  |  |  | Polyester green label fiber | 97.10 |  |  |  |
|  |  |  |  | PET white fragment | 97.04 | Polyethylene terephthalate | 0.95 |  |
|  |  |  |  | Polyester blue yarn fiber | 96.58 |  |  |  |
|  |  |  |  | PET blue fragment | 96.52 | PET | 0.95 |  |
|  |  |  |  | PET orange fiber | 96.34 |  |  |  |
|  |  |  |  | Polyester white fiber | 96.30 | Polyethylene terephthalate | 0.94 |  |
|  |  |  |  | Polyester dark brown yarn fiber | 95.92 |  |  |  |
| PS-V | 6a | Fiber | Clear | Cotton brown fiber | 98.80 | Cardboard/ Cellulose | 0.97 | Anthropogenically impacted |
|  |  |  |  | Cotton blue fiber | 98.31 |  |  |  |
|  |  |  |  | Cotton purple fiber | 97.00 | Papercup cellulosic | 0.96 |  |
|  |  |  |  | Mask 128S GeATR #2 | 96.47 |  |  |  |
|  |  |  |  | Cotton blue fiber | 96.38 | Cellulose | 0.93 |  |
|  |  |  |  | Cotton Yellow Fiber | 95.90 |  |  |  |
|  |  |  |  | Cotton blue fiber | 94.74 | Cellulose | 0.93 |  |
|  |  |  |  | Cotton black fiber | 94.50 |  |  |  |
|  |  |  |  | Cotton Yellow Fiber | 94.23 | .Cellulose wipe | 0.91 |  |
|  |  |  |  | Cotton green fiber | 93.53 |  |  |  |
| PS-V | 6b | Fiber | White | Cotton brown fiber | 97.94 | Cardboard/ Cellulose | 0.98 | Anthropogenically impacted |
|  |  |  |  | Cotton blue fiber | 97.77 |  |  |  |
|  |  |  |  | Mask 128S GeATR #2 | 97.62 | Papercup cellulosic | 0.97 |  |
|  |  |  |  | Cotton purple fiber | 96.13 |  |  |  |
|  |  |  |  | Cotton Yellow Fiber | 95.98 | Cellulose | 0.93 |  |
|  |  |  |  | Cotton blue fiber | 95.06 |  |  |  |
|  |  |  |  | Cotton black fiber | 94.84 | Cellulose | 0.93 |  |
|  |  |  |  | Cotton blue fiber | 93.64 |  |  |  |
|  |  |  |  | Cotton green fiber | 93.23 | .Cellulose wipe | 0.91 |  |
|  |  |  |  | Cotton Yellow Fiber | 93.15 |  |  |  |
| PS-V | 7a | Fiber | Clear | Cotton green fiber | 95.28 | Cardboard/ Cellulose | 0.97 | Anthropogenically impacted |
|  |  |  |  | Cotton pink fiber | 94.39 |  |  |  |
|  |  |  |  | Cotton Yellow Fiber | 93.26 | Papercup cellulosic | 0.96 |  |
|  |  |  |  | Cotton grey fiber | 92.95 |  |  |  |
|  |  |  |  | Cotton purple fiber | 90.83 | Cellulose | 0.91 |  |
|  |  |  |  | Cotton grey fiber | 87.69 |  |  |  |
|  |  |  |  | Mask 128S GeATR #2 | 87.20 | Cellulose | 0.91 |  |
|  |  |  |  | Cotton grey fiber | 86.55 |  |  |  |
|  |  |  |  | Cotton blue fiber | 86.38 | .Cellulose wipe | 0.90 |  |
|  |  |  |  | Cellulose | 86.35 |  |  |  |
| PS-V | 7b | Fiber | White | Cotton blue fiber | 96.18 | Cardboard/ Cellulose | 0.96 | Anthropogenically impacted |
|  |  |  |  | Cotton brown fiber | 96.05 |  |  |  |
|  |  |  |  | Cotton pink fiber bundle | 95.79 | Papercup cellulosic | 0.95 |  |
|  |  |  |  | Cotton black fiber | 95.65 |  |  |  |
|  |  |  |  | Cotton blue fiber | 94.96 | Cellulose | 0.90 |  |
|  |  |  |  | Cotton Yellow Fiber | 94.83 |  |  |  |
|  |  |  |  | Cotton purple fiber | 93.49 | Methyl cellulose | 0.90 |  |
|  |  |  |  | Cotton blue fiber | 93.10 |  |  |  |
|  |  |  |  | Mask 128S GeATR #2 | 91.98 | Hydroxyethyl cellulose | 0.90 |  |
|  |  |  |  | Cotton Yellow Fiber | 91.54 |  |  |  |
| PS-V | 8a | Fiber | Clear | Cotton brown fiber | 97.60 | Cardboard/ Cellulose | 0.96 | Anthropogenically impacted |
|  |  |  |  | Cotton blue fiber | 97.02 |  |  |  |
|  |  |  |  | Cotton purple fiber | 95.52 | Papercup cellulosic | 0.95 |  |
|  |  |  |  | Mask 128S GeATR #2 | 94.74 |  |  |  |
|  |  |  |  | Cotton Yellow Fiber | 94.66 | .Cellulose wipe | 0.92 |  |
|  |  |  |  | Cotton blue fiber | 94.38 |  |  |  |
|  |  |  |  | Cotton black fiber | 94.12 | Cellulose | 0.91 |  |
|  |  |  |  | Cellulose acetate filter | 93.56 |  |  |  |
|  |  |  |  | Cotton blue fiber | 93.27 | Fibre cotton combers | 0.91 |  |
|  |  |  |  | Fibre cotton combers | 92.99 |  |  |  |
| PS-V | 8b | Fiber | Clear | Chitin from crustacean shells | 86.60 | Fur yak bleached | 0.74 | Keratin based |
|  |  |  |  | Cellophane | 50.31 |  |  |  |
|  |  |  |  | Algae fucus serratus | 49.21 | Fur wild boar | 0.72 |  |
|  |  |  |  | Fur red deer | 47.83 |  |  |  |
|  |  |  |  | Merino scoured wool made rough | 47.56 | Fur red deer | 0.71 |  |
|  |  |  |  | wool cashmere kasakhstan | 47.56 |  |  |  |
|  |  |  |  | Wool raw cashmere afghanistan | 47.36 | Fur dog | 0.68 |  |
|  |  |  |  | Wool raw cashmere mongolia | 47.34 |  |  |  |
|  |  |  |  | Wool slubbing rough | 47.32 | Fur cow | 0.67 |  |
|  |  |  |  | Fur wild boar | 47.32 |  |  |  |
| PS-V | 10a | Fiber | Clear | Mask 128S GeATR #2 | 97.73 | Cardboard/ Cellulose | 0.98 | Anthropogenically impacted |
|  |  |  |  | Cotton blue fiber | 97.01 |  |  |  |
|  |  |  |  | Cotton brown fiber | 96.68 | Papercup cellulosic | 0.97 |  |
|  |  |  |  | Cotton Yellow Fiber | 96.22 |  |  |  |
|  |  |  |  | Cotton black fiber | 95.08 | Cellulose | 0.94 |  |
|  |  |  |  | Cotton purple fiber | 94.87 |  |  |  |
|  |  |  |  | Cotton blue fiber | 94.04 | Cellulose | 0.93 |  |
|  |  |  |  | Cotton blue fiber | 93.18 |  |  |  |
|  |  |  |  | Cotton green fiber | 93.14 | Hydroxyethyl cellulose | 0.93 |  |
|  |  |  |  | Cotton Yellow Fiber | 92.79 |  |  |  |
| PS-V | 10b | Fiber | Clear | Cotton Yellow Fiber | 86.10 | Cardboard/ Cellulose | 0.97 | Anthropogenically impacted |
|  |  |  |  | Cotton green fiber | 85.76 |  |  |  |
|  |  |  |  | Cotton blue fiber | 85.57 | Papercup cellulosic | 0.96 |  |
|  |  |  |  | Mask 128S GeATR #2 | 85.22 |  |  |  |
|  |  |  |  | Cotton blue fiber | 85.01 | Methyl cellulose | 0.91 |  |
|  |  |  |  | Cotton purple fiber | 84.75 |  |  |  |
|  |  |  |  | Wood mahagoni | 84.29 | Fibre poplar down | 0.90 |  |
|  |  |  |  | Cotton Yellow Fiber | 84.28 |  |  |  |
|  |  |  |  | Wood pine | 84.17 | Hydroxyethyl cellulose | 0.90 |  |
|  |  |  |  | Cotton brown fiber | 83.78 |  |  |  |
| PS-V | 12a | Fiber | Yellow | Wood pine | 84.85 | Cardboard/ Cellulose | 0.97 | Anthropogenically impacted |
|  |  |  |  | Wood mahagoni | 83.67 |  |  |  |
|  |  |  |  | Cotton green fiber | 78.59 | Papercup cellulosic | 0.96 |  |
|  |  |  |  | Fibre turf | 78.10 |  |  |  |
|  |  |  |  | Cotton Yellow Fiber | 77.63 | Methyl cellulose | 0.91 |  |
|  |  |  |  | Cotton grey fiber | 76.63 |  |  |  |
|  |  |  |  | Fibre poplar down | 76.42 | Hydroxyethyl cellulose | 0.91 |  |
|  |  |  |  | Cotton pink fiber | 75.60 |  |  |  |
|  |  |  |  | Fibre poplar down | 75.11 | Cellulose | 0.90 |  |
|  |  |  |  | Cotton purple fiber | 74.32 |  |  |  |
| PS-V | 12b | Fiber | Clear | Cotton brown fiber | 98.67 | Cardboard/ Cellulose | 0.97 | Anthropogenically impacted |
|  |  |  |  | Cotton blue fiber | 98.26 |  |  |  |
|  |  |  |  | Mask 128S GeATR #2 | 96.86 | Papercup cellulosic | 0.96 |  |
|  |  |  |  | Cotton blue fiber | 96.18 |  |  |  |
|  |  |  |  | Cotton Yellow Fiber | 96.05 | Cellulose | 0.94 |  |
|  |  |  |  | Cotton purple fiber | 95.39 |  |  |  |
|  |  |  |  | Cotton black fiber | 95.35 | Cellulose | 0.94 |  |
|  |  |  |  | Cotton blue fiber | 94.24 |  |  |  |
|  |  |  |  | Cellulose acetate filter | 93.23 | .Cellulose wipe | 0.92 |  |
|  |  |  |  | Cotton green fiber | 93.15 |  |  |  |
| PS-V | 16a | Fiber | Blue | Mask 128S GeATR #2 | 98.18 | Cardboard/ Cellulose | 0.98 | Anthropogenically impacted |
|  |  |  |  | Cotton blue fiber | 97.30 |  |  |  |
|  |  |  |  | Cotton brown fiber | 97.29 | Papercup cellulosic | 0.97 |  |
|  |  |  |  | Cotton Yellow Fiber | 95.61 |  |  |  |
|  |  |  |  | Cotton purple fiber | 95.48 | Cellulose | 0.95 |  |
|  |  |  |  | Cotton blue fiber | 94.95 |  |  |  |
|  |  |  |  | Cotton black fiber | 94.00 | Cellulose | 0.95 |  |
|  |  |  |  | Cotton green fiber | 93.66 |  |  |  |
|  |  |  |  | Cotton blue fiber | 93.65 | .Cellulose wipe | 0.93 |  |
|  |  |  |  | Cotton grey fiber | 93.47 |  |  |  |
| PS-V | 16b | Fiber | Clear | Cotton pink fiber | 92.40 | Cardboard/ Cellulose | 0.98 | Semi-Synthetic |
|  |  |  |  | Cotton green fiber | 90.47 |  |  |  |
|  |  |  |  | Cotton Yellow Fiber | 89.03 | Papercup cellulosic | 0.97 |  |
|  |  |  |  | Cotton grey fiber | 88.02 |  |  |  |
|  |  |  |  | Cellulose | 85.73 | Hydroxyethyl cellulose | 0.92 |  |
|  |  |  |  | Cotton grey fiber | 82.84 |  |  |  |
|  |  |  |  | Cotton purple fiber | 82.73 | Cellulose | 0.92 |  |
|  |  |  |  | Fibre viscose | 82.30 |  |  |  |
|  |  |  |  | Cellophane | 80.75 | Methyl cellulose | 0.92 |  |
|  |  |  |  | Cellophane | 79.80 |  |  |  |
| PS-V | 17a | Fiber | Clear | Cotton brown fiber | 98.34 | Cardboard/ Cellulose | 0.97 | Anthropogenically impacted |
|  |  |  |  | Cotton blue fiber | 97.87 |  |  |  |
|  |  |  |  | Cotton purple fiber | 96.54 | Papercup cellulosic | 0.96 |  |
|  |  |  |  | Mask 128S GeATR #2 | 96.21 |  |  |  |
|  |  |  |  | Cotton blue fiber | 95.88 | Cellulose | 0.93 |  |
|  |  |  |  | Cotton Yellow Fiber | 95.30 |  |  |  |
|  |  |  |  | Cotton blue fiber | 94.31 | Cellulose | 0.93 |  |
|  |  |  |  | Cotton black fiber | 94.13 |  |  |  |
|  |  |  |  | Cotton grey fiber | 92.92 | .Cellulose wipe | 0.92 |  |
|  |  |  |  | Cellulose acetate filter | 92.73 |  |  |  |
| PS-V | 17b | Fiber | Blue | Cellophane | 80.05 | Cardboard/ Cellulose | 0.97 | Semi-Synthetic |
|  |  |  |  | Cotton pink fiber | 76.42 |  |  |  |
|  |  |  |  | Cotton green fiber | 73.05 | Papercup cellulosic | 0.97 |  |
|  |  |  |  | Cellulose | 72.74 |  |  |  |
|  |  |  |  | Cotton Yellow Fiber | 72.04 | Methyl cellulose | 0.93 |  |
|  |  |  |  | Cotton grey fiber | 71.40 |  |  |  |
|  |  |  |  | Fibre viscose | 66.60 | Hydroxyethyl cellulose | 0.92 |  |
|  |  |  |  | Fibre viscose dyed | 65.94 |  |  |  |
|  |  |  |  | Cotton grey fiber | 65.91 | Cellulose | 0.91 |  |
|  |  |  |  | Cotton white lab coat fiber | 64.81 |  |  |  |
| PS-V | 21a | Fiber | Clear | Wood mahagoni | 83.21 | Cardboard/ Cellulose | 0.97 | Natural |
|  |  |  |  | Cotton pink fiber | 81.32 |  |  |  |
|  |  |  |  | Cotton green fiber | 79.91 | Papercup cellulosic | 0.96 |  |
|  |  |  |  | Wood beech | 79.64 |  |  |  |
|  |  |  |  | Fibre grass | 79.25 | Hydroxyethyl cellulose | 0.91 |  |
|  |  |  |  | Wood pine | 78.95 |  |  |  |
|  |  |  |  | Fibre turf | 78.79 | Cellulose | 0.91 |  |
|  |  |  |  | Cotton Yellow Fiber | 78.37 |  |  |  |
|  |  |  |  | Fibre poplar down | 78.31 | Methyl cellulose | 0.91 |  |
|  |  |  |  | Cotton grey fiber | 78.12 |  |  |  |
| PS-V | 21b | Fragment | Clear | Polypropylene | 98.43 | Polypropyelene | 0.95 | Synthetic |
|  |  |  |  | Polypropylene | 98.23 |  |  |  |
|  |  |  |  | Fibre polypropylene | 97.99 | Polypropyelene | 0.95 |  |
|  |  |  |  | Polypropylene | 97.96 |  |  |  |
|  |  |  |  | Polypropylene | 97.70 | Polypropyelene | 0.94 |  |
|  |  |  |  | PP purple fragment | 97.59 |  |  |  |
|  |  |  |  | Polypropylene | 97.36 | Polypropyelene | 0.94 |  |
|  |  |  |  | PP white toothbrush fragment | 97.31 |  |  |  |
|  |  |  |  | Polypropylene | 97.22 | Polypropyelene | 0.94 |  |
|  |  |  |  | PP purple fragment | 97.02 |  |  |  |
| PS-V | 22a | Fiber | Blue | Cotton brown fiber | 98.08 | Cardboard/ Cellulose | 0.97 | Anthropogenically impacted |
|  |  |  |  | Cotton blue fiber | 97.57 |  |  |  |
|  |  |  |  | Cotton purple fiber | 96.00 | Papercup cellulosic | 0.96 |  |
|  |  |  |  | Cotton blue fiber | 95.60 |  |  |  |
|  |  |  |  | Cotton Yellow Fiber | 94.51 | Cellulose | 0.91 |  |
|  |  |  |  | Mask 128S GeATR #2 | 94.17 |  |  |  |
|  |  |  |  | Cotton black fiber | 94.04 | Cellulose | 0.91 |  |
|  |  |  |  | Cotton blue fiber | 93.89 |  |  |  |
|  |  |  |  | Cotton Yellow Fiber | 92.33 | Fibre poplar down | 0.90 |  |
|  |  |  |  | Cotton pink fiber bundle | 92.27 |  |  |  |
| PS-V | 23a | Fiber | Clear | Cotton purple fiber | 96.95 | Cardboard/ Cellulose | 0.97 | Anthropogenically impacted |
|  |  |  |  | Mask 128S GeATR #2 | 96.51 |  |  |  |
|  |  |  |  | Cotton brown fiber | 96.49 | Papercup cellulosic | 0.96 |  |
|  |  |  |  | Cotton blue fiber | 96.17 |  |  |  |
|  |  |  |  | Cellulose acetate filter | 95.52 | .Cellulose wipe | 0.94 |  |
|  |  |  |  | Fibre cotton combers | 94.03 |  |  |  |
|  |  |  |  | Cotton Yellow Fiber | 93.95 | Cellulose | 0.93 |  |
|  |  |  |  | Cotton green fiber | 93.57 |  |  |  |
|  |  |  |  | Cotton Yellow Fiber | 93.40 | Fibre poplar down | 0.93 |  |
|  |  |  |  | Cotton blue fiber | 92.89 |  |  |  |
| PS-V | 23b | Fiber | Blue | Cotton blue fiber | 86.25 | Cardboard/ Cellulose | 0.98 | Anthropogenically impacted |
|  |  |  |  | Cotton brown fiber | 85.59 |  |  |  |
|  |  |  |  | Cotton black fiber | 85.56 | Papercup cellulosic | 0.97 |  |
|  |  |  |  | Cotton Yellow Fiber | 85.52 |  |  |  |
|  |  |  |  | Mask 128S GeATR #2 | 84.83 | Cellulose | 0.93 |  |
|  |  |  |  | Cotton blue fiber | 84.49 |  |  |  |
|  |  |  |  | Cotton purple fiber | 84.26 | Cellulose | 0.93 |  |
|  |  |  |  | Cotton Yellow Fiber | 83.18 |  |  |  |
|  |  |  |  | Cotton pink fiber bundle | 83.02 | Hydroxyethyl cellulose | 0.92 |  |
|  |  |  |  | Cotton blue fiber | 82.79 |  |  |  |
| PS-V | 24a | Fiber | Clear | Cotton pink fiber | 93.50 | Cardboard/ Cellulose | 0.98 | Semi-Synthetic |
|  |  |  |  | Cotton green fiber | 90.27 |  |  |  |
|  |  |  |  | Cotton Yellow Fiber | 88.65 | Papercup cellulosic | 0.97 |  |
|  |  |  |  | Cotton grey fiber | 88.31 |  |  |  |
|  |  |  |  | Cellulose | 87.58 | Cellulose | 0.93 |  |
|  |  |  |  | Fibre viscose | 93.73 |  |  |  |
|  |  |  |  | Cotton grey fiber | 8.13 | Cellulose | 0.93 |  |
|  |  |  |  | Cotton purple fiber | 81.36 |  |  |  |
|  |  |  |  | Cellophane | 79.72 | .Cellulose wipe | 0.92 |  |
|  |  |  |  | Fibre viscose dyed | 79.32 |  |  |  |
| PS-V | 24b | Fiber | Pink | Cotton green fiber | 95.07 | Cardboard/ Cellulose | 0.98 | Anthropogenically impacted |
|  |  |  |  | Cotton pink fiber | 94.97 |  |  |  |
|  |  |  |  | Cotton Yellow Fiber | 94.54 | Papercup cellulosic | 0.97 |  |
|  |  |  |  | Cotton grey fiber | 92.77 |  |  |  |
|  |  |  |  | Cotton purple fiber | 88.48 | Cellulose | 0.94 |  |
|  |  |  |  | Cotton grey fiber | 88.21 |  |  |  |
|  |  |  |  | Cotton white lab coat fiber | 86.59 | Cellulose | 0.93 |  |
|  |  |  |  | Mask 128S GeATR #2 | 85.94 |  |  |  |
|  |  |  |  | Cotton Yellow Fiber | 85.23 | Hydroxyethyl cellulose | 0.93 |  |
|  |  |  |  | Cotton blue fiber | 84.92 |  |  |  |
| PS-V | 25a | Fiber | Clear | Polypropylene | 96.33 | Polypropyelene | 0.82 | Synthetic |
|  |  |  |  | Fibre polypropylene | 96.17 |  |  |  |
|  |  |  |  | Polypropylene | 96.14 | HDPE | 0.82 |  |
|  |  |  |  | PP white toothbrush fragment | 95.78 |  |  |  |
|  |  |  |  | Polypropylene | 95.77 | Polypropyelene | 0.81 |  |
|  |  |  |  | PP purple fragment | 95.69 |  |  |  |
|  |  |  |  | PP orange fragment | 95.65 | Polypropyelene | 0.81 |  |
|  |  |  |  | Polypropylene | 95.63 |  |  |  |
|  |  |  |  | PP clear fragmnet | 95.63 | Fibre polypropylene dyed | 0.81 |  |
|  |  |  |  | Polypropylene | 95.49 |  |  |  |
| PS-V | 25b | Fiber | Grey | Cotton brown fiber | 87.67 | Cellulose | 0.94 | Anthropogenically impacted |
|  |  |  |  | Mask 128S GeATR #2 | 86.72 |  |  |  |
|  |  |  |  | Cotton blue fiber | 86.70 | Cellulose | 0.94 |  |
|  |  |  |  | Cotton Yellow Fiber | 85.95 |  |  |  |
|  |  |  |  | Cotton blue fiber | 85.34 | Fibre cotton combers | 0.93 |  |
|  |  |  |  | Cotton black fiber | 85.32 |  |  |  |
|  |  |  |  | Cotton pink fiber bundle | 84.89 | Fibre cotton US pima | 0.93 |  |
|  |  |  |  | Cotton green fiber | 83.82 |  |  |  |
|  |  |  |  | Cotton purple fiber | 83.77 | Fibre indian raw cotton | 0.93 |  |
|  |  |  |  | Cotton blue fiber | 83.58 |  |  |  |
| PS-V | 26a | Fiber | Clear | Cotton pink fiber | 91.68 | Cardboard/ Cellulose | 0.98 | Anthropogenically impacted |
|  |  |  |  | Cotton green fiber | 89.61 |  |  |  |
|  |  |  |  | Cotton Yellow Fiber | 88.13 | Papercup cellulosic | 0.97 |  |
|  |  |  |  | Cotton grey fiber | 87.84 |  |  |  |
|  |  |  |  | Cotton grey fiber | 84.13 | Cellulose | 0.93 |  |
|  |  |  |  | Cellophane | 83.14 |  |  |  |
|  |  |  |  | Cellulose | 81.04 | Hydroxyethyl cellulose | 0.93 |  |
|  |  |  |  | Cotton purple fiber | 80.77 |  |  |  |
|  |  |  |  | Fibre grass | 80.65 | Methyl cellulose | 0.92 |  |
|  |  |  |  | Cotton white lab coat fiber | 79.51 |  |  |  |
| PS-V | 26b | Fiber | Clear | Cotton green fiber | 97.01 | Cardboard/ Cellulose | 0.98 | Anthropogenically impacted |
|  |  |  |  | Cotton Yellow Fiber | 96.87 |  |  |  |
|  |  |  |  | Cotton pink fiber | 95.64 | Papercup cellulosic | 0.97 |  |
|  |  |  |  | Cotton grey fiber | 94.56 |  |  |  |
|  |  |  |  | Cotton purple fiber | 93.01 | Cellulose | 0.93 |  |
|  |  |  |  | Cotton grey fiber | 90.13 |  |  |  |
|  |  |  |  | Cotton brown fiber | 89.74 | Cellulose | 0.93 |  |
|  |  |  |  | Cotton blue fiber | 89.69 |  |  |  |
|  |  |  |  | Mask 128S GeATR #2 | 89.17 | .Cellulose wipe | 0.92 |  |
|  |  |  |  | Cotton Yellow Fiber | 88.77 |  |  |  |
| PS-V | 30a | Fiber | Clear | Cotton blue fiber | 92.84 | Cardboard/ Cellulose | 0.97 | Anthropogenically impacted |
|  |  |  |  | Cotton Yellow Fiber | 92.29 |  |  |  |
|  |  |  |  | Cotton black fiber | 91.81 | Papercup cellulosic | 0.96 |  |
|  |  |  |  | Cotton brown fiber | 91.44 |  |  |  |
|  |  |  |  | Cotton blue fiber | 91.20 | Cellulose | 0.92 |  |
|  |  |  |  | Cotton Yellow Fiber | 91.01 |  |  |  |
|  |  |  |  | Cotton purple fiber | 90.54 | Cellulose | 0.91 |  |
|  |  |  |  | Cotton blue fiber | 90.44 |  |  |  |
|  |  |  |  | Cotton pink fiber bundle | 90.00 | Hydroxyethyl cellulose | 0.91 |  |
|  |  |  |  | Mask 128S GeATR #2 | 89.99 |  |  |  |
| PS-V | 30b | Fiber | White | Wood pine | 85.82 | Cardboard/ Cellulose | 0.97 | Natural |
|  |  |  |  | Wood mahagoni | 82.42 |  |  |  |
|  |  |  |  | Fibre turf | 78.45 | Papercup cellulosic | 0.97 |  |
|  |  |  |  | Cotton green fiber | 75.88 |  |  |  |
|  |  |  |  | Cotton pink fiber | 74.37 | Methyl cellulose | 0.93 |  |
|  |  |  |  | Cotton grey fiber | 74.30 |  |  |  |
|  |  |  |  | Cotton Yellow Fiber | 74.02 | Hydroxyethyl cellulose | 0.92 |  |
|  |  |  |  | Fibre poplar down | 73.64 |  |  |  |
|  |  |  |  | Fibre grass | 73.11 | Fibre poplar down | 0.91 |  |
|  |  |  |  | Fibre poplar down | 73.58 |  |  |  |
| PS-S | 3a | Fiber | Clear | Cotton brown fiber | 96.61 | Cardboard/ Cellulose | 0.97 | Anthropogenically impacted |
|  |  |  |  | Cotton purple fiber | 96.04 |  |  |  |
|  |  |  |  | Cotton blue fiber | 95.34 | Papercup cellulosic | 0.96 |  |
|  |  |  |  | Mask 128S GeATR #2 | 94.74 |  |  |  |
|  |  |  |  | Cotton green fiber | 93.59 | Cellulose | 0.93 |  |
|  |  |  |  | Cotton grey fiber | 93.53 |  |  |  |
|  |  |  |  | Cotton blue fiber | 93.35 | Cellulose | 0.93 |  |
|  |  |  |  | Cellulose acetate filter | 92.87 |  |  |  |
|  |  |  |  | Cotton Yellow Fiber | 82.33 | .Cellulose wipe | 0.92 |  |
|  |  |  |  | Fibre cotton combers | 82.07 |  |  |  |
| PS-S | 3b | Fiber | Clear | Cotton green fiber | 92.08 | Cardboard/ Cellulose | 0.97 | Anthropogenically impacted |
|  |  |  |  | Cotton Yellow Fiber | 92.02 |  |  |  |
|  |  |  |  | Mask 128S GeATR #2 | 91.50 | Papercup cellulosic | 0.96 |  |
|  |  |  |  | Cotton Yellow Fiber | 91.24 |  |  |  |
|  |  |  |  | Cotton blue fiber | 91.07 | Cellulose | 0.92 |  |
|  |  |  |  | Cotton black fiber | 89.96 |  |  |  |
|  |  |  |  | Cotton white lab coat fiber | 89.30 | Methyl cellulose | 0.91 |  |
|  |  |  |  | Cotton brown fiber | 89.18 |  |  |  |
|  |  |  |  | Cotton purple fiber | 89.03 | Hydroxyethyl cellulose | 0.91 |  |
|  |  |  |  | Cotton blue fiber | 88.80 |  |  |  |
| PS-S | 4a | Fiber | Clear | Cellulose | 88.45 | Cardboard/ Cellulose | 0.97 | Semi-Synthetic |
|  |  |  |  | Cotton pink fiber | 86.82 |  |  |  |
|  |  |  |  | Cotton green fiber | 84.13 | Papercup cellulosic | 0.96 |  |
|  |  |  |  | Cotton grey fiber | 82.69 |  |  |  |
|  |  |  |  | Cotton Yellow Fiber | 81.16 | Methyl cellulose | 0.92 |  |
|  |  |  |  | Cellophane | 81.13 |  |  |  |
|  |  |  |  | Fibre viscose | 80.67 | Hydroxyethyl cellulose | 0.91 |  |
|  |  |  |  | Fibre viscose dyed | 78.08 |  |  |  |
|  |  |  |  | Cotton grey fiber | 78.01 | Cellulose | 0.90 |  |
|  |  |  |  | Fibre grass | 75.05 |  |  |  |
| PS-S | 4b | Fiber | Clear | Cotton pink fiber | 84.42 | Cardboard/ Cellulose | 0.98 | Anthropogenically impacted |
|  |  |  |  | Cotton green fiber | 80.62 |  |  |  |
|  |  |  |  | Cotton Yellow Fiber | 78.67 | Papercup cellulosic | 0.97 |  |
|  |  |  |  | Cotton grey fiber | 77.79 |  |  |  |
|  |  |  |  | Cellophane | 76.04 | Cellulose | 0.94 |  |
|  |  |  |  | Cellulose | 75.03 |  |  |  |
|  |  |  |  | Cotton grey fiber | 72.21 | Methyl cellulose | 0.93 |  |
|  |  |  |  | Cotton white lab coat fiber | 70.23 |  |  |  |
|  |  |  |  | Cotton purple fiber | 69.93 | Hydroxyethyl cellulose | 0.93 |  |
|  |  |  |  | Mask 128S GeATR #2 | 69.12 |  |  |  |
| PS-S | 5a | Fiber | Clear | Cotton brown fiber | 98.08 | Cardboard/ Cellulose | 0.97 | Anthropogenically impacted |
|  |  |  |  | Cotton blue fiber | 97.73 |  |  |  |
|  |  |  |  | Cotton Yellow Fiber | 96.47 | Papercup cellulosic | 0.96 |  |
|  |  |  |  | Cotton black fiber | 95.80 |  |  |  |
|  |  |  |  | Mask 128S GeATR #2 | 95.69 | Cellulose | 0.95 |  |
|  |  |  |  | Cotton purple fiber | 95.52 |  |  |  |
|  |  |  |  | Cotton blue fiber | 95.43 | Cellulose | 0.94 |  |
|  |  |  |  | Cotton pink fiber bundle | 94.05 |  |  |  |
|  |  |  |  | Cotton blue fiber | 93.75 | .Cellulose wipe | 0.93 |  |
|  |  |  |  | Cellulose acetate filter | 92.93 |  |  |  |
| PS-S | 5b | Fiber | Clear | Cotton purple fiber | 96.54 | Cardboard/ Cellulose | 0.97 | Anthropogenically impacted |
|  |  |  |  | Cotton blue fiber | 95.81 |  |  |  |
|  |  |  |  | Mask 128S GeATR #2 | 95.61 | Papercup cellulosic | 0.96 |  |
|  |  |  |  | Cotton brown fiber | 95.17 |  |  |  |
|  |  |  |  | Cotton green fiber | 95.17 | Cellulose | 0.91 |  |
|  |  |  |  | Cotton Yellow Fiber | 95.07 |  |  |  |
|  |  |  |  | Cotton Yellow Fiber | 94.64 | Cellulose | 0.91 |  |
|  |  |  |  | Cotton blue fiber | 93.28 |  |  |  |
|  |  |  |  | Cotton grey fiber | 92.82 | .Cellulose wipe | 0.91 |  |
|  |  |  |  | Cotton black fiber | 92.77 |  |  |  |
| PS-S | 6a | Fiber | Blue | Cotton pink fiber | 86.09 | Cardboard/ Cellulose | 0.97 | Semi-Synthetic |
|  |  |  |  | Cotton green fiber | 82.01 |  |  |  |
|  |  |  |  | Cellulose | 81.49 | Papercup cellulosic | 0.96 |  |
|  |  |  |  | Cotton Yellow Fiber | 80.23 |  |  |  |
|  |  |  |  | Cotton grey fiber | 79.42 | Hydroxyethyl cellulose | 0.91 |  |
|  |  |  |  | Cellophane | 76.95 |  |  |  |
|  |  |  |  | Cotton grey fiber | 74.09 | Methyl cellulose | 0.91 |  |
|  |  |  |  | Fibre viscose | 73.39 |  |  |  |
|  |  |  |  | Fibre viscose dyed | 72.26 | Cellulose | 0.91 |  |
|  |  |  |  | Wood mahagoni | 71.59 |  |  |  |
| PS-S | 6b | Fiber | Clear | Cotton Yellow Fiber | 94.33 | Cardboard/ Cellulose | 0.97 | Anthropogenically impacted |
|  |  |  |  | Cotton green fiber | 92.86 |  |  |  |
|  |  |  |  | Cotton pink fiber | 89.95 | Papercup cellulosic | 0.96 |  |
|  |  |  |  | Cotton grey fiber | 89.87 |  |  |  |
|  |  |  |  | Cotton purple fiber | 89.61 | Methyl cellulose | 0.91 |  |
|  |  |  |  | Cotton blue fiber | 88.50 |  |  |  |
|  |  |  |  | Cotton brown fiber | 87.65 | Hydroxyethyl cellulose | 0.91 |  |
|  |  |  |  | Cotton Yellow Fiber | 87.33 |  |  |  |
|  |  |  |  | Cotton grey fiber | 87.30 | Cellulose | 0.91 |  |
|  |  |  |  | Cotton blue fiber | 86.99 |  |  |  |
| PS-S | 8a | Fiber | Clear | Cotton blue fiber | 94.67 | Cardboard/ Cellulose | 0.98 | Anthropogenically impacted |
|  |  |  |  | Cotton Yellow Fiber | 94.10 |  |  |  |
|  |  |  |  | Cotton black fiber | 93.42 | Papercup cellulosic | 0.97 |  |
|  |  |  |  | Mask 128S GeATR #2 | 93.41 |  |  |  |
|  |  |  |  | Cotton purple fiber | 92.92 | Cellulose | 0.92 |  |
|  |  |  |  | Cotton brown fiber | 92.92 |  |  |  |
|  |  |  |  | Cotton blue fiber | 92.91 | Hydroxyethyl cellulose | 0.92 |  |
|  |  |  |  | Cotton Yellow Fiber | 92.00 |  |  |  |
|  |  |  |  | Cotton green fiber | 91.74 | Methyl cellulose | 0.92 |  |
|  |  |  |  | Cotton blue fiber | 91.17 |  |  |  |
| PS-S | 8b | Fiber | Clear | Cotton Yellow Fiber | 96.77 | Cardboard/ Cellulose | 0.97 | Anthropogenically impacted |
|  |  |  |  | Cotton green fiber | 96.12 |  |  |  |
|  |  |  |  | Cotton pink fiber | 94.40 | Papercup cellulosic | 0.97 |  |
|  |  |  |  | Cotton grey fiber | 93.84 |  |  |  |
|  |  |  |  | Cotton purple fiber | 91.76 | Cellulose | 0.91 |  |
|  |  |  |  | Cotton grey fiber | 89.30 |  |  |  |
|  |  |  |  | Cotton blue fiber | 89.22 | Methyl cellulose | 0.91 |  |
|  |  |  |  | Cotton brown fiber | 88.68 |  |  |  |
|  |  |  |  | Cotton white lab coat fiber | 88.45 | Cellulose | 0.91 |  |
|  |  |  |  | Cotton Yellow Fiber | 88.40 |  |  |  |
| PS-S | 9a | Fiber | Clear | Cotton blue fiber | 96.22 | Cardboard/ Cellulose | 0.98 | Anthropogenically impacted |
|  |  |  |  | Cotton brown fiber | 96.18 |  |  |  |
|  |  |  |  | Cotton blue fiber | 94.60 | Papercup cellulosic | 0.97 |  |
|  |  |  |  | Cotton purple fiber | 94.48 |  |  |  |
|  |  |  |  | Cotton Yellow Fiber | 94.32 | Cellulose | 0.94 |  |
|  |  |  |  | Mask 128S GeATR #2 | 94.18 |  |  |  |
|  |  |  |  | Cotton Yellow Fiber | 93.80 | Cellulose | 0.93 |  |
|  |  |  |  | Cotton black fiber | 93.63 |  |  |  |
|  |  |  |  | Cotton blue fiber | 92.88 | Hydroxyethyl cellulose | 0.92 |  |
|  |  |  |  | Cotton pink fiber bundle | 92.79 |  |  |  |
| PS-S | 9b | Fiber | Clear | Cotton blue fiber | 98.02 | Cardboard/ Cellulose | 0.97 | Anthropogenically impacted |
|  |  |  |  | Cotton brown fiber | 97.97 |  |  |  |
|  |  |  |  | Cotton black fiber | 96.73 | Papercup cellulosic | 0.96 |  |
|  |  |  |  | Cotton Yellow Fiber | 96.65 |  |  |  |
|  |  |  |  | Mask 128S GeATR #2 | 96.40 | Cellulose | 0.92 |  |
|  |  |  |  | Cotton blue fiber | 95.93 |  |  |  |
|  |  |  |  | Cotton purple fiber | 95.23 | Cellulose | 0.92 |  |
|  |  |  |  | Cotton pink fiber bundle | 94.79 |  |  |  |
|  |  |  |  | Cotton blue fiber | 94.46 | Fibre poplar down | 0.91 |  |
|  |  |  |  | Cotton green fiber | 92.88 |  |  |  |
| PS-S | 10a | Fiber | Black | Cotton pink fiber | 88.39 | Cardboard/ Cellulose | 0.98 | Semi-Synthetic |
|  |  |  |  | Cotton green fiber | 85.35 |  |  |  |
|  |  |  |  | Cotton Yellow Fiber | 84.56 | Papercup cellulosic | 0.97 |  |
|  |  |  |  | Cotton grey fiber | 82.94 |  |  |  |
|  |  |  |  | Cellulose | 79.72 | Cellulose | 0.94 |  |
|  |  |  |  | Cotton grey fiber | 76.71 |  |  |  |
|  |  |  |  | Cellophane | 76.52 | Cellulose | 0.93 |  |
|  |  |  |  | Cotton purple fiber | 75.83 |  |  |  |
|  |  |  |  | Fibre viscose | 75.47 | Hydroxyethyl cellulose | 0.93 |  |
|  |  |  |  | Cotton white lab coat fiber | 74.35 |  |  |  |
| PS-S | 10b | Fiber | Clear | Mask 128S GeATR #2 | 97.87 | Cardboard/ Cellulose | 0.98 | Anthropogenically impacted |
|  |  |  |  | Cotton blue fiber | 97.79 |  |  |  |
|  |  |  |  | Cotton Yellow Fiber | 97.36 | Papercup cellulosic | 0.97 |  |
|  |  |  |  | Cotton brown fiber | 97.03 |  |  |  |
|  |  |  |  | Cotton black fiber | 96.75 | Cellulose | 0.93 |  |
|  |  |  |  | Cotton blue fiber | 95.37 |  |  |  |
|  |  |  |  | Cotton purple fiber | 94.62 | Cellulose | 0.93 |  |
|  |  |  |  | Cotton blue fiber | 94.25 |  |  |  |
|  |  |  |  | Cotton green fiber | 93.87 | Fibre poplar down | 0.92 |  |
|  |  |  |  | Cotton Yellow Fiber | 93.21 |  |  |  |
| PS-S | 11a | Fiber | Blue | Mask 128S GeATR #2 | 93.85 | Cardboard/ Cellulose | 0.97 | Anthropogenically impacted |
|  |  |  |  | Cotton black fiber | 93.18 |  |  |  |
|  |  |  |  | Cotton Yellow Fiber | 92.40 | Papercup cellulosic | 0.97 |  |
|  |  |  |  | Cotton blue fiber | 90.73 |  |  |  |
|  |  |  |  | Cotton pink fiber bundle | 90.08 | Cellulose | 0.93 |  |
|  |  |  |  | Cotton green fiber | 89.93 |  |  |  |
|  |  |  |  | Cotton brown fiber | 89.61 | Cellulose | 0.93 |  |
|  |  |  |  | Cotton blue fiber | 87.51 |  |  |  |
|  |  |  |  | Cotton blue fiber | 87.13 | Hydroxyethyl cellulose | 0.92 |  |
|  |  |  |  | Cotton black fiber | 87.02 |  |  |  |
| PS-S | 11b | Fiber | Yellow | Cotton blue fiber | 94.87 | Cardboard/ Cellulose | 0.98 | Anthropogenically impacted |
|  |  |  |  | Mask 128S GeATR #2 | 94.07 |  |  |  |
|  |  |  |  | Cotton Yellow Fiber | 93.75 | Papercup cellulosic | 0.97 |  |
|  |  |  |  | Cotton brown fiber | 93.38 |  |  |  |
|  |  |  |  | Cotton purple fiber | 92.93 | Cellulose | 0.92 |  |
|  |  |  |  | Cotton black fiber | 92.76 |  |  |  |
|  |  |  |  | Cotton blue fiber | 92.70 | Cellulose | 0.92 |  |
|  |  |  |  | Cotton green fiber | 92.60 |  |  |  |
|  |  |  |  | Cotton Yellow Fiber | 92.46 | Fibre poplar down | 0.92 |  |
|  |  |  |  | Cotton blue fiber | 91.70 |  |  |  |
| PS-S | 12a | Fiber | Blue | Cotton brown fiber | 97.60 | Cardboard/ Cellulose | 0.98 | Anthropogenically impacted |
|  |  |  |  | Cotton blue fiber | 97.57 |  |  |  |
|  |  |  |  | Mask 128S GeATR #2 | 96.85 | Papercup cellulosic | 0.97 |  |
|  |  |  |  | Cotton purple fiber | 96.68 |  |  |  |
|  |  |  |  | Cotton Yellow Fiber | 96.14 | Cellulose | 0.95 |  |
|  |  |  |  | Cotton blue fiber | 95.57 |  |  |  |
|  |  |  |  | Cotton Yellow Fiber | 95.15 | Cellulose | 0.95 |  |
|  |  |  |  | Cotton green fiber | 94.88 |  |  |  |
|  |  |  |  | Cotton black fiber | 94.46 | .Cellulose wipe | 0.93 |  |
|  |  |  |  | Cotton blue fiber | 93.70 |  |  |  |
| PS-S | 12b | Fiber | Clear | Mask 128S GeATR #2 | 92.31 | Cardboard/ Cellulose | 0.98 | Anthropogenically impacted |
|  |  |  |  | Cotton green fiber | 89.96 |  |  |  |
|  |  |  |  | Cotton blue fiber | 88.00 | Papercup cellulosic | 0.97 |  |
|  |  |  |  | Cotton purple fiber | 87.67 |  |  |  |
|  |  |  |  | Cotton Yellow Fiber | 87.63 | .Cellulose wipe | 0.96 |  |
|  |  |  |  | Cotton brown fiber | 87.54 |  |  |  |
|  |  |  |  | Cotton grey fiber | 87.16 | Cellulose | 0.96 |  |
|  |  |  |  | Cotton pink fiber | 87.01 |  |  |  |
|  |  |  |  | Fibre linen | 86.32 | Cellulose | 0.96 |  |
|  |  |  |  | Cotton Yellow Fiber | 86.31 |  |  |  |
| PS-S | 15a | Fiber | Clear | Cotton brown fiber | 98.55 | Cardboard/ Cellulose | 0.98 | Anthropogenically impacted |
|  |  |  |  | Cotton blue fiber | 98.38 |  |  |  |
|  |  |  |  | Cotton purple fiber | 97.39 | Papercup cellulosic | 0.97 |  |
|  |  |  |  | Mask 128S GeATR #2 | 97.36 |  |  |  |
|  |  |  |  | Cotton Yellow Fiber | 96.14 | Cellulose | 0.94 |  |
|  |  |  |  | Cotton blue fiber | 96.02 |  |  |  |
|  |  |  |  | Cotton blue fiber | 94.89 | Cellulose | 0.94 |  |
|  |  |  |  | Cotton Yellow Fiber | 94.68 |  |  |  |
|  |  |  |  | Cotton black fiber | 94.67 | .Cellulose wipe | 0.93 |  |
|  |  |  |  | Cotton green fiber | 94.10 |  |  |  |
| PS-S | 15b | Fiber | Clear | Mask 128S GeATR #2 | 96.55 | Cardboard/ Cellulose | 0.98 | Anthropogenically impacted |
|  |  |  |  | Cotton blue fiber | 96.31 |  |  |  |
|  |  |  |  | Cotton Yellow Fiber | 95.91 | Papercup cellulosic | 0.97 |  |
|  |  |  |  | Cotton black fiber | 94.91 |  |  |  |
|  |  |  |  | Cotton brown fiber | 94.71 | Cellulose | 0.93 |  |
|  |  |  |  | Cotton blue fiber | 94.59 |  |  |  |
|  |  |  |  | Cotton purple fiber | 94.02 | Cellulose | 0.92 |  |
|  |  |  |  | Cotton Yellow Fiber | 93.04 |  |  |  |
|  |  |  |  | Cotton green fiber | 92.91 | Hydroxyethyl cellulose | 0.92 |  |
|  |  |  |  | Cotton blue fiber | 92.66 |  |  |  |
| PS-S | 17a | Fiber | Grey | Cotton brown fiber | 98.56 | Cardboard/ Cellulose | 0.97 | Anthropogenically impacted |
|  |  |  |  | Cotton blue fiber | 98.40 |  |  |  |
|  |  |  |  | Cotton purple fiber | 96.70 | Papercup cellulosic | 0.96 |  |
|  |  |  |  | Cotton Yellow Fiber | 96.09 |  |  |  |
|  |  |  |  | Cotton blue fiber | 95.99 | Cellulose | 0.94 |  |
|  |  |  |  | Mask 128S GeATR #2 | 95.66 |  |  |  |
|  |  |  |  | Cotton black fiber | 95.48 | Cellulose | 0.94 |  |
|  |  |  |  | Cotton blue fiber | 94.72 |  |  |  |
|  |  |  |  | Cotton pink fiber bundle | 93.63 | Fibre poplar down | 0.92 |  |
|  |  |  |  | Cotton Yellow Fiber | 93.41 |  |  |  |
| PS-S | 17b | Fiber | Clear | Mask 128S GeATR #2 | 95.83 | Cardboard/ Cellulose | 0.97 | Anthropogenically impacted |
|  |  |  |  | Cotton Yellow Fiber | 94.81 |  |  |  |
|  |  |  |  | Cotton black fiber | 94.03 | Papercup cellulosic | 0.97 |  |
|  |  |  |  | Cotton blue fiber | 93.44 |  |  |  |
|  |  |  |  | Cotton green fiber | 92.87 | Cellulose | 0.95 |  |
|  |  |  |  | Cotton brown fiber | 92.40 |  |  |  |
|  |  |  |  | Cotton blue fiber | 90.51 | Cellulose | 0.94 |  |
|  |  |  |  | Cotton white lab coat fiber | 90.16 |  |  |  |
|  |  |  |  | Cotton blue fiber | 89.93 | Hydroxyethyl cellulose | 0.93 |  |
|  |  |  |  | Cotton purple fiber | 89.50 |  |  |  |
| PS-S | 20a | Fiber | Blue | Polyester pink fiber | 96.62 | Polyesterterpthalate | 0.96 | Synthetic |
|  |  |  |  | Polyester white fiber | 96.48 |  |  |  |
|  |  |  |  | PET blue fragment | 96.34 | Polyester | 0.96 |  |
|  |  |  |  | PET black fiber | 96.18 |  |  |  |
|  |  |  |  | PET green fiber | 95.92 | PET | 0.95 |  |
|  |  |  |  | Polyester red yarn fiber | 95.51 |  |  |  |
|  |  |  |  | PET green fiber | 95.34 | Polyethylene terephthalate | 0.95 |  |
|  |  |  |  | Polyester brown yarn fiber | 95.31 |  |  |  |
|  |  |  |  | PET white fiber bundle | 95.27 | Polyesterterpthalate | 0.95 |  |
|  |  |  |  | Polyester yellow fiber | 95.06 |  |  |  |
| PS-S | 20b | Fiber | Clear | Cotton pink fiber | 85.09 | Cardboard/ Cellulose | 0.97 | Semi-Synthetic |
|  |  |  |  | Cellophane | 80.59 |  |  |  |
|  |  |  |  | Cotton green fiber | 80.33 | Papercup cellulosic | 0.97 |  |
|  |  |  |  | Cotton Yellow Fiber | 79.65 |  |  |  |
|  |  |  |  | Cotton grey fiber | 78.76 | Methyl cellulose | 0.93 |  |
|  |  |  |  | Cellulose | 78.53 |  |  |  |
|  |  |  |  | Cotton grey fiber | 72.62 | Hydroxyethyl cellulose | 0.92 |  |
|  |  |  |  | Fibre viscose dyed | 71.00 |  |  |  |
|  |  |  |  | Fibre viscose | 70.87 | Hydroxyethyl cellulose | 0.91 |  |
|  |  |  |  | Cotton white lab coat fiber | 69.73 |  |  |  |
| PS-S | 22a | Fiber | Blue | Cotton brown fiber | 97.63 | Cardboard/ Cellulose | 0.97 | Anthropogenically impacted |
|  |  |  |  | Cotton blue fiber | 97.50 |  |  |  |
|  |  |  |  | Cotton black fiber | 96.15 | Papercup cellulosic | 0.97 |  |
|  |  |  |  | Cotton Yellow Fiber | 96.01 |  |  |  |
|  |  |  |  | Mask 128S GeATR #2 | 95.99 | Cellulose | 0.95 |  |
|  |  |  |  | Cotton blue fiber | 95.84 |  |  |  |
|  |  |  |  | Cotton pink fiber bundle | 94.18 | Cellulose | 0.94 |  |
|  |  |  |  | Cotton blue fiber | 93.93 |  |  |  |
|  |  |  |  | Cotton purple fiber | 93.90 | Fibre poplar down | 0.92 |  |
|  |  |  |  | Cotton green fiber | 93.03 |  |  |  |
| PS-S | 22b | Fiber | Clear | Cotton brown fiber | 98.49 | Cardboard/ Cellulose | 0.97 | Anthropogenically impacted |
|  |  |  |  | Cotton blue fiber | 97.81 |  |  |  |
|  |  |  |  | Cotton purple fiber | 96.95 | Papercup cellulosic | 0.96 |  |
|  |  |  |  | Cotton blue fiber | 95.87 |  |  |  |
|  |  |  |  | Cotton Yellow Fiber | 95.27 | Cellulose | 0.93 |  |
|  |  |  |  | Mask 128S GeATR #2 | 94.71 |  |  |  |
|  |  |  |  | Cotton black fiber | 94.33 | Cellulose | 0.93 |  |
|  |  |  |  | Cotton blue fiber | 94.04 |  |  |  |
|  |  |  |  | Cotton Yellow Fiber | 93.33 | .Cellulose wipe | 0.92 |  |
|  |  |  |  | Cotton pink fiber bundle | 92.87 |  |  |  |
| PS-S | 23a | Fiber | Clear | Cotton pink fiber bundle | 97.01 | Cardboard/ Cellulose | 0.97 | Anthropogenically impacted |
|  |  |  |  | Cotton black fiber | 96.84 |  |  |  |
|  |  |  |  | Cotton blue fiber | 96.41 | Papercup cellulosic | 0.96 |  |
|  |  |  |  | Cotton Yellow Fiber | 95.99 |  |  |  |
|  |  |  |  | Cotton brown fiber | 95.88 | Cellulose | 0.93 |  |
|  |  |  |  | Cotton blue fiber | 94.52 |  |  |  |
|  |  |  |  | Mask 128S GeATR #2 | 93.77 | Cellulose | 0.92 |  |
|  |  |  |  | Cotton blue fiber | 92.83 |  |  |  |
|  |  |  |  | Cotton green fiber | 92.59 | Hydroxyethyl cellulose | 0.91 |  |
|  |  |  |  | Cotton purple fiber | 91.61 |  |  |  |
| PS-S | 23b | Fiber | Grey | Mask 128S GeATR #2 | 90.58 | Cardboard/ Cellulose | 0.96 | Anthropogenically impacted |
|  |  |  |  | Cotton black fiber | 90.18 |  |  |  |
|  |  |  |  | Cotton Yellow Fiber | 89.98 | Papercup cellulosic | 0.95 |  |
|  |  |  |  | Cotton blue fiber | 89.53 |  |  |  |
|  |  |  |  | Cotton brown fiber | 88.93 | Cellulose | 0.91 |  |
|  |  |  |  | Cotton pink fiber bundle | 88.27 |  |  |  |
|  |  |  |  | Cotton green fiber | 87.70 | Cellulose | 0.91 |  |
|  |  |  |  | Cotton blue fiber | 86.47 |  |  |  |
|  |  |  |  | Cotton blue fiber | 86.31 | Methyl cellulose | 0.91 |  |
|  |  |  |  | Cotton black fiber | 86.05 |  |  |  |
| PS-S | 27a | Fiber | Yellow | Wood mahagoni | 82.69 | Cardboard/ Cellulose | 0.97 | Natural |
|  |  |  |  | Cotton green fiber | 80.36 |  |  |  |
|  |  |  |  | Fibre poplar down | 80.05 | Papercup cellulosic | 0.97 |  |
|  |  |  |  | Cotton Yellow Fiber | 79.21 |  |  |  |
|  |  |  |  | Mask 128S GeATR #2 | 79.18 | Methyl cellulose | 0.93 |  |
|  |  |  |  | Cotton blue fiber | 79.18 |  |  |  |
|  |  |  |  | Fibre kapok | 78.24 | Hydroxyethyl cellulose | 0.92 |  |
|  |  |  |  | Fibre poplar down | 78.07 |  |  |  |
|  |  |  |  | Cotton blue fiber | 78.04 | Cellulose | 0.92 |  |
|  |  |  |  | Cotton purple fiber | 77.80 |  |  |  |
| PS-S | 27b | Fiber | Clear | Mask 128S GeATR #2 | 95.10 | Cardboard/ Cellulose | 0.98 | Semi-Synthetic |
|  |  |  |  | Cotton Yellow Fiber | 93.69 |  |  |  |
|  |  |  |  | Cotton green fiber | 93.60 | Papercup cellulosic | 0.97 |  |
|  |  |  |  | Cotton blue fiber | 93.53 |  |  |  |
|  |  |  |  | Cotton Yellow Fiber | 93.34 | Cellulose | 0.94 |  |
|  |  |  |  | Cotton purple fiber | 92.62 |  |  |  |
|  |  |  |  | Cotton brown fiber | 92.39 | Cellulose | 0.93 |  |
|  |  |  |  | Cotton blue fiber | 91.85 |  |  |  |
|  |  |  |  | Cotton black fiber | 91.50 | Hydroxyethyl cellulose | 0.93 |  |
|  |  |  |  | Cotton grey fiber | 90.68 |  |  |  |
| BR-S | 5a | Fiber | Black | PET white fiber bundle | 97.19 | Polyethylene terephthalate | 0.97 | Synthetic |
|  |  |  |  | Polyester white label fiber | 96.85 |  |  |  |
|  |  |  |  | PET white fragment | 96.84 | Polyesterterpthalate | 0.97 |  |
|  |  |  |  | PET blue fragment | 96.74 |  |  |  |
|  |  |  |  | PET Yellow fiber bundle | 96.15 | Polyethylene terephthalate | 0.97 |  |
|  |  |  |  | PET orange fiber | 95.96 |  |  |  |
|  |  |  |  | PET white fiber bundle | 95.92 | Polyethylene terephthalate | 0.97 |  |
|  |  |  |  | PET blue fiber bundle | 95.50 |  |  |  |
|  |  |  |  | PET green fiber | 95.46 | Polyethylene terephthalate | 0.96 |  |
|  |  |  |  | Polyester blue yarn fiber | 95.42 |  |  |  |
| BR-S | 5b | Fiber | Black | Polyethylene terephthalate | 95.90 | Polyethylene terephthalate | 0.95 | Synthetic |
|  |  |  |  | Polyester red fiber | 95.24 |  |  |  |
|  |  |  |  | Polyester | 95.04 | Polyethylene terephthalate | 0.95 |  |
|  |  |  |  | Polyethylene terephthalate | 94.81 |  |  |  |
|  |  |  |  | Polyester red yarn fiber | 94.60 | Polybuthylene terephthalate | 0.94 |  |
|  |  |  |  | PET white fragment | 94.43 |  |  |  |
|  |  |  |  | Polyethylene terephthalate | 94.42 | PET | 0.94 |  |
|  |  |  |  | PET green glitter fragment | 94.30 |  |  |  |
|  |  |  |  | Polyester white fabric bag fiber | 94.14 | Polyethylene terephthalate | 0.94 |  |
|  |  |  |  | Polyester blue yarn fiber | 93.85 |  |  |  |
| BR-S | 16a | Fiber | Black | PET white fragment | 97.79 | Polyesterterpthalate | 0.97 | Synthetic |
|  |  |  |  | Polyethylene terephthalate | 96.93 |  |  |  |
|  |  |  |  | PET Yellow fiber bundle | 96.74 | Polyethylene terephthalate | 0.97 |  |
|  |  |  |  | Polyester white label fiber | 96.70 |  |  |  |
|  |  |  |  | Polyester red yarn fiber | 96.54 | PET | 0.96 |  |
|  |  |  |  | Polyester blue yarn fiber | 96.41 |  |  |  |
|  |  |  |  | Polyester green label fiber | 96.28 | Polyethylene terephthalate | 0.96 |  |
|  |  |  |  | PET black fragment | 95.98 |  |  |  |
|  |  |  |  | PET green glitter fragment | 95.76 | Polyethylene terephthalate | 0.96 |  |
|  |  |  |  | Polyester red fiber | 95.54 |  |  |  |
| BR-S | 16b | Fiber | Clear | Cotton pink fiber | 88.88 | Cardboard/ Cellulose | 0.98 | Semi-Synthetic |
|  |  |  |  | Cotton green fiber | 84.68 |  |  |  |
|  |  |  |  | Cellophane | 83.87 | Papercup cellulosic | 0.97 |  |
|  |  |  |  | Cotton grey fiber | 83.25 |  |  |  |
|  |  |  |  | Cellulose | 83.24 | Cellulose | 0.94 |  |
|  |  |  |  | Cotton Yellow Fiber | 82.17 |  |  |  |
|  |  |  |  | Cotton grey fiber | 76.98 | Hydroxyethyl cellulose | 0.93 |  |
|  |  |  |  | Fibre grass | 76.35 |  |  |  |
|  |  |  |  | Fibre viscose | 96.33 | Cellulose | 0.93 |  |
|  |  |  |  | Fibre viscose dyed | 95.49 |  |  |  |
| BR-S | 17a | Fiber | Clear | Cotton pink fiber | 88.96 | Cardboard/ Cellulose | 0.98 | Anthropogenically impacted |
|  |  |  |  | Cotton green fiber | 84.13 |  |  |  |
|  |  |  |  | Cellulose | 82.99 | Papercup cellulosic | 0.97 |  |
|  |  |  |  | Cotton grey fiber | 82.42 |  |  |  |
|  |  |  |  | Cotton Yellow Fiber | 81.53 | Cellulose | 0.92 |  |
|  |  |  |  | Cellophane | 79.71 |  |  |  |
|  |  |  |  | Fibre grass | 78.85 | Hydroxyethyl cellulose | 0.92 |  |
|  |  |  |  | Cotton grey fiber | 77.24 |  |  |  |
|  |  |  |  | Fibre viscose | 76.84 | Methyl cellulose | 0.92 |  |
|  |  |  |  | Wood mahagoni | 75.83 |  |  |  |
| BR-S | 17b | Fiber | Blue | Polyester white label fiber | 97.59 | Polyesterterpthalate | 0.96 | Synthetic |
|  |  |  |  | PET white fragment | 97.50 |  |  |  |
|  |  |  |  | Polyester blue yarn fiber | 97.46 | Polyethylene terephthalate | 0.96 |  |
|  |  |  |  | PET Yellow fiber bundle | 97.38 |  |  |  |
|  |  |  |  | Polyester green label fiber | 97.25 | Polyethylene terephthalate | 0.96 |  |
|  |  |  |  | Polyester red fiber | 97.00 |  |  |  |
|  |  |  |  | Polyester red yarn fiber | 96.97 | Polyethylene terephthalate | 0.95 |  |
|  |  |  |  | PET black fiber | 96.74 |  |  |  |
|  |  |  |  | Polyester dark brown yarn fiber | 96.70 | PET | 0.95 |  |
|  |  |  |  | PET blue fiber bundle | 96.49 |  |  |  |

Table D. Microplastics/gram of edible tissue from **Traylor et al. (this paper, bold)** and other studies that report edible tissue in items/gram. Species, organism type, sample location, items/gram of tissue and source are included.

| **Species** | **Organism type** | **Location of collection** | **items/g tissue** | **Source** |
| --- | --- | --- | --- | --- |
| ***Ophiodon elongatus-(v)*** | **Lingcod** | **Oregon coast** | **0.022** | **Traylor et al. (2025)** |
| ***Oncorhynchus tshawytscha*** | **Chinook** | **Oregon coast** | **0.028** | **Traylor et al. (2025)** |
| *Konosirus punctatus* | finfish | East China Sea | 0.044 | Wu et al. (2020) |
| ***Ophiodon elongatus (r)*** | **Lingcod** | **Oregon coast** | **0.091** | **Traylor et al. (2025)** |
| ***Sebastes melanops*** | **Black Rockfish** | **Oregon coast** | **0.107** | **Traylor et al. (2025)** |
| *Epinephelus coioides* | grouper | Persian Gulf | 0.158 | Akhbarizadeh et al. (2019) |
| *Cynoglossus abbreviatus* | finfish | Persian Gulf | 0.16 | Abbasi et al. (2018) |
| *Parapenaeopsis hardwickii* | Spear shrimp | East China Sea | 0.25 | Wu et al. (2020) |
| *Scomberomorus guttatus* | King Mackerel | Bangladesh estuary | 0.3 | Hossain et al. (2023) |
| *Scomber Scombrus* | Mackerel | Scotland -Atlantic | 0.3 | Akoueson et al. (2020) |
| *Penaeus semisulcatus* | Green tiger prawn | Persian Gulf | 0.36 | Akhbarizadeh et al. (2019) |
| *Dicentrachus labrax* | fish | W Portugal coast | 0.4 | Barboza et al (2020) |
| *Scomber colia* | fish | W Portugal coast | 0.6 | Barboza et al (2020) |
| ***Entosphenus tridentatus (ad)*** | **lamprey** | **Oregon coast** | **0.604** | **Traylor et al. (2025)** |
| *Trachurus trachurus* | fish | W Portugal coast | 0.7 | Barboza et al (2020) |
| ***Entosphenus tridentatus (juv)*** | **lamprey** | **Oregon coast** | **0.999** | **Traylor et al. (2025)** |
| ***Clupea pallasii*** | **Pacific Herring** | **Oregon coast** | **1.08** | **Traylor et al. (2025)** |
| *Penaeus semisulcatus* | Green tiger prawn | Persian Gulf | 1.5 | Abbasi et al. (2018) |
| *Litopenaeus vannameiin* | Pacific white shrimp | Caspian Sea | 5.7 | Bagheri et al. (2020) |
| ***Pandalus jordani (r)*** | **Pink Shrimp** | **Oregon coast** | **7.62** | **Traylor et al. (2025)** |
| ***Pandalus jordani (v)*** | **Pink Shrimp** | **Oregon coast** | **10.67** | **Traylor et al. (2025)** |
| *Paratya australiensis* | Glass shrimp | Eastern Australia | 24 | Nan et al. (2020) |

Appendix Figure A**. a) Control sample material, b) seafood sample material, and c) seafood sample color categories of particles found using FTIR.**
